# Supplementary material for: Graft conditioning with fluticasone propionate reduces graft‐versus‐host disease upon allogeneic hematopoietic cell transplantation in mice
Source: EMBO Mol Med. 2023 Aug 4;15(9):e17748. doi: 10.15252/emmm.202317748 (PMC10493574; doi:10.15252/emmm.202317748)
Supplement: Supplementary file 1 — Appendix [file EMMM-15-e17748-s005.pdf]

APPENDIX

Graft pre-treatment with fluticasone propionate reduces graft-versus-host disease in allo-HCT

Erika S. Varady[1,2,*], L. Angel Ayala[1,2,*], Pauline U. Nguyen[1,2], Vanessa M. Scarfone[1], Alborz Karimzadeh[1,2,**], Cuiwen Zhou[1,2], Xiyu Chen[1,2], Scott A. Greilach[1,2], Craig M. Walsh[1,2], and Matthew A. Inlay[1,2,†]

Table of Contents

Appendix Methods 2

Appendix Table S1. Antibodies 5

Appendix References 6

Appendix Methods

Antibodies

A detailed list of all antibodies used in this study is shown in Table S1.

Mice

C57BL/6J (H-2^b, "B6", stock no. 00664), mTmG (H-2^b, "tdTomato+", stock no. 007576), B6-Foxp3^{EGFP} (H-2^b, "FoxP3-GFP", stock no. 006772) and Balb/cJ (H-2^d, "Balb/c", stock no. 000651) mouse strains from Jackson Laboratory (Bar Harbor, ME) 8-12 weeks of age along with Rosa-ECFP (H-2^b, "CFP" or "TM5") mice 8-12 weeks of age generously donated by Dr. Irving Weissman, were used as donors and recipients for competitive transplantation. mTmG, FoxP3-GFP, and CFP are all congenic strains on the B6 background.

HSC Cell Culture for CXCR4 analysis

BM was harvested from C57BL/6 mice by crushing tibias and femurs using a mortar and pestle. Cells were processed in ice-cold fluorescence activated cell sorting (FACS) buffer (phosphate buffered saline (PBS) + 2% fetal bovine serum (FBS)). Red blood cells were lysed by ACK lysis buffer (155 mM NH_4Cl, 10 mM $KHCO_3$, 0.1 mM Na_2EDTA, pH 7.2-7.4 in dH2O) then cells were filtered through a 70μ mesh (Genesee Scientific, CA, #57-104). Cells were then resuspended in 100μL FACS buffer and incubated with 5μL of mouse anti-Kit (anti-CD117) Microbeads (Miltenyi Biotec, #130-091-224) for 15 minutes then magnetic separation was performed using the Milyenyi AutoMACs Pro cell separator (Miltenyi Biotec, Somerville, MA). Approximately 500,000 cells from the CD117 positive fraction were cultured per well in a 24-well culture plate in 500μL of X-VIVO 15 cell culture media (Fisher Scientific, #BW04-418Q) with 50 ng/mL murine stem cell factor (SCF) (PeproTech, #250-03) and 20 ng/mL murine thrombopoietin (TPO) (PeproTech, #315-14). Fluticasone propionate (FLU) (Sigma-Aldrich, F9428) was cultured at a concentration of 3nM unless otherwise specified. FLU and dexamethasone (abcam, ab120743) were reconstituted in DMSO. To block glucocorticoid receptor (GR), 1μM Mifepristone (RU486) (Sigma-Aldrich, #M8046) was simultaneously cultured with cells and FLU. Poly vinyl alcohol (PVA) (Sigma-Aldrich, #P8136) culture media was taken from (Wilkinson *et al*, 2019). All cells were cultured for 16 h in 37°C and 5% CO_2. After incubation, cells were analyzed using flow cytometry with antibodies listed in Appendix Table S1 ("Antibodies Table") in FACS buffer.

Chemotaxis Assay

Transwell migration assays were performed in 24 well-6.5mm diameter and 5.0µm pore size insert tissue culture treated transwell plates (Corning, #3421). After HSC cell culture with FLU or Vehicle culture, cells from each condition were washed and resuspended in 100µL X-VIVO 15 plus 50 ng/mL SCF and 20 ng/mL TPO culture media lacking FLU. Cells were transferred into transwell porous inserts resting in a bottom well containing 650 µL prewarmed X-VIVO 15/SCF/TPO media and 50 ng/mL mouse recombinant SDF-1 alpha (CXCL12) (Stem Cell Technologies, #78121). Transwells were cultured for 5 h in 37ºC and 5% CO_2. To block CXCR4, cells were pre-treated with 10 µg/mL AMD3100 (Sigma-Aldrich, 239820) for 30 minutes in 500 µL of X-VIVO 15/SCF/TPO media lacking FLU. After chemotactic assay incubation, cells were analyzed using Flow cytometry using antibodies listed in Supplementary Information Table S1 ("Antibodies Table") in FACS buffer. Cells from the top insert and bottom well were stained separately and counted using Countbright beads (Thermofisher, #C36950) to determine the number of migrated HSCs.

Cell Sorting

BM was harvested from donor mice by crushing leg bones in ice-cold FACS buffer followed by red blood cell lysis by ACK lysis buffer and filtration through a 70 µ mesh to remove debris. BM was Kit-enriched using the Milyenyi AutoMACs Pro (Miltenyi Biotec, Somerville, MA). Cells were stained with antibodies listed in Supporting Information Table S1 ("Antibodies Table") in FACS buffer. Cells were then fluorescence-activated cell sorted using a BD FACS-Aria II (Becton Dickinson, Franklin Lakes, NJ) into a 96-well U bottom plate containing 100 µL of X-VIVO 15/SCF/TPO culture media with or without FLU. Donor TM5 (CFP+) and mTmG (tdTomato+) mouse-derived cells were sorted into separate wells and TM5 was further split into FLU or vehicle containing wells. mTmG cells were all sorted into media only containing vehicle. Each well contained $1x10^4$ KLS (Ter119- CD27+ c-kit+ Sca1+) gated cells.

T cell Activation Assay

A tissue culture-treated flat bottom 96-well plate (Costar) was incubated with 30 µg/ml anti-hamster IgG (Vector Laboratories) for 1 hour at 37°C. Non-stimulated condition wells were

incubated with only PBS. Wells were then washed with PBS and then incubated with 5 µg/ml anti-mouse CD3e (eBiosciences) for 2 hours at 37°C. Non-stimulated condition wells were incubated with only PBS. Freshly harvested splenocytes (1.2 x 10^6) from CFP+ C57BL/6 (TM5) mice, pre-treated in triplicate with 3nM FLU or DMSO Vehicle, were stained with 5 µM CFSE (Biolegend) at a concentration of 10×10^6 cells/mL for 20 minutes at room temperature in the dark. Splenocytes were washed then split into two samples of 0.6 x 10^6 cells in 200µL complete RPMI-1640 (C-RPMI) stimulated and unstimulated condition with and without 2 µg/ml anti-mouse CD28 (eBiosciences) respectively and plated in stimulated (with eCD3), or non-stimulated (without eCD3) wells and incubated for 4 days at 37°C and 5%CO_2. Complete RPMI (C-RPMI) media was composed of RPMI 1640 with L-Glutamine, 25 mM HEPES, 0.11 mM 2-Mercaptoethanol, 1X MEM Non-Essential Amino Acids, 1 mM sodium pyruvate, and 10% heat inactivated (HI) FBS all from Gibco and 1X Penicillin/Streptomycin from Corning. After incubation, cells were analyzed using flow cytometry with antibodies listed in Appendix Table S1 in heat-inactivated (HI) FACS buffer.

Mixed Lymphocyte Reaction

Freshly harvested splenocytes (3×10^5) from CFP+ (H-2^b) mice were pre-treated in triplicate with 3nM FLU or DMSO Vehicle then used for responder cells. Responder cells were co-cultured (1:1) with (3×10^5) irradiated (2500cGy) allogeneic Balb/c or syngeneic C57BL/6 stimulator splenocytes for 4 days in 96-well round bottom plate with 200uL of C-RPMI. C-RPMI was used throughout this experiment. After incubation, cells were analyzed using flow cytometry with antibodies listed in Appendix Table S1 in HI FACS buffer.

Statistical Analysis

Statistical analysis was performed with GraphPad Prism 5 software (La Jolla, CA). Specific statistical tests are described for each experiment in the Figure Legends.

Appendix Table S1. Antibodies

Antigen	Clone	Conjugate	Source	Catalogue #
TER119	TER119	PE/Cy5	Biolegend	116210
	TER119	BV421	Biolegend	116233
SCA1 (Ly-6A/E)	E13-161.7	PE/Cy7	eBioscience	122514
	E13-161.7	PE	Biolegend	122507
KIT (CD117)	ACK2	APC	Biolegend	135107
	2B8	APC-eFluor 780	eBioscience	47-1171-82
	2B8	BV421	Biolegend	105828
CD27	LG.7F9	eFluor 780	eBioscience	47-0271-82
	LG.7F9	APC	eBioscience	17-0271-82
CD11A	M17/4	PE/Cy7	eBioscience	25-0111-30
	M17/4	Biotin	Biolegend	101103
	M17/4	APC	Biolegend	101119
	M17/4	PE	Biolegend	101107
	M17/4	FITC	Biolegend	101106
	M17/4	Alexa Fluor 488	Biolegend	101111
EPCR (CD201)	eBio1560	PerCP-eFluor 710	eBioscience	46-2012-82
	eBio1560	APC	eBioscience	17-2012-82
GR1 (Ly-6G/Ly-6C)	RB6-8C5	Alexa Fluor 700	eBioscience	108422
MAC1 (CD11b)	M1/70	APC	Biolegend	101212
	M1/70	FITC	Biolegend	101205
CD19	6D5	APC	Biolegend	115512
	eBio1D3	PerCP-Cy5.5	eBioscience	45-0193-82
	6D5	BV421	Biolegend	115537
CD45	30-F11	APC/Cy7	Biolegend	103116
	30-F11	Alexa Fluor 700	Biolegend	103128
CD45.2	104	FITC	Biolegend	109806
CD45.1	A20	PE/Cy7	Biolegend	110729
CD3ε	17A2	PerCP-eFluor 710	eBioscience	46-0032-82
	17A2	PE/Cy7	Biolegend	100220
CD150 (SlamF1)	TC15-12F12.2	Brilliant Violet 650	Biolegend	115931
CD4	RM4-5	PE/Cy7	Biolegend	100527
CD8a	53-6.7	APC/Cy7	Biolegend	100714
CD93 (AA4.1)	AA4.1	APC	eBioscience	17-5893-81
IgD	11-26c.2a	Alexa Fluor 700	Biolegend	405729
IgM	RMM-1	APC/Cy7	Biolegend	406515
B220	RA3-6B2	BV605	Biolegend	103243
Cxcr4	L276F12	PE	Biolegend	146506
	L276F12	APC	Biologend	146508
**Secondary antibodies**				
		Qdot 655-Streptavidin	Life Technologies	Q10121MP
		Qdot 605-Streptavidin	Life Technologies	Q10103MP
		eFluor710-Streptavidin	eBioscience	49-4317-80

Appendix References

Wilkinson AC, Ishida R, Kikuchi M, *et al*. (2019) Long-term ex vivo hematopoietic stem cell expansion affords nonconditioned transplantation. *Nature*. 571(7763).
