# Supplementary material for: Graft conditioning with fluticasone propionate reduces graft‐versus‐host disease upon allogeneic hematopoietic cell transplantation in mice
Source: EMBO Mol Med. 2023 Aug 4;15(9):e17748. doi: 10.15252/emmm.202317748 (PMC10493574; doi:10.15252/emmm.202317748)
Supplement: Supplementary file 2 — Expanded View Figures PDF [file EMMM-15-e17748-s008.pdf]

Expanded View Figures

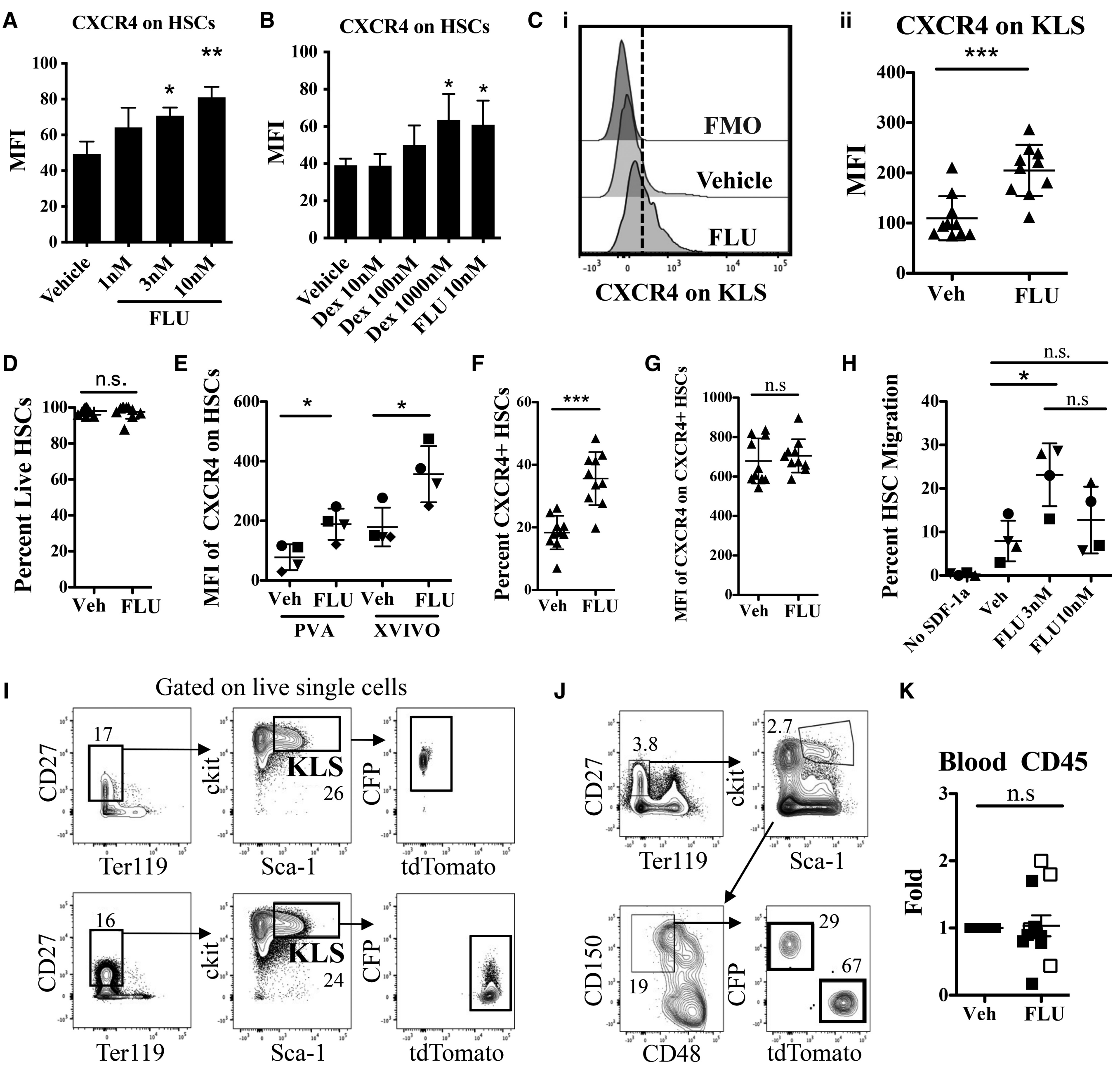

Figure EV1.

Figure EV1. Characterizing murine cells after FLU treatment.

A, B Comparisons of MFI of CXCR4 surface expression on HSCs treated with Vehicle and three different doses of (A) FLU (1, 3, and 10 nM) and (B) Dexamethasone (Dex, 10, 100, and 1,000 nM), $n = 3$. Both 3 nM and 10 nM FLU had significant increases in CXCR4 compared to vehicle, but not relative to each other. Only 1,000 nM Dex led to a significant increase in CXCR4 expression relative to vehicle, and similar to 10 nM FLU.

C (i) Representative histograms of CXCR4 expression on KLS population after FLU (blue) and Vehicle (DMSO, red) culture conditions. The dashed line indicates cutoff for positive CXCR4 expression based on the fluorescence minus one (FMO, gray histogram) control. (ii) MFI of cell surface CXCR4 on KLS population after culture conditions ($n = 10$, representative of seven independent experiments). Data indicates an approximately 2-fold increase of CXCR4 expression on KLS cells after FLU treatment relative to vehicle control similar to that observed in HSCs in Fig 1A.

D Total live HSCs after culture conditions indicates that 16 h culture in 3 nM FLU does not negatively affect HSC viability.

E MFI of CXCR4 on HSCs treated with FLU or Vehicle cultured in two different mediums, either serum-free XVIVO medium or polyvinyl alcohol (PVA) supplemented medium ($n = 4$, representative of four independent experiments). This shows that the FLU-induced increase in CXCR4 expression is not specific to one type of cell culture media.

F Percent CXCR4 positive HSCs.

G MFI of CXCR4 on CXCR4$^+$ HSC population. These data indicate that CXCR4 expression is not increasing on CXCR4$^+$ HSCs, but rather that CXCR4$^-$ HSCs begin to turn on CXCR4. D, F, and G: $n = 10$, representative of seven independent experiments.

H HSC migration toward SDF-1α (50 ng/ml) after treatment of Vehicle or two different concentrations of FLU (3 or 10 nM) quantified by flow cytometry ($n = 4$, representative of four independent experiments). Migration appeared reduced in HSCs treated in 10 nM FLU compared to 3 nM.

I Representative sorting scheme of c-kit$^+$, CD27$^+$, Sca-1$^+$ (KLS) live single cells from either CFP$^+$ (top) or tdTomato$^+$ (bottom) c-kit enriched BM, which were then cultured in vehicle or FLU then used in the transplantation experiments shown in Fig 1D.

J Representative analysis of donor HSCs (CD27$^+$ Ter119$^-$, c-kit$^+$, Sca-1$^+$, CD150$^+$, CD48$^-$) from competitive transplanted recipient BM 12 weeks post-transplant. Recipient from "Vehicle" control group is shown.

K Fold change in blood CD45 chimerism 12 weeks post-transplantation normalized to Vehicle group ($n = 12$, representative of nine independent experiments). Open symbols are females.

Data information: $*P \leq 0.05$; $**P \leq 0.01$; $***P \leq 0.001$ (Student's unpaired t-test). $M \pm$ SD shown. HSC, Hematopoietic stem cell, KLS, c-kit$^+$ lineage$^-$ sca-1$^+$ cell population, Dex, Dexamethasone, Flo., FLU, Veh., Vehicle, FMO, fluorescence minus one.

 © 2023 The Authors

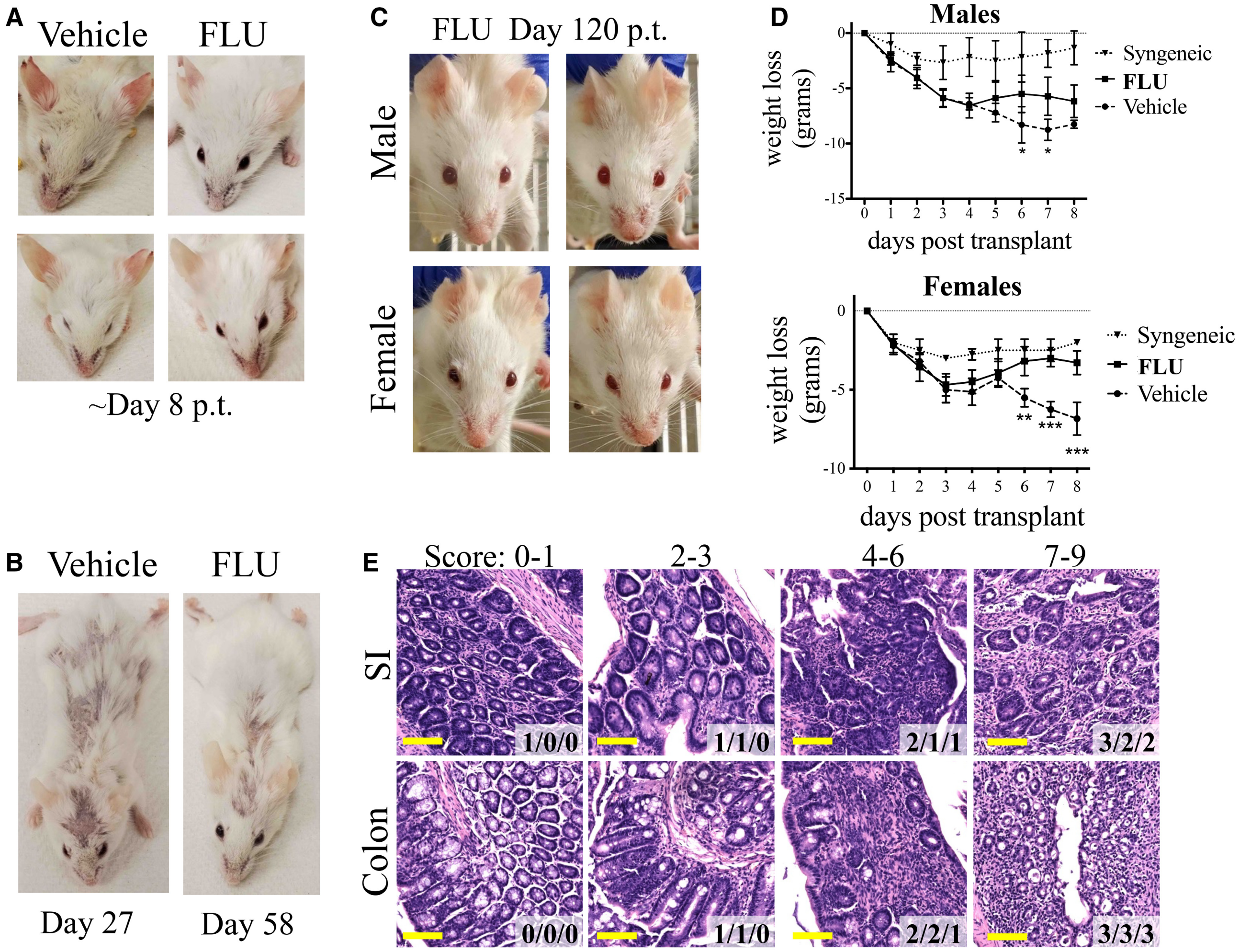

Figure EV2. Mice that received FLU-treated cells show less symptoms of GvHD compared to mice that received Vehicle-treated cells.

A Additional representative photographs of FLU and Vehicle treated cell recipients after approximately 7 days. Mice that were photographed were randomly chosen. Photos are taken of males except for the bottom left photograph is a female.

B Images of euthanized mice in each group at a later stage with skin lesions. Both are males.

C Images from remaining live healthy mice from the FLU-treated transplantation group approximately 120 days post-transplantation.

D Weight loss analysis of allogeneic transplants “FLU” and “Vehicle”, and “Syngeneic” vehicle (C57BL/6 into C57BL/6) transplanted mice separating males (top; n = 7 for “Vehicle”, n = 8 for “FLU”, n = 3 for “Syngeneic” groups) from females (bottom; n = 4 for “Vehicle”, n = 8 for “FLU”, n = 2 for “Syngeneic” groups). Significance is Vehicle group comparison to FLU group. Males at day 8 only had an n of 2.

E Comparison of small intestines (SI, top row), and colon (bottom row) of H&E stained sections representing different histopathological clinical scores. Tissues were scored by a blinded pathologist and assigned scores from 0 to 3 for three clinical signs of GvHD histopathology: lamina propria inflammation, crypt atrophy, and crypt epithelial apoptosis. Images represent different combined scores from 0 to 1 (leftmost), 2 to 3 (middle left), 4 to 6 (middle right), and 7 to 9 (rightmost). The three scores for each tissue is displayed in the lower right corner. Note that images represent only one region of the tissue, and tissues with lower scores had more regions of no pathology than those with higher scores. Yellow scale bars equal 100 microns.

Data information: *$P \leq 0.05$; **$P \leq 0.01$; ***$P \leq 0.001$; Student’s unpaired t-test. $M \pm$ SD shown. p.t., post-transplant.

© 2023 The Authors

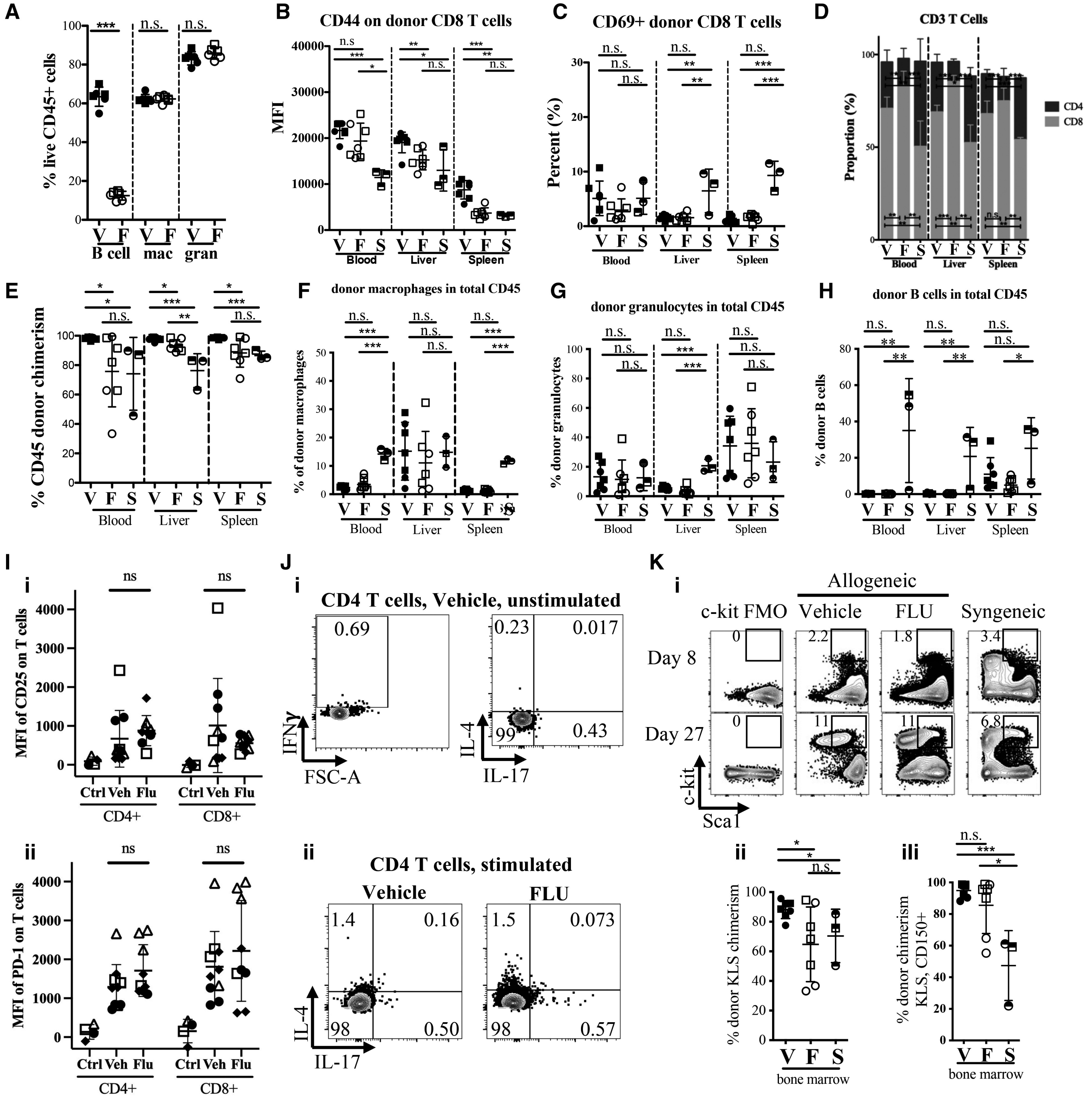

Figure EV3.

 © 2023 The Authors

Figure EV3. Additional characterization of donor cells in allogeneic transplanted recipients receiving either FLU or Vehicle-treated whole splenocytes and bone marrow cells.

A Quantification of live B cells (CD45$^+$, CD11b$^-$, Gr1$^-$, CD3$^-$, B220$^+$), macrophages (mac; CD45$^+$, Gr1$^-$, CD11b$^+$), and granulocytes (gran; CD45$^+$, CD11b$^+$, Gr-1$^+$) after 16 h culture with FLU or Vehicle (n = 6, representative of three independent experiments). FLU dramatically decreases B cell viability but does not affect myeloid populations.

B, C Quantification of percent CD69$^+$ cells (B) and MFI of CD44 expression (C) in donor CD8 population. Gated on live, CD45$^+$, CFP$^+$, CD11b$^-$, Gr1$^-$, B220$^-$, CD3$^+$, CD4$^-$, CD8$^+$ single cells. No significant differences were observed in CD69 or CD44 expression in CD8 T cells.

D Proportion (%) of CD4 (purple) and CD8 (gray) T cells within the CD3$^+$ T cell population. Overall, FLU-treated donors cells had a decrease in CD4 T cells compared to Vehicle-treated donor cells, though both FLU and Vehicle groups had a lower CD4:CD8 ratio than the syngeneic recipients.

E Total donor CD45$^+$ chimerism in blood, liver and spleen of transplanted recipients. Data is donor (CFP$^+$) CD45$^+$ cells as a percentage of total CD45$^+$ cells. At 1 week post-transplant, donor chimerism is slightly lower in the FLU group compared to vehicle, though this could be due to the lower number of viable cells transplanted after FLU culture.

F–H Quantification of percent donor macrophages (F) granulocytes (G) and B cells (H) in total CD45 cells. No significant differences between FLU and Vehicle groups were observed.

I Quantification of surface CD25 (i) and PD1 (ii) MFI expression in donor-derived (CFP$^+$) CD4 and CD8 T cells, 7–8 days after transplantation. Different symbols represent four independent experiments, open symbols are females, closed symbols are male, Ctrl are fresh B6-CFP spleen cells. While CD25 and PD1 are both upregulated in donor-derived cells 7–8 days after allogeneic transplantation, there is no significant difference between Vehicle and FLU-treated grafts.

J (i) IFNγ, IL-4, and IL-17 expression levels in unstimulated CD4 T cells from Vehicle recipient. These plots were used as a negative control for gating for the data in Fig 3E. (ii) Representative FACS plot of IL-4 and IL-17 expression of stimulated CD4 T cells, quantified in Fig 3Eiii. Please note that the data represented in Figs 3E and EV3I and J are from the same set of experiments.

K (i) Representative plot of donor (CFP$^+$) KLS population in BM from transplanted recipients of Vehicle-treated, FLU-treated, and Vehicle-treated-syngeneic donor cells from mice euthanized on day 8 p.t. (top) and on day 27 p.t (bottom). Donor KLS (ii) and KLS, CD150$^+$ (iii) chimerism in recipient BM after transplant. Data indicate a slight reduction in engraftment of FLU-treated BM compared to Vehicle-treated, but not in the CD150$^+$ KLS population, which is more highly enriched for HSCs. B-H: n = 7 for FLU and Vehicle groups days 6–9 p.t., representative of three independent experiments, n = 3 for Syngeneic Vehicle-treated (S) CFP$^+$ C57BL/6 into C57BL/6 group day 9 (1 mouse) and day 27 (2 mice) p.t., representative of two independent experiments. Circles represent males and squares represent females.

Data information: *$P \le 0.05$; **$P \le 0.01$; ***$P \le 0.001$ (Student's unpaired t-test). Error bars are SD. p.t., post-transplant, KLS, c-kit$^+$ lineage$^-$ Sca1$^+$ cell population, FMO, fluorescence minus one, V, Vehicle, F, FLU, S, syngeneic, MFI, mean fluorescent intensity, n.s. not significant.

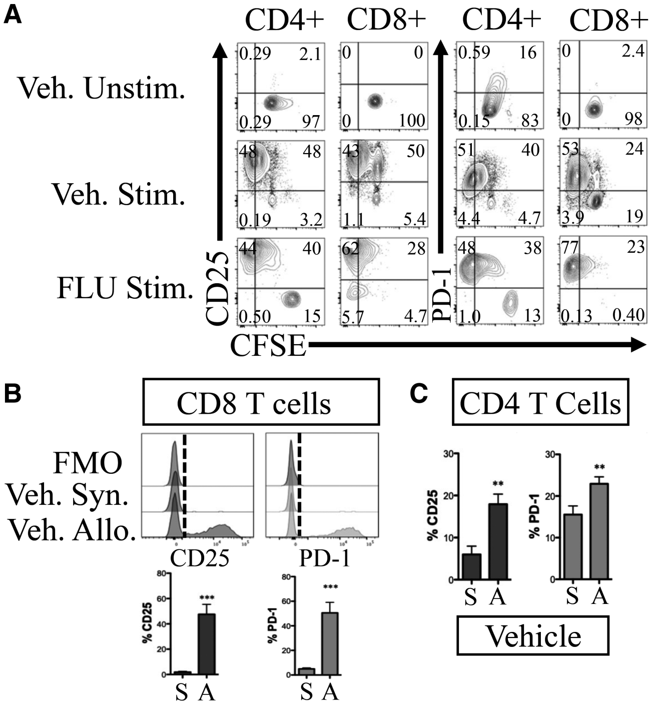

Figure EV4. Additional analysis of FLU-treated splenocytes after CD3/CD28 stimulation and MLR.

A Representative plots of proliferation (CFSE staining) vs activation markers CD25 and PD-1 expression on either FLU or Vehicle pretreated CD4$^+$ and CD8$^+$ cells after *in vitro* T cell activation assay via anti-CD3/CD28 antibody stimulation, from Fig 4A. Cells were stained with CFSE after FLU/vehicle culture and prior to anti-CD3/CD28 stimulation. In both vehicle and FLU conditions, stimulated cells diluted CFSE and upregulated CD25 and PD-1, indicating that anti-CD3/CD28 stimulation could rescue FLU-treated T cells from apoptosis and induce activation and proliferation.

B, C Representative histograms and quantification from MLR of CD25 and PD-1 expression on CD8 T cells (B) and CD4 T cells (C), from Fig 4B. Plots are gated on live, single, CD3$^+$ cells and either CD4$^+$ or CD8$^+$. Bar graphs display the percentages of CD25$^+$ or PD-1$^+$ T cells.

**$P \le 0.01$; ***$P \le 0.001$ (Student's unpaired t-test). Syn. or S., Syngeneic, Allo. or A., Allogeneic, Veh., Vehicle, Unstim., unstimulated, Stim., stimulated, Flo, FLU, FMO, fluorescence minus one.

© 2023 The Authors

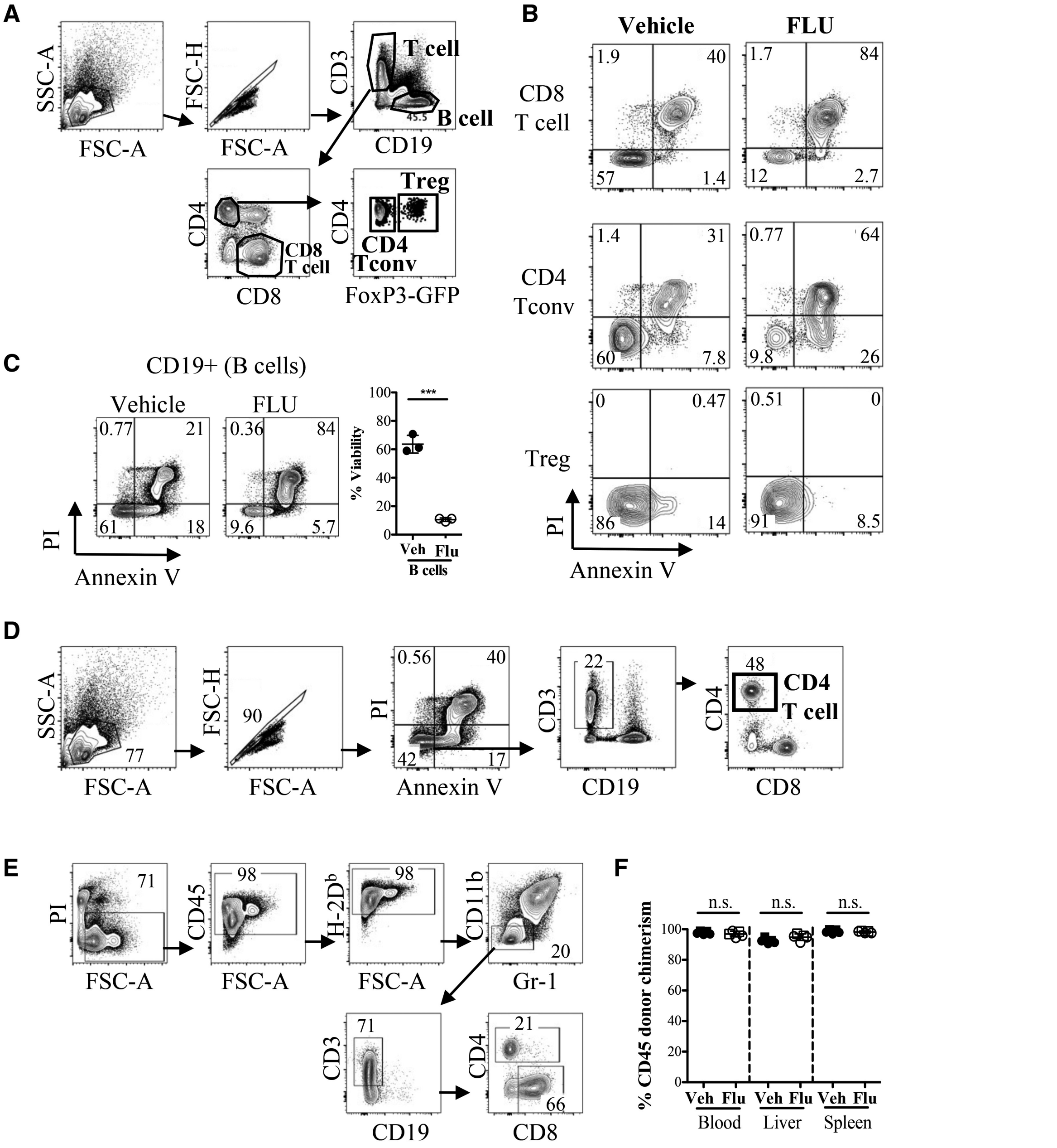

Figure EV5. Additional characterization of Tregs and other lymphocytes after FLU treatment *in vitro* and after allogeneic transplantation.

A Representative FACS gating scheme of FoxP3-GFP splenocytes after 16 h of treatment with FLU (3 nM) or vehicle (DMSO) before gating on viability using Propidium Iodide (PI) and apoptotic marker Annexin V, used in Fig 6A. Only vehicle treated cells are shown.

B Representative FACS plots showing live (PI^-, Annexin V^-), apoptotic (PI^-, Annexin V^+), and dead (PI^+, Annexin V^+) CD8 T cells, CD4 Tconv, and Tregs after 16 h FLU or vehicle culture. Data used for quantification in Fig 6A.

C Representative FACS plots (left) and quantitative data (right) showing B cell ($CD19^+$) viability ($n = 3$). B cell viability is poor after FLU treatment, with similar viability to that of CD4 Tconv and CD8 T cells.

D Representative FACS gating scheme of splenocytes after 16 h treatment with FLU or vehicle after gating on live (PI^-, Annexin V^-) cells. Data used for quantification in Fig 6B. Only vehicle treated cells shown. The same fcs files were used in EV5A.

E Representative FACS gating scheme of lineage cells from allogeneic recipient Balb/c (H-2^d) mice (spleen shown) 9 days after transplantation with donor FoxP3-GFP (H-2^b) mouse splenocytes and bone marrow. Data used for quantification in Fig 6C.

F Percent CD45 donor FoxP3-GFP (H-2^b) chimerism ($CD45^+$ H-$2D^{b+}$) as a percentage of total $CD45^+$ cells in recipient Balb/c (H-2^d) blood, liver and spleen 9 days post-transplantation ($n = 5$). Near 100% donor chimerism indicates a successful engraftment and hematopoietic reconstitution. n.s., not significant (Student's unpaired *t*-test).

 © 2023 The Authors
