# Supplementary material for: Graft conditioning with fluticasone propionate reduces graft‐versus‐host disease upon allogeneic hematopoietic cell transplantation in mice
Source: EMBO Mol Med. 2023 Aug 4;15(9):e17748. doi: 10.15252/emmm.202317748 (PMC10493574; doi:10.15252/emmm.202317748)
Supplement: Supplementary file 3 — PDF+ [file EMMM-15-e17748-s009.pdf]

Article

SOURCE DATA | TRANSPARENT PROCESS | OPEN ACCESS

EMBO Molecular Medicine

Graft conditioning with fluticasone propionate reduces graft-versus-host disease upon allogeneic hematopoietic cell transplantation in mice

Erika S Varady[1,2,†], L Angel Ayala[1,2,†], Pauline U Nguyen[1,2], Vanessa M Scarfone[1], Alborz Karimzadeh[1,2,‡], Cuiwen Zhou[1,2], Xiyu Chen[1,2], Scott A Greilach[1,2], Craig M Walsh[1,2] & Matthew A Inlay[1,2,*]

Abstract

Hematopoietic cell transplantation (HCT) treats many blood conditions but remains underused due to complications such as graft-versus-host disease (GvHD). In GvHD, donor immune cells attack the patient, requiring powerful immunosuppressive drugs like glucocorticoids (GCs) to prevent death. In this study, we tested the hypothesis that donor cell conditioning with the glucocorticoid fluticasone propionate (FLU) prior to transplantation could increase hematopoietic stem cell (HSC) engraftment and reduce GvHD. Murine HSCs treated with FLU had increased HSC engraftment and reduced severity and incidence of GvHD after transplantation into allogeneic hosts. While most T cells died upon FLU treatment, donor T cells repopulated in the hosts and appeared less inflammatory and alloreactive. Regulatory T cells (Tregs) are immunomodulatory and survived FLU treatment, resulting in an increased ratio of Tregs to conventional T cells. Our results implicate an important role for Tregs in maintaining allogeneic tolerance in FLU-treated grafts and suggest a therapeutic strategy of pre-treating donor cells (and not the patients directly) with GCs to simultaneously enhance engraftment and reduce GvHD upon allogeneic HCT.

Keywords allogeneic transplantation; blood and marrow transplantation; glucocorticoids; graft-versus-host disease; hematopoietic stem cells

Subject Categories Haematology; Immunology; Stem Cells & Regenerative Medicine

DOI 10.15252/emmm.202317748 | Received 23 March 2023 | Revised 30 June 2023 | Accepted 10 July 2023 | Published online 4 August 2023

EMBO Mol Med (2023) 15: e17748

Introduction

Hematopoietic cell transplantation (HCT), also known as blood and marrow transplantation (BMT), is a potentially curative treatment for many hematologic malignancies (Bair *et al*, 2020). Hematopoietic stem cells (HSCs) are the key component to HCT due to their ability to self-renew, differentiate into lineage blood cells, and engraft the bone marrow from the blood (Hendrikx *et al*, 1996; Iscove & Nawa, 1997; van Os *et al*, 2010; Ratajczak & Suszynska, 2016). A low number of transplanted HSCs can result in disease relapse and opportunistic infections after HCT; therefore, increasing engraftment efficiency and the number of engrafted HSCs would aid a successful HCT (Gluckman *et al*, 1997; Bahçeci *et al*, 2000). C-X-C chemotactic receptor type 4 (CXCR4) is a chemokine receptor important for HSC homing to the bone marrow and subsequent engraftment through response to a blood chemotactic gradient of stromal derived factor 1α (SDF-1α, CXCL12) secreted by bone marrow stromal cells (Peled *et al*, 1999; Sharma *et al*, 2011). Transgenic upregulation of CXCR4 expression on human HSCs has been shown to increase bone marrow engraftment upon transplantation in immunocompromised mice (Brenner *et al*, 2004). Furthermore, Guo *et al* (2017) found that treating human cord blood HSCs with the glucocorticoid (GC) fluticasone propionate (FLU), also known as Flonase, could increase surface CXCR4 expression, leading to increases in transwell migration of human cord blood HSCs toward an SDF-1α gradient, as well as increasing bone marrow engraftment in immunodeficient mice.

Apart from disease relapse, a major complication causing high morbidity and mortality following allogeneic HCT (allo-HCT) is acute and/or chronic Graft-versus-Host Disease (GvHD). GvHD occurs when donor immune cells, or graft cells, recognize the host as a foreign threat, and initiate an immune response against the host, causing severe tissue damage, organ failure, and possibly death. Up to 40–60% of allo-HCT patients develop acute GvHD due to a human leukocyte antigen (HLA) mismatch between donor and recipient (Jagasia *et al*, 2012). In a mismatched setting, as many as 10% of donor T cells may respond allogeneically to host cells, and systemically infiltrate and damage healthy recipient tissues (Ashwell *et al*, 1986; Benichou *et al*, 1999; Suchin *et al*, 2001). Strategies to

1 Sue and Bill Gross Stem Cell Research Center, University of California Irvine, Irvine, CA, USA
2 Department of Molecular Biology and Biochemistry, University of California Irvine, Irvine, CA, USA
*Corresponding author. Tel: +1 949 824 8226; Fax: +1 949 824 1272; E-mail: minlay@uci.edu
†These authors contributed equally to this work
‡Present address: Joslin Diabetes Center, Harvard Medical School, Boston, MA, USA

© 2023 The Authors. Published under the terms of the CC BY 4.0 license

prevent GvHD focus on T cell suppression or depletion (El-Jawahri *et al*, 2016; Khoury *et al*, 2017). Calcineurin inhibitors (tacrolimus or cyclosporine) combined with methotrexate can suppress T cell activation and proliferation (Storb *et al*, 1986; Ruutu *et al*, 2014; Cronstein & Aune, 2020). Antibody-mediated T cell depletion *ex vivo* or *in vivo* can prevent GvHD, yet also can result in disease relapse due to graft failure and a reduction of the Graft versus Leukemia (GvL) effect (Ho & Soiffer, 2001). GvL is an important benefit to allo-HCT, where donor T cells eliminate patient tumor cells. Patients that develop GvHD symptoms can be treated with glucocorticoids (GCs) such as methylprednisone, which are powerful immunosuppressants but have harmful side effects when administered globally and in prolonged treatment courses (Fuji *et al*, 2021). Lastly, treatment with high dose cyclophosphamide (PTCy) *after* haplo-identical transplantation targets activated allogeneic T cells, and can prevent GvHD while maintaining GvL (Luznik *et al*, 2001, 2008), but has shown mixed results and the risk of GvHD remains (Modi *et al*, 2020; Irene *et al*, 2021).

Regulatory T cells (Tregs) are known to reduce GvHD severity by suppressing alloreactive T cell function and creating immune tolerance after allo-HCT (Hoffmann *et al*, 2002; Di Ianni *et al*, 2011). In GvHD, there are reduced levels of Tregs and higher levels of $CD4^+$ conventional T cells (Tconv), which can be alloreactive (Edinger *et al*, 2003; Zorn *et al*, 2005). Transplanting *in vitro* expanded Tregs at the same time as allo-HCT prevents acute GvHD (aGvHD) (Riegel *et al*, 2020). Treg therapy is an important area of research for creating immune tolerance in allogeneic donor cells to alleviate GvHD, and clinical trials infusing either primary or *in vitro* expanded Tregs have shown some success at reducing GvHD (Brunstein *et al*, 2011; Di Ianni *et al*, 2011; Martelli *et al*, 2014; MacMillan *et al*, 2021). However, there is a delicate balance of preventing GvHD while keeping the beneficial GvL response (Riegel *et al*, 2020). Many groups aim to decrease immune dysregulation in GvHD either by depleting alloreactive T cells or increasing the number of Tregs in the graft.

In this study, we tested the hypothesis that conditioning donor cells by pre-treatment with the glucocorticoid FLU *prior to* allo-HCT could both enhance HSC engraftment and also suppress allogeneic T cell activation to prevent or reduce GvHD.

Results

FLU increases HSC migration and engraftment by upregulating CXCR4

In the study by Guo *et al* (2017), the authors found improvements in human cord blood HSC CXCR4 expression, migration and engraftment following pre-treatment with FLU, but did not observe a similar effect on murine HSCs. We re-examined the impact of GCs on murine HSCs (Figs 1 and EV1). We first cultured c-kit enriched murine BM with different concentrations of FLU or Dexamethasone (Dex) for 16 h, then examined CXCR4 expression and viability on phenotypic HSCs ($Ter119^-$ $CD27^+$ $ckit^+$ $Sca1^+$ $CD150^+$ $CD34^-$; Fig EV1A and B). We found that 3 nM FLU was the best concentration and glucocorticoid that led to a significant upregulation of CXCR4 expression on HSCs and the overall KLS ($ckit^+$ Lin^- $Sca1^+$) population with no detrimental effect on HSC viability (Figs 1A and EV1C and 1D). We also examined different formulations of HSC cell culture media and found in all cases that FLU increased CXCR4 expression on HSCs (Fig EV1E). We blocked CXCR4 upregulation with the glucocorticoid receptor (GR) antagonist RU486, demonstrating that FLU acted through the GR (Fig 1B). FLU induced CXCR4 expression on HSCs, but did not increase the expression level of CXCR4 on HSCs that were already $CXCR4^+$ (Fig EV1F and G).

We next tested whether FLU could increase migration of HSCs toward SDF-1α (CXCL12), the ligand for CXCR4. A transwell migration assay using FLU pre-treated c-kit-enriched BM showed a significant increase in the percentage of HSC migration toward SDF-1α relative to vehicle-treated HSCs (Figs 1C and EV1H). In addition, when the CXCR4 antagonist AMD3100 was added to the FLU culture, migration was blocked, demonstrating that FLU improved HSC migration through the CXCR4 pathway.

To determine whether FLU treatment led to improvements in HSC engraftment *in vivo*, we performed syngeneic competitive transplantations with HSCs pre-treated with FLU or the vehicle DMSO (Figs 1D and EV1I–K). In these experiments, sorted KLS ($ckit^+Lin^-Sca1^+$) cells from CFP^+ mice were treated with either FLU or vehicle, then transplanted into separate lethally-irradiated Wt B6 recipients. In addition, vehicle-treated KLS cells from $tdTomato^+$ mice were co-transplanted as a competitor in a 1:1 ratio with the CFP^+ cells. At 12 weeks post-transplantation, HSC chimerism was compared between FLU and vehicle donors (CFP) after normalizing to their competitor internal controls ($tdTomato^+$ HSCs, Figs 1Di and EV1I). Although there was no significant difference in total blood CD45 chimerism, there was a mild but significant increase in bone marrow HSC engraftment from FLU pre-treated HSCs 3 months after transplantation (Figs 1Dii and EV1J and K). Overall, we have shown that FLU increases CXCR4 expression on murine HSCs and improves murine HSC migration and bone marrow engraftment after transplantation.

Pre-treating donor cells with FLU decreases aGvHD in mice

Given the immunosuppressive ability of glucocorticoids, we reasoned that a pre-treatment of donor cells with FLU prior to transplantation would not only enhance HSC engraftment, but potentially suppress an allogeneic immune cell response and thereby reduce or prevent GvHD. To address this, we used an aGvHD mouse model whereby B6 BM and spleen cells ($H\text{-}2^b$ MHC background) are transplanted into Balb/c recipients ($H\text{-}2^d$ background). In this setting, recipients develop severe aGvHD in 7–10 days (Figs 2 and EV2; van Leeuwen *et al*, 2002). We pre-treated fresh CFP^+ (B6 background) whole bone marrow cells (2–3 × 10^6 cells) and splenocytes (6 × 10^6 cells) with 3 nM FLU for 16 h and then transplanted them into lethally-irradiated (850 cGy) Balb/c mice (Fig 2Ai). A syngeneic control group used B6 recipients (Fig 2Aii). Recipient mice were scored for GvHD severity based on Naserian *et al* (2018) assigning 1 point for each GvHD symptom including hunched back, skin lesions, dull fur, diarrhea, and 10% loss of initial weight.

After unblinding the study, we observed a phenotypic difference between recipients that received FLU- or Vehicle-treated donor cells (Figs 2B and EV2A and B). "Vehicle" mice displayed discharge around their eyes with dull fur around the nose around 1-week post-transplant (p.t.) and all but one were euthanized within 9 days p.t. due to a weight loss of ≥ 30%, with the last euthanized at day

 © 2023 The Authors

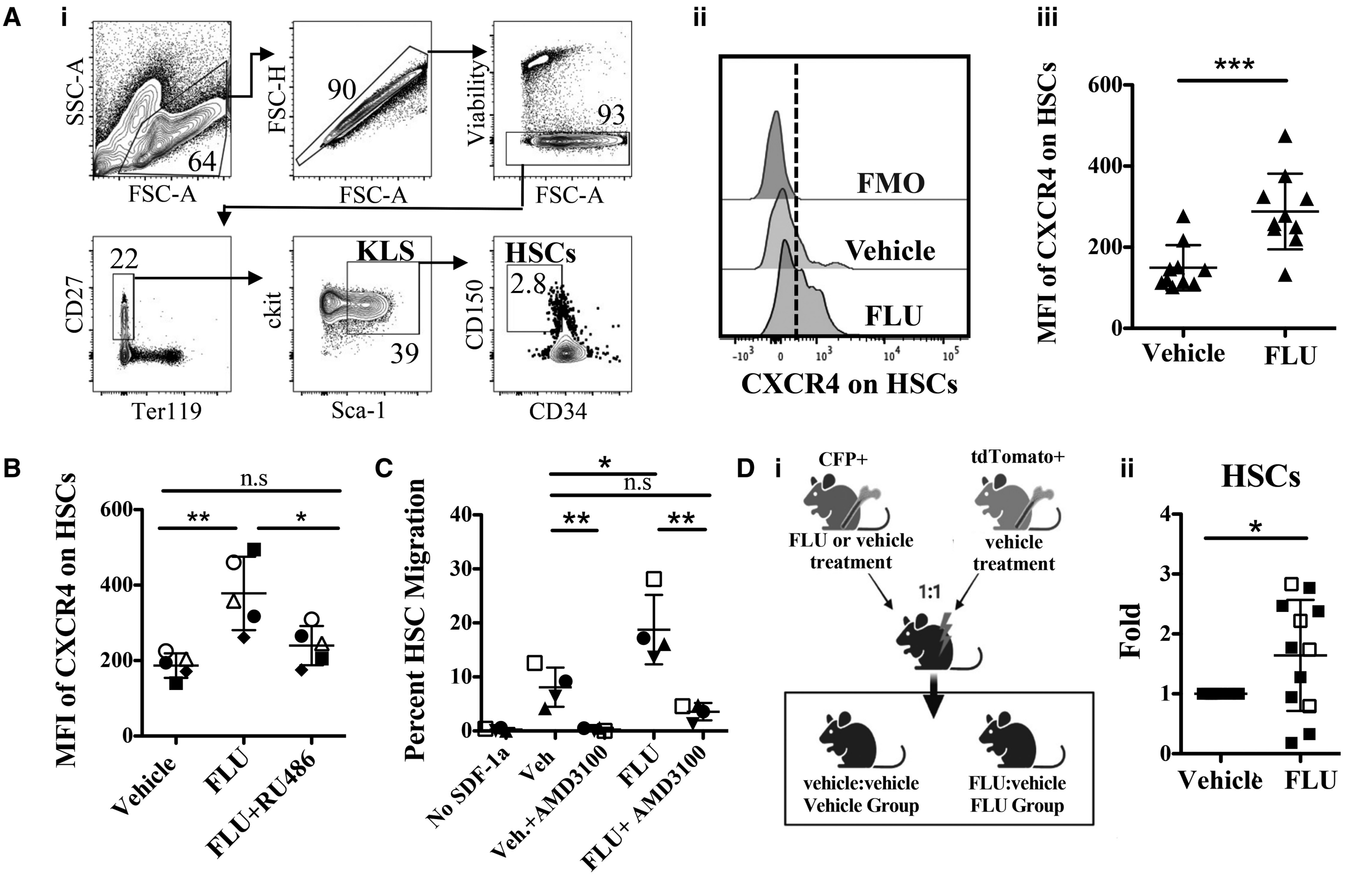

Figure 1. Effects of FLU on murine HSC surface expression and function.

A (i) Representative gating strategy for KLS population (CD27$^+$ Ter119$^-$ ckit$^+$ Sca1$^+$) and HSCs (CD27$^+$ Ter119$^-$ ckit$^+$ Sca1$^+$ SlamF1$^+$ CD34$^-$) in mouse BM. (ii) Representative histograms of surface CXCR4 expression on HSCs after 16 h of culture in Vehicle (DMSO, red histogram) or 3 nM FLU (blue histogram). The dashed line indicates cutoff for positive CXCR4 expression based on the fluorescence minus one (FMO, gray histogram) control. (iii) Flow cytometry quantification of median fluorescent intensity (MFI) of surface CXCR4 expression on HSCs (n = 10, representative of seven independent experiments). FLU concentration is 3 nM unless otherwise indicated.

B MFI of surface CXCR4 on HSCs after Vehicle, FLU or FLU $^+$ RU486 (GR antagonist) treatment (n = 5, three independent experiments shown; each symbol represents a mouse throughout each condition, open symbols are females and closed symbols are males).

C Transwell migration of HSCs toward SDF-1α (50 ng/ml) after 16 h pre-treatment in Vehicle or FLU then a 30-min incubation with or without CXCR4 antagonist (AMD3100; n = 4, four independent experiments shown; each symbol represents a mouse throughout each condition, the open symbol is a female and closed symbols are males).

D (i) Scheme of competitive syngeneic transplant of FACS sorted BM KLS cells from fluorescently labeled CFP$^+$ or tdTomato$^+$ mice co-transplanted in equal amounts into lethally-irradiated (850 cGy) C57BL/6 recipients. CFP$^+$ cells were pre-treated in vehicle or FLU, while tdTomato$^+$ cells were only pre-treated in vehicle prior to transplantation. (ii) Flow cytometry analysis of donor HSC chimerism in the BM 12 weeks post-transplant in both recipient groups. Shown is the percentage of CFP$^+$ HSCs in the "FLU Group" relative to the percentage of CFP$^+$ HSCs in the "Vehicle Group" (Vehicle; n = 12, representative of nine independent experiments). Open symbols are females, closed are males.

Data information: *$P \le 0.05$; **$P \le 0.01$; ***$P \le 0.001$ (Student's unpaired t-test (1A, 1B, 1C), One sample t-test (1D)). $M \pm$ SD shown. BM, bone marrow, HSC, Hematopoietic stem cell, KLS, kit$^+$ lineage$^-$ Sca1$^+$ cell population, GR, glucocorticoid receptor, Veh., Vehicle, FMO, fluorescence minus one.
Source data are available online for this figure.

27 (Fig 2B and C). On the other hand, only half of the "FLU" recipients died due to GvHD and the other half survived with only minimal GvHD symptoms (clinical score < 2, Figs 2C and EV2C). FLU recipients also had a significantly lower overall clinical score compared to the vehicle group (Fig 2D), with no gender difference observed (Fig EV2D). As expected, the "Syngeneic" recipients all recovered quickly with mild symptoms (Fig 2C and D).

Tissues (small intestines (SI) and colon) were selected from each group, fixed, sectioned, H&E stained, then scored by a blinded pathologist (Figs 2E and F, and EV2E). Both SI and colon sections from the vehicle group displayed visible signs of severe GvHD pathology, including inflammation of the lamina propria (LP), crypt epithelial atrophy, and apoptotic crypt epithelial cells. Sections from the FLU group displayed less severe histopathology, with scattered LP inflammation and crypt atrophy interspersed with regions of no pathological alteration (NPA). While there appeared to be gross differences between vehicle and FLU tissues, there were no statistically significant differences between the

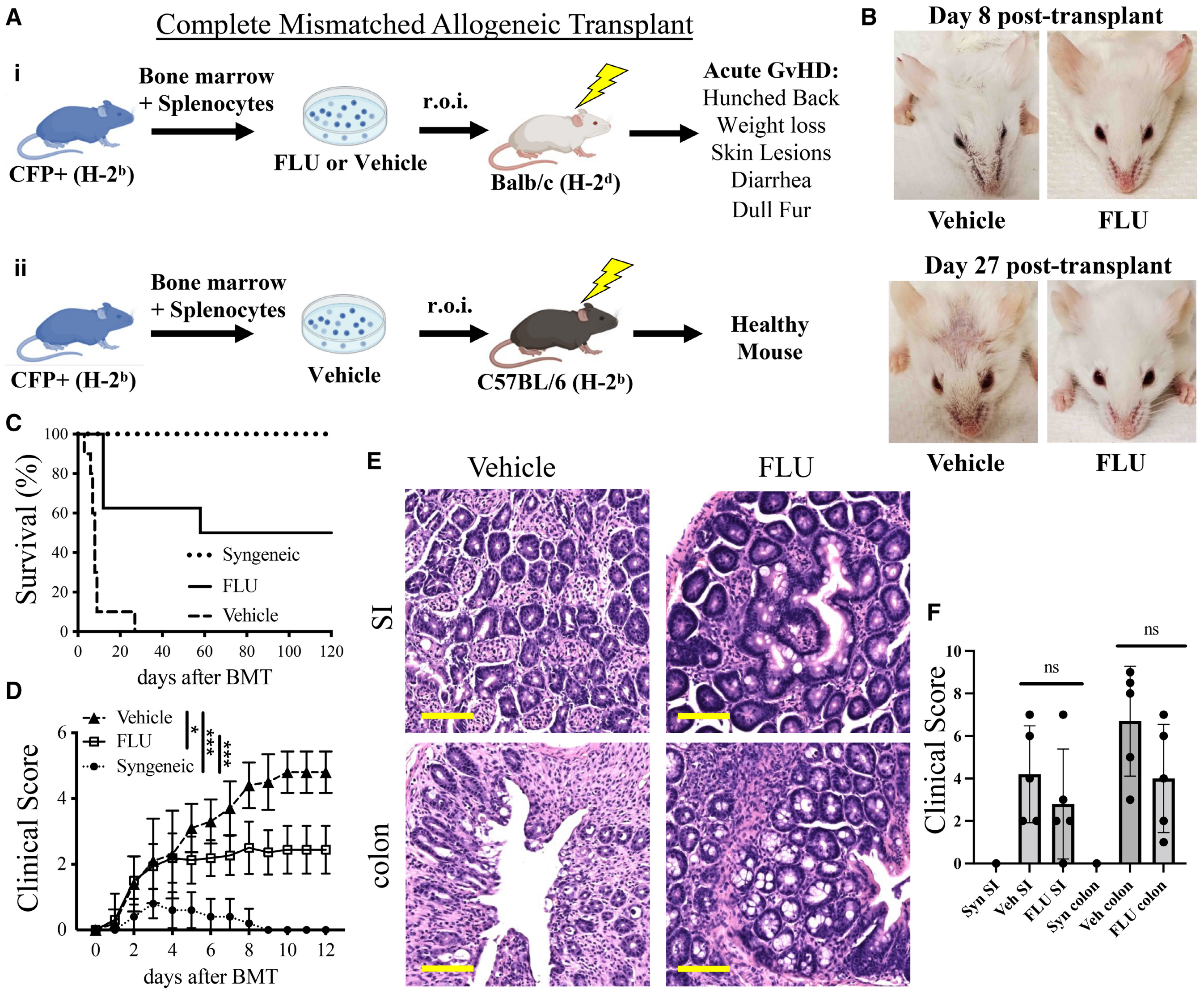

Figure 2. GvHD analysis after allogeneic transplantation of FLU pre-treated splenocytes and bone marrow cells in mice.

A (i) Scheme of allogeneic transplant of 6×10^6 whole splenocytes plus 3×10^6 whole bone marrow cells from CFP^+ (H-2^b) mice after FLU or vehicle culture into lethally-irradiated (lightning symbol) Balb/c recipients. (ii) Scheme of Syngeneic transplant, which is identical to the allogeneic transplant but with C57BL/6 (H-2^b) recipients.

B Representative images of allogeneic Balb/c (H-2^d) recipients at 8 days (top) and 27 days (bottom) post-transplantation of FLU (left) or vehicle (right) treated cells.

C GvHD survival curve of “FLU” or “Vehicle” allogeneic recipients or “Syngeneic” recipients through 120 days post-transplantation. Mice were euthanized at ≥ 30% loss of initial weight or after receiving a clinical score of 5. n = 10 for “Vehicle” group, n = 8 for “FLU” group, n = 4 for “Syngeneic” until day 27 then n = 2 thereafter not due to GvHD, but data collection for Fig 3; with half females and half males for all groups; representative of three independent experiments.

D Quantification of clinical scoring system of GvHD in recipients. One point was assigned for each symptom of GvHD: hunched back, dull fur, skin lesion, diarrhea, and 10% loss of initial weight. An automatic full score of 5 was assigned to recipients that experienced ≥ 30% loss of initial weight (n = 10 for Vehicle group and n = 16 for FLU group with half females and half males for both groups; representative of three independent experiments).

E Representative H&E stained sections of the small intestines (SI) and colon. Signs of pathology included inflammation of the lamina propria, crypt atrophy, and apoptotic crypt epithelial cells.

F Clinical scores of the SI and colon for Syngeneic, Vehicle, and FLU groups. Sections were scored as NPA (no pathological alteration), minor, moderate, or severe GvHD pathology by a blinded histologist, and scored from 0 to 3, respectively, for each tissue, for each clinical sign as mentioned above.

Data information: Scale bars (yellow) equal 100 microns. $*P \leq 0.05$; $***P \leq 0.001$ (Student's unpaired t-test). $M \pm$ SD shown. GvHD, graft-vs-host disease, r.o.i, retro-orbital injection, CFP, cyan fluorescent protein, SI, small intestines, H&E, hematoxylin and eosin, n.s., not significant.
Source data are available online for this figure.

 © 2023 The Authors

clinical scores in either the SI or colon between five sets of tissues (Fig 2F).

FLU-pretreated donor T cells engraft and expand, but do not activate in allogeneic recipients

Glucocorticoids are known to cause apoptosis in lymphocytes, and we found that after 16 h in culture, there was a significant reduction of T and B lymphocyte viability in the FLU-treated group compared to the vehicle control, with little effect on macrophages or granulocytes (Figs 3A and EV3A). This suggested that the reduction in GvHD symptoms in the "FLU" mice could be due to the absence of viable T cells in the graft. However, after approximately 1-week post-transplantation, we saw a robust engraftment of donor T cells ($CD3^{+}$) in the "FLU" recipients' blood, spleen, and liver, similar to the "Vehicle" recipients in the spleen and liver, albeit slightly lower in the blood (Fig 3B and C). This expansion of donor T cells was not observed in the "Syngeneic" recipients, indicating T cell expansion only in the allogeneic setting. Thus, the absence of GvHD in the "FLU" recipients is likely not due to a lack of donor T cell engraftment.

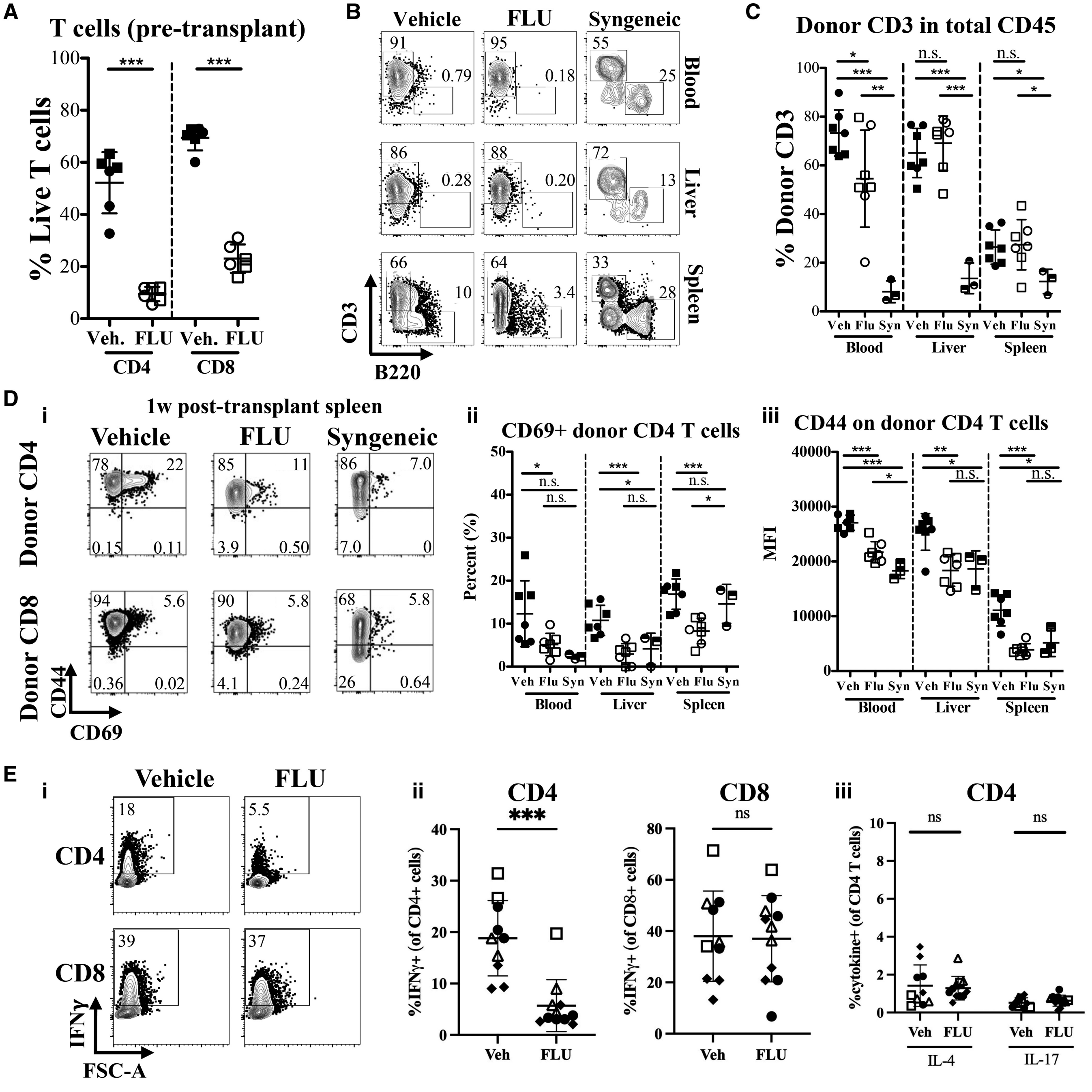

Figure 3.

Figure 3. Characterization of donor T cells in allogeneic transplanted recipients receiving either FLU or vehicle-treated whole splenocytes and bone marrow cells.

Data from allogeneic transplant of whole splenocytes plus whole bone marrow cells from CFP^+ (H-2^b) mice after FLU or vehicle (DMSO) culture into lethally-irradiated Balb/c (H-2^d) recipients. Syngeneic (Syn) transplant controls are C57BL/6 recipients that received Vehicle treated CFP^+ cells.

- A Percent live (Annexin V^-, PI^-) $CD4^+$ T cells ($CD11b^-$, $Gr1^-$, $CD19^-$, $CD45^+$, CFP^+, $CD8^-$, $CD4^+$) and CD8 T cells ($CD11b^-$, $Gr1^-$, $CD19^-$, $CD45^+$, CFP^+, $CD4^-$, $CD8^+$) from spleen after 16 h culture with FLU or vehicle and *prior to* transplantation (n = 6, representative of three independent experiments). Circles represent males and squares represent females.
- B Representative FACS plots of donor (CFP^+) CD3 and B220 populations in blood, liver and spleen of recipient Balb/c mice 7–10 days after transplantation with Vehicle (left column), or FLU (middle column) treated cells, or Syngeneic hosts (right column). Gated on live, $CD45^+$, CFP^+, $CD11b^-$, $Gr1^-$ single cells.
- C Quantification of the percentage of donor $CD3^+$ T cells in total $CD45^+$ hematopoietic cells in blood, liver and spleen of recipient mice. Gated on live, $CD45^+$, CFP^+, $CD11b^-$, $Gr1^-$, $B220^-$ single cells. (n = 7 biological replicates for Vehicle and Flonase, n = 3 biological replicates for Syngeneic). Circles represent males and squares represent females.
- D (i) Representative plots showing activation markers CD44 and CD69 expression on donor CD4 (top row) and CD8 (bottom row) T cells from Vehicle, FLU and Syngeneic recipient groups. Gated on live, $CD45^+$, CFP^+, $CD11b^-$, $Gr1^-$, $B220^-$, $CD3^+$, single cells. Quantification of percent $CD69^+$ cells (ii) and MFI of CD44 (iii) in donor CD4 T cell population. Gated on live, $CD45^+$, CFP^+, $CD11b^-$, $Gr1^-$, $B220^-$, $CD3^+$, $CD8^-$, $CD4^+$ single cells. (n = 7 for FLU and Vehicle groups days 6–9 p.t., representative of three independent experiments; n = 3 for Syngeneic (Syn) group day 9 (1 mouse) and day 27 (2 mice) p.t., representative of two independent experiments). Circles represent males and squares represent females.
- E (i) Representative FACS plots showing intracellular IFNγ expression (y-axis) on total $CD4^+$ (top row) and $CD8^+$ (bottom row) T cells 1 week post-transplant from the spleens of mice that received vehicle (left) or FLU-treated (right) cells, 4 h after PMA/ionomycin stimulation. Gated on live, $CD45^+$, $CD3^+$, single cells. Gates are based on IFNγ staining of unstimulated cells (Fig EV3Ji). (ii) Quantification of IFNγ^+ T cells (CD4 left, CD8 right) from four independent experiments. (iii) Quantification of IL-4^+ (left) and IL-17^+ (right) CD4 T cells 4 h after PMA/ionomycin stimulation. Gating was based on unstimulated cells (Fig EV3Ji). Representative stain shown in Fig EV3Jii. n = 10 for vehicle, and n = 11 for Flonase, shapes represent different experiments, open symbols are female, closed symbols are male.

Data information: *$P \le 0.05$; **$P \le 0.01$; ***$P \le 0.001$ (Student's unpaired t-test). $M \pm$ SD shown. p.t., post-transplant, Veh, Vehicle, syn, syngeneic, ns not significant. Source data are available online for this figure.

Glucocorticoids are immunosuppressive and decrease many immune cell functions such as cytokine production, signal transduction, and TCR signaling. To assess a reduction in T cell activation in the "FLU" group, we analyzed leukocyte early activation markers CD69 and CD44 (Fig 3D). Both markers are elevated on infiltrating T cells in allogeneic transplantation in mice (Beilhack *et al*, 2005; Schumann *et al*, 2015). In the FLU group, we observed a significant decrease in the percentage of $CD69^+$ CD4 T cells, and reduced CD44 expression compared to the Vehicle group, in the blood, liver, and spleen, indicating a reduction in T cell activation in FLU-treated CD4 T cells (Fig 3D).

CD8 T cells appeared less activated, with little difference in CD69 positivity or CD44 expression between vehicle and FLU groups, although CD8 T cells are known to lose CD69 expression 6 days after allo-HCT (Fig EV3B–D; Beilhack *et al*, 2005). Donor B cell, macrophages, and granulocytes were also examined with no significant differences observed between the groups (Fig EV3E–H). We also examined expression of activation markers CD25 and PD-1, and while both markers were upregulated compared to fresh spleen controls, there was no significant difference between donor-derived T cells between the two groups (Fig EV3I).

Next, we sought to determine the ratios of T cell effector subsets in mice that received either vehicle or FLU-treated splenocytes (Figs 3E and EV3J). After 1 week post-transplant, we analyzed the spleen for Th1, Th2, and Th17 cells based on cytokine secretion (IFNγ, IL-4, and IL-17, respectively). We saw a significant reduction in Th1 effector cells (IFNγ^+) in mice that receive FLU-treated cells compared to the vehicle control (Fig 3Ei–ii). There was no difference in Th2 (IL-4^+) and Th17 (IL-17^+) subsets between the two groups (Figs 3Eiii and EV3J). This suggests that a reduction in Th1 response may contribute to reducing GvHD severity in our model. Conversely, CD8 T cells had a similar percentage of IFNγ expressing T cells between vehicle and FLU groups (Fig 3Eii).

Lastly, we examined overall donor chimerism (including stem/progenitors in the BM) and saw little difference in chimerism, indicating BM engraftment was robust in both groups (Fig EV3K). Together, these data provide some insight on a possible explanation for FLU-mediated reduction in aGvHD in mice involving a decrease in activation of CD4 T cells and reduced inflammatory Th1 response.

T cells treated with FLU activate and expand after anti-CD3/CD28 co-stimulation, but not when cultured with allogeneic splenocytes

To better understand how FLU inhibits T cell activation, we examined *in vitro* stimulation of the TCR through two classic assays: (i) using antibodies against CD3 and CD28, and (ii) using a mixed lymphocyte reaction (Figs 4 and EV4). In the first assay, B6 spleen cells were pre-treated for 16 h with Vehicle or FLU, then stimulated with anti-CD3/CD28 antibodies (Fig 4A). Stimulated cells increased in size (via forward scatter, FSC-A) indicating activation (Fig 4Ai), and diluted CFSE, indicating proliferation (Fig EV4A), in both vehicle and FLU cultures. Unstimulated FLU-treated T cells all died, as expected. Stimulated CD4 and CD8 cells in both conditions also displayed significant increases of the activation markers CD25 and PD-1 compared to the unstimulated Vehicle group (Figs 4Aii–iii and EV4A). These results suggest that activation of T cells via stimulation of the TCR (via CD3) and co-stimulatory molecule (CD28) allows T cells to survive and overcome FLU-induced apoptosis.

We next performed a mixed lymphocyte reaction (MLR) to see if FLU pre-treated T cells can be activated in an allogeneic manner *in vitro*. CFP^+ splenocytes (H-2^b) were cultured in FLU or vehicle for 16 h then mixed with syngeneic (B6) or allogenic (Balb/c, H-2^d) irradiated splenocytes (Figs 4B and EV4B and C). As expected, vehicle treated T cells expanded when mixed with allogeneic stimulator cells, upregulated activation markers CD25 and PD1, and diluted CFSE relative to the syngeneic condition, indicating allogeneic T cell activation (Figs 4Bi–ii and EV4B and C). In contrast, FLU pre-treated splenocytes did not survive, proliferate nor activate when cultured with allogeneic stimulator cells (Fig 4Bi). Because FLU-treated T cells can activate in response to CD3/CD28 signaling, but not when mixed with allogeneic stimulator cells, this indicates that allogeneic

 © 2023 The Authors

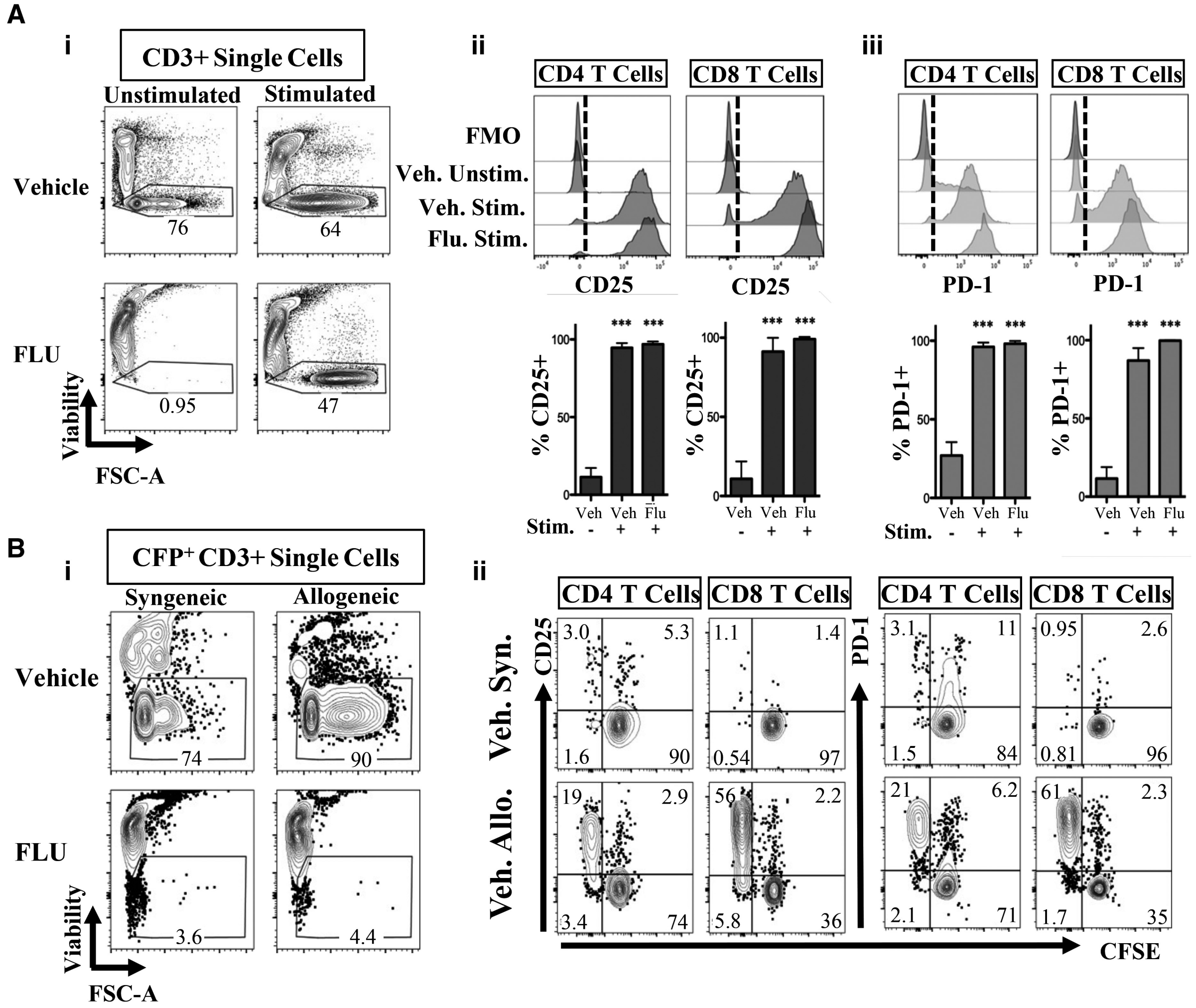

Figure 4. Effects of FLU on T cells activated *in vitro*.

A Analysis of T cell activation with anti-CD3/CD28 antibodies after pre-treatment in FLU or vehicle. 6×10^5 CFP+ splenocytes were pre-treated for 16 h in vehicle or FLU, then stimulated with anti-CD3 (plate-bound) and anti-CD28 (in solution) antibodies, or media only (unstimulated) for 4 days (n = 3; M ± SD; two male and one female, representative of two independent experiments). (i) Representative plots of viability (PI) vs size (FSC-A) gated on CD3+ single cells. (ii–iii) Histograms and quantification of activation markers CD25 (ii) and PD-1 (iii) expression on CD4 and CD8 T cells. Gated on live, single CD3+ cells, and either CD4+ or CD8+. The vertical dashed line represents the cutoff for positive expression, based on the FMO control which contained a pool of cells from all conditions. Bar graphs show the percentage of CD25+ or PD-1+ CD4 or CD8 T cells.

B Mixed lymphocyte reaction (MLR) analysis after 4 days in culture using a 1:1 ratio of 3×10^5 irradiated (2,500 cGy) stimulator cells, to 3×10^5 unirradiated responder cells (n = 3; M ± SD; two male and one female). Responder cells are CFP+ (H-2^b) and were first cultured for 16 h in FLU or vehicle, then stained with CFSE before the MLR assay. Stimulator cells are either Balb/c (H-2^d, Allogeneic) or C57BL/6 (H-2^b, Syngeneic). (i) Representative viability (PI) vs size (FSC-A) FACS plots of responder T cells (CFP+, CD3+). (ii) Representative plots of cell proliferation (CFSE staining) vs activation marker expression (CD25 and PD-1) on CD4 and CD8 T cells after stated culture conditions. Only vehicle treated cells are shown, due to poor viability of FLU-treated cells.

Data information: ***$P \leq 0.001$ (Student's unpaired t-test). Syn., Syngeneic, Allo., Allogeneic, Veh., Vehicle, FMO, fluorescence minus one.
Source data are available online for this figure.

stimulation is not sufficient to overcome the suppression caused by FLU treatment.

Donor T cells pre-treated with FLU were not alloreactive when transplanted into secondary allogeneic recipients

While the Balb/c recipients are not given FLU themselves, the immunosuppressive effects of FLU on donor T cells lasts long after FLU has been removed. It is possible that FLU causes selection against allogeneic T cells, permanently deleting these cells from the recipients. To determine whether FLU pre-treated T cells retain *long-term* tolerance toward an allogeneic host, we transplanted Balb/c mice with vehicle or FLU-treated B6 donor cells, then performed *secondary* transplants into a second cohort of Balb/c mice. Given that neither the primary nor secondary recipients were given FLU directly, we reasoned that any short-term suppression of the donor T cells during the FLU pre-treatment should have worn off by the secondary transplantation. As in previous experiments, irradiated primary Balb/c recipients were transplanted with vehicle or FLU-treated CFP+ B6 cells (splenocytes and bone marrow). However,

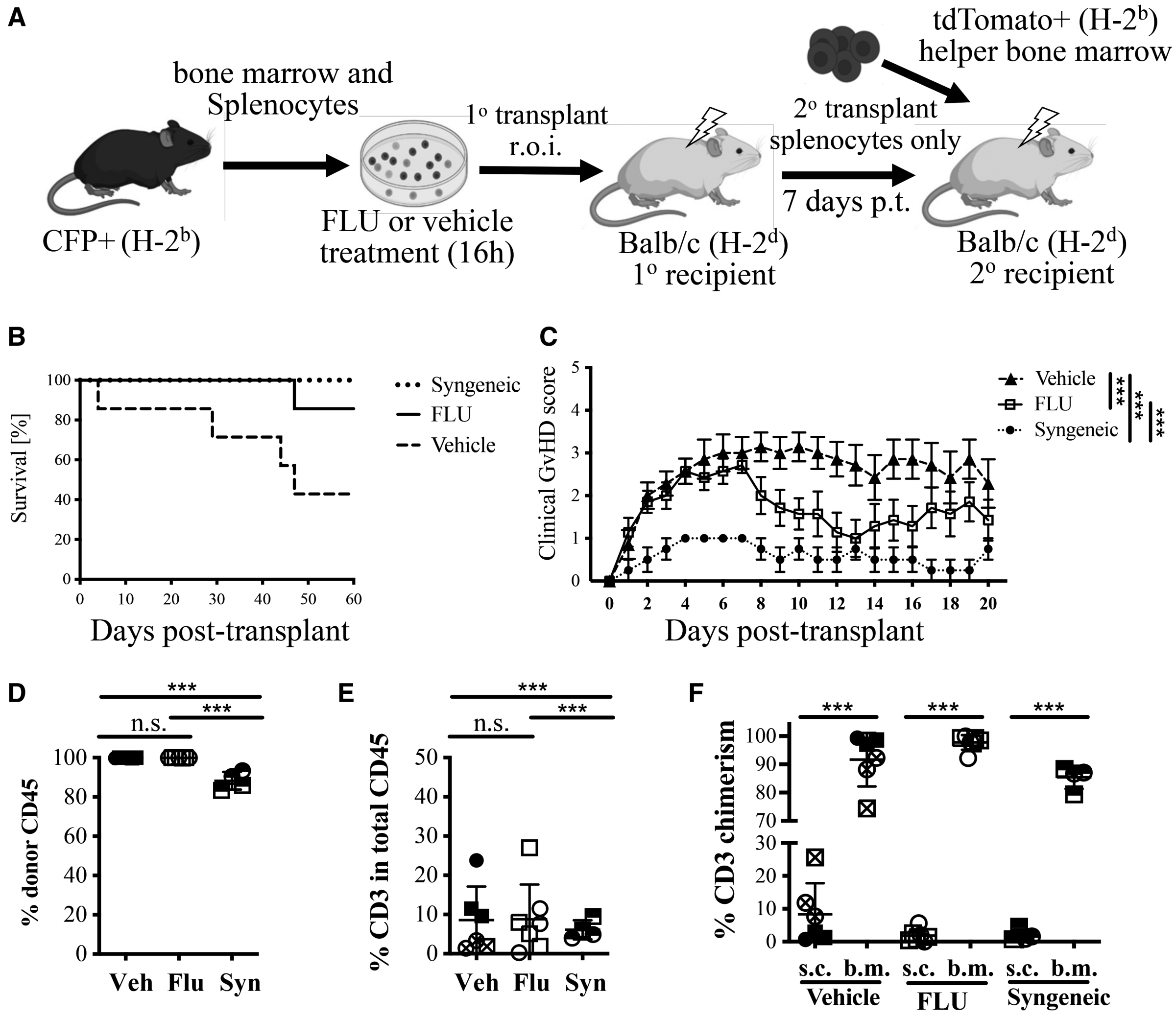

Figure 5. Analysis of GvHD after secondary allogeneic transplantation.

A Scheme of secondary allogeneic transplantations. CFP+ (H-2b) 3 × 10^6 whole BM plus 6 × 10^6 splenocytes pre-treated with FLU or Vehicle for 16 h were transplanted into lethally-irradiated Balb/c (H-2d) primary recipients. After 7 days, 1 × 10^6 splenocytes were harvested from the primary recipients and transplanted along with 2 × 10^6 tdTomato+ (H-2b) helper BM into lethally-irradiated Balb/c (H-2d) secondary recipients. For syngeneic transplants, both primary and secondary recipients were C57BL/6 (H-2b), and only vehicle-treated cells were used for the primary transplant.

B Survival study over a 60-day period post-transplantation of secondary recipients receiving allogeneic FLU (solid line) or Vehicle (dashed line) pre-treated primary recipient splenocytes ("FLU" n = 7, "Vehicle" n = 7, three male and four female; "Syngeneic", two male and two female; representative of two independent experiments).

C GvHD clinical grades of mice used in (B) over a period of 20 days (Error bars are SEM). *, ***$P \leq 0.001$ (Student's unpaired t-test).

D Percent of donor CD45+ chimerism including both primary recipient splenocytes (CFP+) and bone marrow derived (tdTomato+) cells in secondary recipients. Shown is the percentage of donor CD45+ cells out of total CD45+ cells.

E Percent total CD3+ donor T cells as a percentage of total live, CD45+ single cells. Donor T cells includes both splenocyte-derived (CFP+) and bone marrow derived (tdTomato+).

F Chimerism of donor primary splenocyte-derived (s.c., CFP+) or donor bone marrow-derived (b.m., tdTomato+) CD3+ T cells. Circles represent males and squares represent females. Crossed symbols represent mice later euthanized due to GvHD.

Data information: In D–F, FLU n = 7, three male and four female; Vehicle n = 6, three male and three female; Syngeneic vehicle (Syn or Syngeneic) n = 4, two male and two female; M ± SD). ***$P \leq 0.001$ (Student's unpaired t-test). r.o.i, retro-orbital injection, p.t., post-transplantation.
Source data are available online for this figure.

7 days post-transplantation, spleens from the primary recipients were harvested and transplanted along with 2 × 10^6 tdTomato+ helper BM (H-2b) into irradiated secondary Balb/c recipients (Fig 5A). For the syngeneic controls, both primary and secondary recipients were B6. After unblinding, only one FLU secondary transplanted recipient did not survive to 60 days post-transplant (who died due to a non-GvHD symptom) compared to the vehicle group which had less than half survive in that time span (Fig 5B). There

 © 2023 The Authors

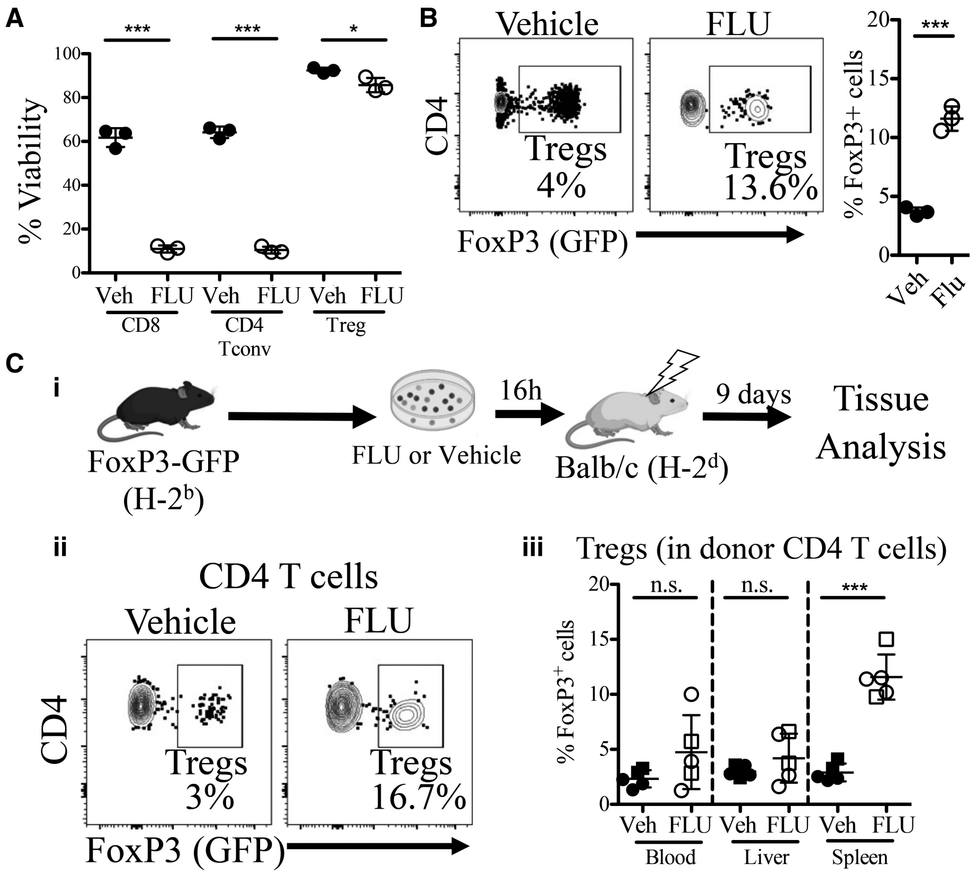

Figure 6. Analysis of FLU effects on regulatory and conventional CD4 T cells *in vitro* and after allogeneic transplantation.

A Flow cytometry analysis of FoxP3-GFP (B6-Foxp3^{EGFP}) spleen cells after 16 h culture in FLU or vehicle. Shown is the percentage of live (% Viability, Propidium Iodide (PI)$^{-}$, Annexin V^{-}) Tregs (CD3^{+}, CD4^{+}, FoxP3^{+}, single cells), CD4 conventional T cells (Tconv; CD3^{+}, CD4^{+}, Foxp3^{-}, single cells), and CD8 T cells (CD3^{+}, CD4^{+}, Foxp3^{-}, single cells; n = 3 biological replicates).

B Representative FACS plots (left) and quantification (right) showing the percentage of donor Tregs (CD4^{+}, Foxp3^{+}) as a percentage of total CD4 T cells (CD3^{+} CD4^{+}) after 16 h FLU (3 nM) or vehicle (DMSO) treatment (n = 3 biological replicates).

C (i) Scheme of allogeneic transplant of 6 × 10^{6} whole splenocytes plus 3 × 10^{6} whole BM cells from FoxP3-GFP (H-2^{b}) mice after culture with FLU or vehicle, then transplanted into lethally-irradiated (850 cGy, lightning symbol) Balb/c (H-2^{d}) recipients. Tissues (blood, spleen and liver) were analyzed 9 days after transplant. (ii) Representative gating scheme of donor Tregs (CD4^{+}, FoxP3^{+} (GFP^{+})), as a percentage of total donor CD4 T cells (H-2Db^{+}(H-2^{b+}), CD45^{+}, CD3^{+}, CD4^{+}, live, single cells) in recipient mouse spleens 9 days after transplant from FLU or vehicle pre-treated cells. (iii) Quantitative data from flow cytometry of the percent engrafted donor Tregs in recipient blood, liver and spleen as a percentage of donor CD4 T cells (n = 5 biological replicates).

Data information: M ± SD shown. Circles represent males and squares represent females. Veh., Vehicle, PI, Propidium iodide. *$P \leq 0.05$; ***$P \leq 0.001$ (Student's unpaired t-test).
Source data are available online for this figure.

was also a significant reduction in the clinical grade for "FLU" secondary recipients compared to "Vehicle" (Fig 5C). In the blood, nearly all recipients' T cells were TdTomato^{+} and therefore came from the transplanted bone marrow, indicating that any of the T cells from the original spleen culture did not expand in the secondary hosts (Fig 5D–F). This suggests that FLU treatment leads to long-lasting tolerance (or permanent depletion) of allogeneic T cells.

Regulatory T cells are resistant to FLU-induced apoptosis and had a higher engraftment in allogeneic recipient spleens after FLU treatment

As Tregs are known to reduce GvHD symptoms in mice (Riegel *et al*, 2020), we next assessed whether FLU had any impact on Treg numbers before or after allo-HCT (Fig 6). Compared to conventional CD4 T cells (CD4 Tconv), Tregs are more resistant to dexamethasone-induced apoptosis *in vitro* (Prenek *et al*, 2020). We measured cell viability after FLU or vehicle culture of splenocytes from B6-Foxp3^{EGFP} mice ("FoxP3-GFP"), in which Tregs are fluorescently labeled due to insertion of an eGFP reporter into the locus of the Treg-specific transcription factor FoxP3 (forkhead box protein P3, *Foxp3*), on a B6 (H-2^{b}) background (Haribhai *et al*, 2007; Lin *et al*, 2007). While B cells, CD8 T cells, and conventional (GFP^{-}) CD4 Tconv all had dramatically reduced viability after 16 h of treatment in FLU compared to Vehicle, Tregs (CD4^{+} GFP^{+}) displayed only a 10% drop in viability (Figs 6A and EV5A–C), which resulted in an increase in the ratio of Tregs to Tconv after FLU treatment, which could impact tolerance upon allo-HCT (Figs 6B and EV5D).

We next examined donor Treg and Tconv engraftment and expansion in the blood, liver, and spleens from recipient Balb/c mice 9 days after allogeneic transplantation of FLU- or vehicle-treated FoxP3-GFP donor cells (Fig 6Ci). There was a significantly higher percentage of engrafted donor Tregs in the spleens of recipients receiving FLU-treated donor cells (11.6% ± 2.0) compared to Vehicle (2.9% ± 0.80, Figs 6Cii–iii and EV5E). However, the differences in the blood and liver were not significant (Fig 6Ciii). In the spleen, the ratio of Treg:Tconv increased from approximately 1:33 (Vehicle) to 1:7.6 (FLU), about a 4.3-fold increase and similar to what was observed *in vitro*. These results were not due to a difference in donor engraftment, among different tissues or among vehicle and FLU-treated groups, indicated by a high CD45 donor chimerism (Fig EV5F). These results implicate a possible mechanism by which FLU reduces GvHD, by increasing the ratio of immunosuppressive Tregs to alloreactive Tconv in the graft.

Discussion

FLU pre-treatment of donor cells has implications in HCT therapy in two important aspects: (i) improving HSC engraftment efficiency, and (ii) reducing the incidence and/or severity of GvHD. In humans, the number of donor HSCs is critical to HCT success, thus increasing engraftment efficiency would be beneficial (Sugrue *et al*, 2000; Gordan *et al*, 2003; Pavone *et al*, 2006). Similar to what Guo *et al* showed using human CB HSCs, our results in mice showed similar improvements in CXCR4 expression, HSC migration, and HSC engraftment. Although mild, these results show that at the very least, culturing donor cells in FLU does not negatively affect HSC engraftment.

Regarding the second aspect, GvHD is a common life-threatening complication after allo-HCT. Our results in a mouse model of GvHD show a dramatic reduction in GvHD severity and death if the donor cells are first pre-treated with FLU prior to transplantation. While 100% of the recipient mice receiving vehicle-treated allogeneic cells died within 10 days, half of the mice receiving FLU-treated cells recovered and were essentially cured. We also observed a lower GvHD clinical grade in the FLU group, for both external signs of GvHD as well as in the gut. As many powerful glucocorticoids used to treat GvHD symptoms can have toxic side effects for patients, it is important to note that the mice examined in this study were never treated with FLU directly.

Regarding the mechanism for why FLU reduces GvHD, we observed robust T cell expansion in the “FLU” recipients, but a reduction in T cell activation, suggesting either an induction of tolerance, or a depletion of alloreactive T cells. The results in the mixed lymphocyte reaction support this notion, as exposure to allogenic cells was unable to stimulate and rescue FLU-treated T cells from apoptosis. Lastly, our findings of increased ratio of Tregs:Tconv could explain how FLU induces tolerance. Tregs appear unaffected by FLU, and their higher ratio in the recipient mice could prevent alloreactive Tconv from activation and response. The absence of alloactivation in FLU-treated T cells even after secondary allo-HCT indicates this suppression is permanent. We theorize that the balance of Treg:Tconv is critical to tolerance versus alloreactivity, and FLU treatment can shift this balance in favor of tolerance by increasing the relative number of Tregs to Tconv.

An important aspect to these findings is that FLU could potentially select against alloreactive T cells while maintaining some T cell functionality, which would be beneficial for preserving a GvL effect while simultaneously reducing GvHD. A previous study injected anti-CD4 antibodies to deplete donor $CD4^+$ T cells after allo-HCT and found a reduction in GvHD while preserving GvL (Ni *et al*, 2017). This suggests that $CD4^+$ T cells are necessary for GvHD but may be dispensable for GvL. We observed a significant decrease in the percentage of Th1 ($IFN\gamma^+$) CD4 T cells in the FLU group, indicative of a reduced inflammatory Th1 response (Fig 3G). Conversely, there was no reduction in the percentage of $IFN\gamma^+$ CD8 T cells. This suggests that the FLU-treated grafts may retain the ability to mount a CD8-driven response which could be important for GvL. That FLU-treated T cells can be activated *in vitro* with anti-CD3/CD28 antibodies supports the notion that these T cells may be activated under the appropriate conditions (Fig 4A). Future experiments will determine if FLU-treated grafts can still mount a robust GvL response.

Glucocorticoids have been used in many clinical treatments for decades. Fluticasone propionate (FLU) is commonly used in over-the-counter allergy nasal sprays. Corticosteroid therapy is also the first-line therapy to treat aGvHD after allo-HCT; however, this is a long-term treatment with many harmful side effects. Several clinical trials were conducted in the 1990s to investigate whether adding GCs (prednisone or methylprednisone) to the standard regimen of cyclosporine with or without methotrexate could prophylactically prevent development of GvHD in HLA-identical allo-HCT (Storb *et al*, 1990; Atkinson *et al*, 1991; Deeg *et al*, 1997; Chao *et al*, 2000; Ruutu *et al*, 2000). While some studies observed reductions in mild to moderate acute GvHD with the addition of GCs, others did not, and there appeared to be no improvements in incidence of severe acute GvHD or overall survival, and some studies reported increased incidence of chronic GvHD and/or infection. The general conclusion was that GCs administered to patients after allo-HCT but prior to the onset of GvHD symptoms provided no overall benefit to patients (Quellmann *et al*, 2008). Speculation for why GCs did not provide a benefit included decreased immune function and adverse interactions with methotrexate in patients receiving GCs. While discouraging for the use of GCs as a prophylactic to prevent GvHD, the key difference with our approach is that we are treating the donor cells with GCs prior to transplantation. Any side effects of giving GCs directly to patients are thus avoided. Whether this approach will be effective in human subjects remains to be seen, but our results clearly demonstrate a benefit of GC conditioning of donor cells to prevent and reduce acute GvHD in mouse models.

Materials and Methods

Detailed information about antibodies, mouse strains, fluorescence-activated cell sorting, HSC culture and chemotaxis assays, and T cell activation assays, are provided in the Appendix.

Mice

All strains were maintained at the Gross Hall and Med Sci A vivarium facilities at UCI and fed with standard chow and water. All animal procedures were approved by the International Animal Care

© 2023 The Authors

and Use Committee (IACUC) and University Laboratory Animal Resources (ULAR) of University of California, Irvine.

Competitive hematopoietic stem cell transplantation, and blood and bone marrow analysis

Sorted cells were cultured for 16 h in 37°C and 5% CO_2. A well of either FLU-treated or Vehicle-treated TM5 cells was combined 1:1 with a well containing vehicle-treated mTmG cells for a total of 2×10^4 KLS donor sorted cells per recipient. These cells were transplanted via retro-orbital injection into lethally-irradiated isoflurane-anesthetized gender matched C57BL/6 recipients. Lethal dose of X-ray irradiation was 850 cGy single dose (XRAD 320, Precision X-ray, North Branford, CT). Transplanted recipients were fed an antibiotic chow of Trimethoprim Sulfa (Uniprim, Envigo, East Millstone, NJ) for 3 weeks to prevent potential bacterial infections. For peripheral blood analysis, blood was obtained from the tail vein of transplanted mice at various time points, and red blood cells were depleted using ACK lysis buffer. For BM analysis, BM was harvested from tibias and femurs by flushing with ice-cold FACS buffer (PBS with 2% fetal bovine serum) followed by ACK lysis and filtration. BM HSC population is defined as single cell, PI^-, $Ter119^-$, $CD27^+$, c-kit^+, $Sca1^+$, $CD150^+$, cells with donor cells expressing CFP. Cells were analyzed on the BD Fortessa. Exclusion criteria include recipient mice without engraftment (chimerism below 1%), or recipients that died prior to analysis. FlowJo software (Tree Star) was used for data analysis.

GvHD cell culture and transplantation

A fully MHC mismatched allogeneic transplantation between mice with different haplotypes, H-2^b and H-2^d, was utilized as a mouse model for acute GvHD (aGvHD). Fresh bone marrow cells and splenocytes were harvested from H-2^b mice (either C57BL/6 or FoxP3-GFP) by flushing tibias and femurs using insulin syringes or using a cell homogenizer respectively. Red blood cells were lysed by ACK lysis buffer, then cells were filtered through a 70 μm mesh. Donor H-2^b cells were cultured with either 3 nM FLU or vehicle (DMSO) at a concentration of 6×10^6 splenocytes plus 3×10^6 whole bone marrow cells per well in a 24-well culture plate in 500 μl of serum-free X-VIVO 15 media. Cells were cultured for 16 h in 37°C and 5% CO_2.

For lineage cell viability analysis, unlike transplanted sample wells, splenocytes were cultured without bone marrow then collected and washed after incubation and stained for flow cytometry analysis. Different lineage cell markers were used depending on experiment (Appendix Table S1). Viability (percent live cells) was measured using the dead cell stain propidium iodide (PI) and the apoptotic cell marker Annexin V (Biolegend, #s 640905 and 640911) according to the manufacturer's protocol.

For transplantation, each well containing splenocytes and bone marrow cells was washed and resuspended in 100 μl of FACS buffer and injected retro-orbitally into lethally irradiated (850 cGy) isoflurane-anesthetized gender matched Balb/c (H-2^d) recipient mice or C57BL/6 (H-2^b) syngeneic control mice. Transplanted recipients were fed an antibiotic chow of Trimethoprim Sulfa (Uniprim, Envigo, East Millstone, NJ) to prevent potential bacterial infections. This transplantation was a blinded study where the treatment condition for recipient mice was unknown to the researcher until after data collection and analysis.

For secondary transplants, 1×10^6 whole splenocytes were taken 7 days post-transplantation from primary recipients, from the previously mentioned GvHD/ allogeneic transplantation protocol, along with fresh 2×10^6 helper $tdTomato^+$ (H-2^b) whole bone marrow and immediately transplanted into lethally irradiated (850 cGy) secondary Balb/ (H-2^d) allogeneic. For syngeneic transplantations, C57BL/6 (H-2^b) mice were used as recipients for both the primary and secondary transplantations. Exclusion criteria included recipient mice with poor or no engraftment (donor chimerism below 5% of total $CD45^+$ cells).

GvHD transplant tissue analysis

For splenocyte and liver analysis, tissues were harvested and homogenized with ice-cold FACS buffer in a glass tissue homogenizer. Next, red blood cells were ACK lysed and then filtered through a 70 μm mesh. Blood from the heart was collected in 10 mM EDTA solution immediately after euthanizing the recipient then ACK lysed and filtered. The following cell populations were analyzed in recipient blood, spleen and liver using flow cytometry: donor CD3 T cells (single cell, PI^-, $CD45^+$, CFP^+, Gr-1^-, $CD11b^-$, $B220^-$, $CD3^+$), CD4 T cells (single cell, PI^-, $CD45^+$, CFP^+, Gr-1^-, $CD11b^-$, $B220^-$, $CD3^+$, $CD8^-$, $CD4^+$), CD8 T cells (single cell, PI^-, $CD45^+$, CFP^+, Gr-1^-, $CD11b^-$, $B220^-$, $CD3^+$, $CD4^-$, $CD8^+$), macrophages (single cell, PI^-, $CD45^+$, CFP^+, Gr-1^-, $CD11b^+$), granulocytes (single cell, PI^-, $CD45^+$, CFP^+, $CD11b^-$, Gr-1^+) and B cells (single cell, PI^-, $CD45^+$, CFP^+, Gr-1^-, $CD11b^-$, $CD3^-$, $B220^+$). For BM analysis, BM was harvested from tibias and femurs by flushing with FACS buffer using an insulin syringe followed by ACK lysis and filtration. BM KLS population is defined as single cell, PI^-, $Ter119^-$, $CD27^+$, c-kit^+, $Sca1^+$ cells with donor cells expressing CFP. Cells were analyzed on the BD Fortessa. For more antibody information refer to Appendix Table S1. FlowJo software (Tree Star) was used for data analysis.

GvHD grading, external

Mice were observed daily for GvHD and scored according to a previously described scoring system (Naserian *et al*, 2018). Briefly, mice were examined for clinical signs of GvHD including hunched back, skin lesions, dull fur, diarrhea, and 10% loss of initial weight. A point was given for each symptom, with a maximum clinical score of 5. In addition, mice with greater than 30% weight loss from initial weight were immediately given a score of 5 and euthanized. Exclusion criteria included recipient mice with poor or no engraftment (donor chimerism below 5% of total $CD45^+$ cells).

GvHD grading, tissue sections

Small intestines and colon samples were harvested from mice and fixed in neutral buffered formalin overnight, then transferred to 100% ethanol for storage. Select samples were submitted to the UCI Chao Family Comprehensive Cancer Center (CFCCC) Experimental Tissue Shared Resource, where they were paraffin-embedded, sectioned, and hematoxylin and eosin (H&E) stained. Sections were examined and scored by a trained pathologist blinded to the identity

© 2023 The Authors

of the groups. The degree of histopathology observed was scored using a semi-quantitative scoring system (0–3 where 0 = normal, 1 = mild, 2 = moderate, and 3 = severe) for the following features as follows: Small intestine and colon: Lamina propria inflammation, crypt atrophy, and crypt epithelial apoptosis. Images were rendered using QuPath-0.2.2 software.

T cell stimulation and intracellular cytokine staining

Following transplantation of C57BL/6-CFP donor cells (bone marrow and spleen) into lethally-irradiated Balb/c recipients, animals were sacrificed 7–8 days post-transplantation, and the spleen and blood was collected for analysis of T cell subsets. After processing into single cell suspensions, spleen cells were divided into three tubes: the first tube was stained for external markers, and the other two were plated onto a 24-well plate, half were stimulated, and half were unstimulated. For the external markers, cells were stained with antibodies against CD45, CD3, CD4, CD8, B220, CD25, and PD1. CFP expression was used to assess donor chimerism, and only recipients with donor chimerism > 5%. Cultured cells were either unstimulated in complete RPMI buffer or stimulated by adding 1:500 dilution of the Cell Stimulation Cocktail (500×, Tonbo Biosciences, #TNB-4975) containing final concentrations of PMA (Phorbol 12-Myristate 13-Acetate, 81 nM), Ionomycin (1.34 μM), and protein transport inhibitors Brefeldin A (10.6 nM) and Monensin (2 nM), for 4 h in complete RPMI at 37°C and 5% CO_2. Following stimulation, cells were collected, washed and were resuspended in FACS buffer for intracellular staining analysis. For intracellular staining, cells were washed in FACS buffer, spun down at 300 RCF for 5 min, and resuspended in PBS with live/dead dye, Zombie Red or Zombie UV (Biolegend #s 423109 or 423107) 1:400 dilution for 5 min, per manufacturer's instructions. Cells were then washed with FACS buffer, centrifuged at 300 RCF for 5 min, and resuspended in a 1× fix/perm solution for 20 min using the FoxP3/Transcription Factor Staining Buffer Kit (TNB-0607-KIT, Tonbo). After fixation, cells were washed, centrifuged at 800 RCF for 5 min, and resuspended in 1× Perm buffer with antibodies against CD45, CD3, CD4, CD8, CD25, FoxP3, IL-4, IL-17, and IFNγ antibodies. Cells were stained overnight in an ice bucket in a 4°C cold room, then washed in 1× Perm Buffer, centrifuged at 800 RCF, and resuspended in FACS buffer. Cells were analyzed by flow cytometry on a BD Fortessa.

Data availability

The fcs files for the flow cytometry plots presented in the figures are available at FlowRepository.org under the repository ID FR-FCM-Z6KY. http://flowrepository.org/id/RvFr5FH6qO41yb7N1znr29qDhJc73q2NduzlLYThUtNLJuqkzIpCUrSGxRHdc1gj.

Expanded View for this article is available online.

Acknowledgements

The authors wish to thank Lok Tung Wong, Artemis Yip and all other members of the Inlay lab, past and present, for their intellectual support for this project, Mathew Blurton-Jones, Angela Fleischman, and Don Forthal for project advice, Rebecca Taylor for administrative support, Yumay Chen for lab support, Allia Fawaz for building management, and Chris and Sarah Veitch for project support. This study was supported primarily by a gift from the Oxnard Foundation (to MAI), with additional support from National Institutes of Health (NIH) grants R56HL133656 and R21CA224022 (to MAI), American Cancer Society ACS/IRG Seed Grant IRG 98-27-10 (to MAI), and California Institute for Regenerative Medicine grant CL1-00520-1.2 (to VMS and the UC Irvine Stem Cell FACS Core). LAA is supported by California Institute for Regenerative Medicine training grant EDUC4-12822, LAA and AK were supported in part from NIH grant R01AG055524, and ESV was supported by the Oxnard Foundation and NIH training grant T32AI060573 through the Institute for Immunology at UC Irvine. The authors also wish to acknowledge Dr. Robert Edwards for tissue analysis, Jeffrey Kim for sectioning and staining, and Delia Tifrea of the Chao Family Comprehensive Cancer Center (CFCCC) Experimental Tissue Shared Resource, which is supported by the National Cancer Institute (NCI) of the NIH under award number P30CA062203.

The paper explained

Problem

Graft versus Host Disease (GvHD) is a dangerous complication that can occur with allogeneic hematopoietic cell transplantation (allo-HCT). Glucocorticoids can suppress the immune response to alleviate GvHD symptoms but can have undesirable side effects. In this study, we sought to determine whether the pre-treatment of donor cells with glucocorticoids prior to transplantation could reduce the incidence and/or severity of GvHD in a mouse model.

Results

Our results showed that culturing mouse donor cells in the glucocorticoid fluticasone propionate (FLU) prior to transplantation could improve engraftment and reduce GvHD. Analysis of the immune repertoire showed that FLU could reduce the viability of conventional T cells and their activation after allo-HCT, particularly in the Th1 response. However, regulatory T cells were not affected, leading to an increase in the ratio of regulatory T cells to conventional T cells, which may condition the graft toward tolerance to the host.

Impact

These findings suggest a novel therapeutic strategy to condition donor grafts with glucocorticoids prior to transplantation to increase engraftment and reduce GvHD. As no direct drug administration to recipient mice was required for this effect, this could potentially reduce the need for immunosuppressive drugs for transplant patients.

Author contributions

Erika S Varady: Conceptualization; data curation; formal analysis; investigation; methodology; writing – original draft. **L Angel Ayala:** Conceptualization; data curation; formal analysis; validation; visualization; methodology; writing – review and editing. **Pauline U Nguyen:** Formal analysis; validation; methodology. **Vanessa M Scarfone:** Resources; methodology. **Alborz Karimzadeh:** Conceptualization; investigation. **Cuiwen Zhou:** Investigation; project administration. **Xiyu Chen:** Investigation. **Scott A Greilach:** Conceptualization; resources. **Craig M Walsh:** Conceptualization; resources; supervision. **Matthew A Inlay:** Conceptualization; data curation; formal analysis; supervision; funding acquisition; validation; investigation; visualization; methodology; writing – original draft; project administration; writing – review and editing.

 © 2023 The Authors

Disclosure and competing interests statement

All authors have read the journal's authorship agreement. The authors declare that they no conflict of interest.

References

Ashwell JD, Chen C, Schwartz RH (1986) High frequency and nonrandom distribution of alloreactivity in T cell clones selected for recognition of foreign antigen in association with self class II molecules. *J Immunol* 136: 389 – 395

Atkinson K, Biggs J, Concannon A, Dodds A, Young S, Wilson F, Ashby M, Downs K (1991) A prospective randomised trial of cyclosporin and methotrexate versus cyclosporin, methotrexate and prednisolone for prevention of graft-versus-host disease after HLA-identical sibling marrow transplantation for haematological malignancy. *Aust N Z J Med* 21: 850 – 856

Bahçeci E, Read EJ, Leitman S, Childs R, Dunbar C, Young NS, Barrett AJ (2000) CD34$^+$ cell dose predicts relapse and survival after T-cell-depleted HLA- identical haematopoietic stem cell transplantation (HSCT) for haematological malignancies. *Br J Haematol* 108: 408 – 414

Bair SM, Brandstadter JD, Ayers EC, Stadtmauer EA (2020) Hematopoietic stem cell transplantation for blood cancers in the era of precision medicine and immunotherapy. *Cancer* 126: 1837 – 1855

Beilhack A, Schulz S, Baker J, Beilhack GF, Wieland CB, Herman EI, Baker EM, Cao YA, Contag CH, Negrin RS (2005) *In vivo* analyses of early events in acute graft-versus-host disease reveal sequential infiltration of T-cell subsets. *Blood* 106: 1113 – 1122

Benichou G, Valujskikh A, Heeger PS (1999) Contributions of direct and indirect T cell alloreactivity during allograft rejection in mice. *J Immunol* 162: 352 – 358

Brenner S, Whiting-Theobald N, Kawai T, Linton GF, Rudikoff AG, Choi U, Ryser MF, Murphy PM, Sechler JMG, Malech HL (2004) CXCR4-transgene expression significantly improves marrow engraftment of cultured hematopoietic stem cells. *Stem Cells* 22: 1128 – 1133

Brunstein CG, Miller JS, Cao Q, McKenna DH, Hippen KL, Curtsinger J, DeFor T, Levine BL, June CH, Rubinstein P *et al* (2011) Infusion of *ex vivo* expanded T regulatory cells in adults transplanted with umbilical cord blood: safety profile and detection kinetics. *Blood* 117: 1061 – 1070

Chao NJ, Snyder DS, Jain M, Wong RM, Niland JC, Negrin RS, Long GD, Hu WW, Stockerl-Goldstein KE, Johnston LJ *et al* (2000) Equivalence of 2 effective graft-versus-host disease prophylaxis regimens: results of a prospective double-blind randomized trial. *Biol Blood Marrow Transplant* 6: 254 – 261

Cronstein BN, Aune TM (2020) Methotrexate and its mechanisms of action in inflammatory arthritis. *Nat Rev Rheumatol* 16: 145 – 154

Deeg HJ, Lin D, Leisenring W, Boeckh M, Anasetti C, Appelbaum FR, Chauncey TR, Doney K, Flowers M, Martin P *et al* (1997) Cyclosporine or cyclosporine plus methylprednisolone for prophylaxis of graft-versus-host disease: a prospective, randomized trial. *Blood* 89: 3880 – 3887

Di Ianni M, Falzetti F, Carotti A, Terenzi A, Castellino F, Bonifacio E, Del Papa B, Zei T, Iacucci Ostini R, Cecchini D *et al* (2011) Tregs prevent GVHD and promote immune reconstitution in HLA-haploidentical transplantation. *Blood* 117: 3921 – 3928

Edinger M, Hoffmann P, Ermann J, Drago K, Garrison Fathman C, Strober S, Negrin RS (2003) CD4$^+$CD25$^+$ regulatory T cells preserve graft-versus-tumor activity while inhibiting graft-versus-host disease after bone marrow transplantation. *Nat Med* 9: 1144 – 1150

El-Jawahri A, Li S, Antin JH, Spitzer TR, Armand PA, Koreth J, Nikiforow S, Ballen KK, Ho VT, Alyea EP *et al* (2016) Improved treatment-related mortality and overall survival of patients with grade IV Acute GVHD in the modern years. *Biol Blood Marrow Transplant* 22: 910 – 918

Fuji S, Byrne M, Nagler A, Mohty M, Savani BN (2021) How we can mitigate the side effects associated with systemic glucocorticoid after allogeneic hematopoietic cell transplantation. *Bone Marrow Transplant* 56: 1248 – 1256

Gluckman E, Rocha V, Boyer-Chammard A, Locatelli F, Arcese W, Pasquini R, Ortega J, Souillet G, Ferreira E, Laporte J-P *et al* (1997) Outcome of cord-blood transplantation from related and unrelated donors. *N Engl J Med* 337: 373 – 381

Gordan LN, Sugrue MW, Lynch JW, Williams KD, Khan SA, Wingard JR, Moreb JS (2003) Poor mobilization of peripheral blood stem cells is a risk factor for worse outcome in lymphoma patients undergoing autologous stem cell transplantation. *Leuk Lymphoma* 44: 815 – 820

Guo B, Huang X, Cooper S, Broxmeyer HE (2017) Glucocorticoid hormone-induced chromatin remodeling enhances human hematopoietic stem cell homing and engraftment. *Nat Med* 23: 424 – 428

Haribhai D, Lin W, Relland LM, Truong N, Williams CB, Chatila TA (2007) Regulatory T cells dynamically control the primary immune response to foreign antigen. *J Immunol* 178: 2961 – 2972

Hendrikx PJ, Martens ACM, Hagenbeek A, Keij JF, Visser JWM (1996) Homing of fluorescently labeled murine hematopoietic stem cells. *Exp Hematol* 24: 129 – 140

Ho VT, Soiffer RJ (2001) The history and future of T-cell depletion as graft-versus-host disease prophylaxis for allogeneic hematopoietic stem cell transplantation. *Blood* 98: 3192 – 3204

Hoffmann P, Ermann J, Edinger M, Garrison Fathman C, Strober S (2002) Donor-type CD4$^+$CD25$^+$ regulatory T cells suppress lethal acute graft-versus-host disease after allogeneic bone marrow transplantation. *J Exp Med* 196: 389 – 399

Irene GC, Albert E, Anna BV, Rahinatu A, Silvana N, Silvana S, Ana G, Jordi L, Carolina CA, Miquel G *et al* (2021) Patterns of infection and infectious-related mortality in patients receiving post-transplant high dose cyclophosphamide as graft-versus-host-disease prophylaxis: impact of HLA donor matching. *Bone Marrow Transplant* 56: 818 – 827

Iscove NN, Nawa K (1997) Hematopoietic stem cells expand during serial transplantation in vive without apparent exhaustion. *Curr Biol* 7: 805 – 808

Jagasia M, Arora M, Flowers MED, Chao NJ, McCarthy PL, Cutler CS, Urbano-Ispizua A, Pavletic SZ, Haagenson MD, Zhang MJ *et al* (2012) Risk factors for acute GVHD and survival after hematopoietic cell transplantation. *Blood* 119: 296 – 307

Khoury HJ, Wang T, Hemmer MT, Couriel D, Alousi A, Cutler C, Aljurf M, Battiwalla M, Cahn JY, Cairo M *et al* (2017) Improved survival after acute graft-versus-host disease diagnosis in the modern era. *Haematologica* 102: 958 – 966

van Leeuwen L, Guiffre A, Atkinson K, Rainer SP, Sewell WA (2002) A two-phase pathogenesis of graft-versus-host disease in mice. *Bone Marrow Transplant* 29: 151 – 158

Lin W, Haribhai D, Relland LM, Truong N, Carlson MR, Williams CB, Chatila TA (2007) Regulatory T cell development in the absence of functional Foxp3. *Nat Immunol* 8: 359 – 368

Luznik L, Jalla S, Engstrom LW, Lannone R, Fuchs EJ (2001) Durable engraftment of major histocompatibility complex-incompatible cells after nonmyeloablative conditioning with fludarabine, low-dose total body irradiation, and posttransplantation cyclophosphamide. *Blood* 98: 3456 – 3464

© 2023 The Authors

Luznik L, O'Donnell PV, Symons HJ, Chen AR, Leffell MS, Zahurak M, Gooley TA, Piantadosi S, Kaup M, Ambinder RF *et al* (2008) HLA-haploidentical bone marrow transplantation for hematologic malignancies using nonmyeloablative conditioning and high-dose, posttransplantation cyclophosphamide. *Biol Blood Marrow Transplant* 14: 641 – 650

MacMillan ML, Hippen KL, McKenna DH, Kadidlo D, Sumstad D, Defor TE, Brunstein CG, Holtan SG, Miller JS, Warlick ED *et al* (2021) First-in-human phase 1 trial of induced regulatory T cells for graft-versus-host disease prophylaxis in HLA-matched siblings. *Blood Adv* 5: 1425 – 1436

Martelli MF, Di Ianni M, Ruggeri L, Pierini A, Falzetti F, Carotti A, Terenzi A, Reisner Y, Aversa F, Falini B *et al* (2014) 'Designed' grafts for HLA-haploidentical stem cell transplantation. *Blood* 123: 967 – 973

Modi D, Albanyan O, Kim S, Deol A, Alavi A, Kin AD, Ayash LJ, Ratanatharathorn V, Uberti JP (2020) Toxicities after high dose post-transplant cyclophosphamide in haploidentical donor transplants: risk factors and impact on survival. *J Clin Oncol* 38: 7545

Naserian S, Leclerc M, Thiolat A, Pilon C, Le Bret C, Belkacemi Y, Maury S, Charlotte F, Cohen JL (2018) Simple, reproducible, and efficient clinical grading system for murine models of acute graft-versus-host disease. *Front Immunol* 9: 10

Ni X, Song Q, Cassady K, Deng R, Jin H, Zhang M, Dong H, Forman S, Martin PJ, Chen YZ *et al* (2017) PD-L1 interacts with CD80 to regulate graft-versusleukemia activity of donor $CD8^+$ T cells. *J Clin Investig* 127: 1960 – 1977

van Os R, Ausema A, Dontje B, van Riezen M, van Dam G, de Haan G (2010) Engraftment of syngeneic bone marrow is not more efficient after intrafemoral transplantation than after traditional intravenous administration. *Exp Hematol* 38: 1115 – 1123

Pavone V, Gaudio F, Console G, Vitolo U, Iacopino P, Guarini A, Liso V, Perrone T, Liso A (2006) Poor mobilization is an independent prognostic factor in patients with malignant lymphomas treated by peripheral blood stem cell transplantation. *Bone Marrow Transplant* 37: 719 – 724

Peled A, Petit I, Kollet O, Magid M, Ponomaryov T, Byk T, Nagler A, Ben-Hur H, Many A, Shultz L *et al* (1999) Dependence of human stem cell engraftment and repopulation of NOD/SCID mice on CXCR4. *Science* 283: 845 – 848

Prenek L, Litvai T, Balázs N, Kugyelka R, Boldizsár F, Najbauer J, Németh P, Berki T (2020) Regulatory T cells are less sensitive to glucocorticoid hormone induced apoptosis than $CD4^+$ T cells. *Apoptosis* 25: 715 – 729

Quellmann S, Schwarzer G, Hübel K, Engert A, Bohlius J (2008) Corticosteroids in the prevention of graft-vs-host disease after allogeneic myeloablative stem cell transplantation: a systematic review and meta-analysis. *Leukemia* 22: 1801 – 1803

Ratajczak MZ, Suszynska M (2016) Emerging strategies to enhance homing and engraftment of hematopoietic stem cells. *Stem Cell Rev Rep* 12: 121 – 128

Riegel C, Boeld TJ, Doser K, Huber E, Hoffmann P, Edinger M (2020) Efficient treatment of murine acute GvHD by *in vitro* expanded donor regulatory T cells. *Leukemia* 34: 895 – 908

Ruutu T, Volin L, Parkkali T, Juvonen E, Elonen E (2000) Cyclosporine, methotrexate, and methylprednisolone compared with cyclosporine and methotrexate for the prevention of graft-versus-host disease in bone marrow transplantation from HLA-identical sibling donor: a prospective randomized study. *Blood* 96: 2391 – 2398

Ruutu T, Gratwohl A, De Witte T, Afanasyev B, Apperley J, Bacigalupo A, Dazzi F, Dreger P, Duarte R, Finke J *et al* (2014) Prophylaxis and treatment of GVHD: EBMT-ELN working group recommendations for a standardized practice. *Bone Marrow Transplant* 49: 168 – 173

Schumann J, Stanko K, Schliesser U, Appelt C, Sawitzki B (2015) Differences in CD44 surface expression levels and function discriminates IL-17 and IFN-γ producing helper T cells. *PloS One* 10: e0132479

Sharma M, Afrin F, Satija N, Tripathi RP, Gangenahalli GU (2011) Stromal-derived factor-1/CXCR4 signaling: indispensable role in homing and engraftment of hematopoietic stem cells in bone marrow. *Stem Cells Dev* 20: 933 – 946

Storb R, Deeg HJ, Whitehead J, Appelbaum F, Beatty P, Bensinger W, Buckner CD, Clift R, Doney K, Farewell V *et al* (1986) Methotrexate and cyclosporine compared with cyclosporine alone for prophylaxis of acute graft versus host disease after marrow transplantation for leukemia. *N Engl J Med* 314: 729 – 735

Storb R, Pepe M, Anasetti C, Appelbaum FR, Beatty P, Doney K, Martin P, Stewart P, Sullivan KM, Witherspoon R *et al* (1990) What role for prednisone in prevention of acute graft-versus-host disease in patients undergoing marrow transplants? *Blood* 76: 1037 – 1045

Suchin EJ, Langmuir PB, Palmer E, Sayegh MH, Wells AD, Turka LA (2001) Quantifying the frequency of alloreactive T cells *in vivo*: new answers to an old question. *J Immunol* 166: 973 – 981

Sugrue MW, Williams K, Pollock BH, Khan S, Peracha S, Wingrad JR, Moreb JS (2000) Characterization and outcome of 'hard to mobilize' lymphoma patients undergoing autologous stem cell transplantation. *Leuk Lymphoma* 39: 509 – 519

Zorn E, Kim HT, Lee SJ, Floyd BH, Litsa D, Arumugarajah S, Bellucci R, Alyea EP, Antin JH, Soiffer RJ *et al* (2005) Reduced frequency of $FOXP3^+$ $CD4^+CD25^+$ regulatory T cells in patients with chronic graft-versus-host disease. *Blood* 106: 2903 – 2911

License: This is an open access article under the terms of the Creative Commons Attribution License, which permits use, distribution and reproduction in any medium, provided the original work is properly cited.

Expanded View Figures

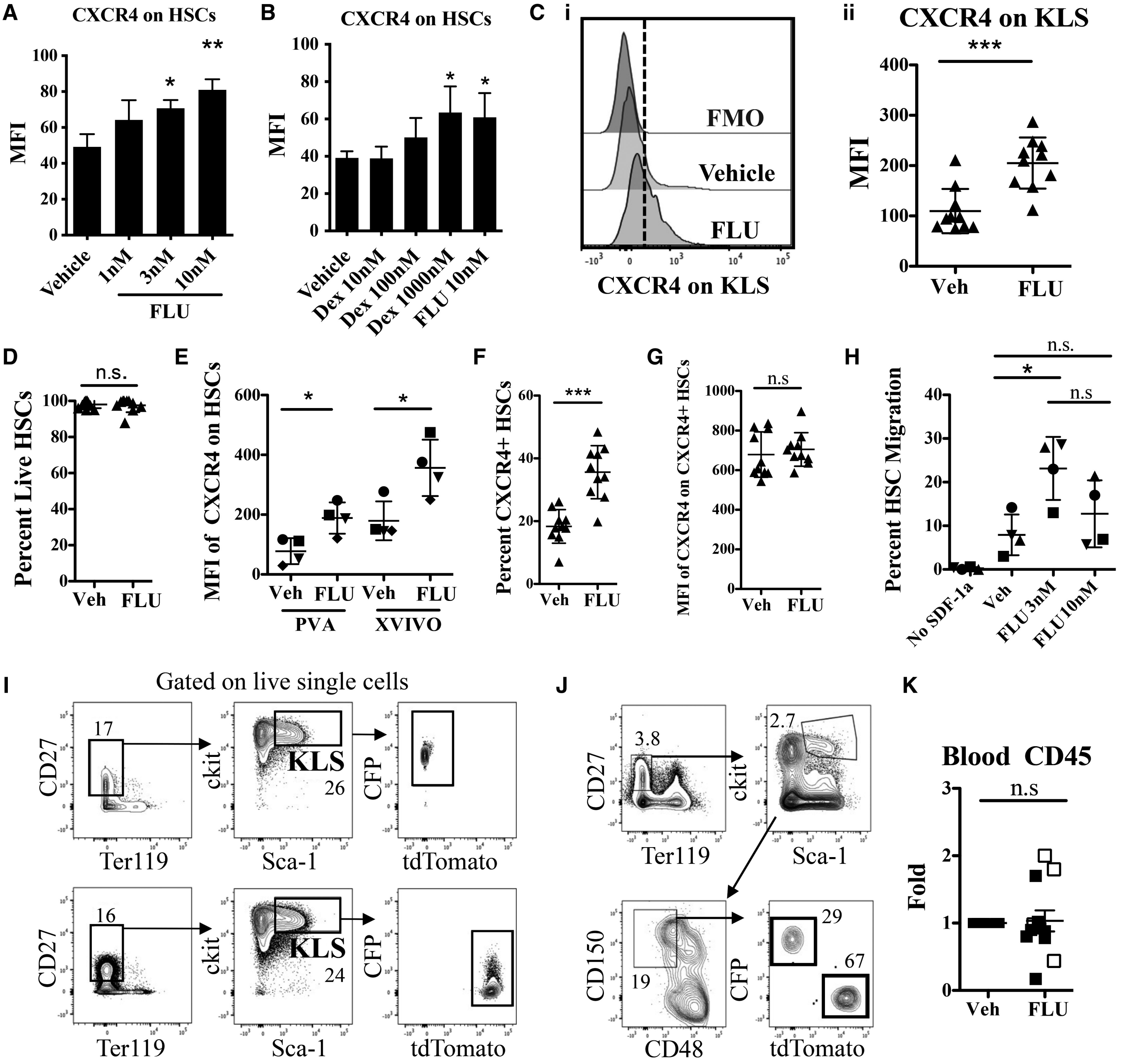

Figure EV1.

Figure EV1. Characterizing murine cells after FLU treatment.

A, B Comparisons of MFI of CXCR4 surface expression on HSCs treated with Vehicle and three different doses of (A) FLU (1, 3, and 10 nM) and (B) Dexamethasone (Dex, 10, 100, and 1,000 nM), $n = 3$. Both 3 nM and 10 nM FLU had significant increases in CXCR4 compared to vehicle, but not relative to each other. Only 1,000 nM Dex led to a significant increase in CXCR4 expression relative to vehicle, and similar to 10 nM FLU.

C (i) Representative histograms of CXCR4 expression on KLS population after FLU (blue) and Vehicle (DMSO, red) culture conditions. The dashed line indicates cutoff for positive CXCR4 expression based on the fluorescence minus one (FMO, gray histogram) control. (ii) MFI of cell surface CXCR4 on KLS population after culture conditions ($n = 10$, representative of seven independent experiments). Data indicates an approximately 2-fold increase of CXCR4 expression on KLS cells after FLU treatment relative to vehicle control similar to that observed in HSCs in Fig 1A.

D Total live HSCs after culture conditions indicates that 16 h culture in 3 nM FLU does not negatively affect HSC viability.

E MFI of CXCR4 on HSCs treated with FLU or Vehicle cultured in two different mediums, either serum-free XVIVO medium or polyvinyl alcohol (PVA) supplemented medium ($n = 4$, representative of four independent experiments). This shows that the FLU-induced increase in CXCR4 expression is not specific to one type of cell culture media.

F Percent CXCR4 positive HSCs.

G MFI of CXCR4 on CXCR4^{+} HSC population. These data indicate that CXCR4 expression is not increasing on CXCR4^{+} HSCs, but rather that CXCR4^{-} HSCs begin to turn on CXCR4. D, F, and G: $n = 10$, representative of seven independent experiments.

H HSC migration toward SDF-1α (50 ng/ml) after treatment of Vehicle or two different concentrations of FLU (3 or 10 nM) quantified by flow cytometry ($n = 4$, representative of four independent experiments). Migration appeared reduced in HSCs treated in 10 nM FLU compared to 3 nM.

I Representative sorting scheme of c-kit^{+}, CD27^{+}, Sca-1^{+} (KLS) live single cells from either CFP^{+} (top) or tdTomato^{+} (bottom) c-kit enriched BM, which were then cultured in vehicle or FLU then used in the transplantation experiments shown in Fig 1D.

J Representative analysis of donor HSCs (CD27^{+} Ter119^{-}, c-kit^{+}, Sca-1^{+}, CD150^{+}, CD48^{-}) from competitive transplanted recipient BM 12 weeks post-transplant. Recipient from "Vehicle" control group is shown.

K Fold change in blood CD45 chimerism 12 weeks post-transplantation normalized to Vehicle group ($n = 12$, representative of nine independent experiments). Open symbols are females.

Data information: $*P \le 0.05$; $**P \le 0.01$; $***P \le 0.001$ (Student's unpaired t-test). $M \pm$ SD shown. HSC, Hematopoietic stem cell, KLS, c-kit^{+} lineage^{-} sca-1^{+} cell population, Dex, Dexamethasone, Flo., FLU, Veh., Vehicle, FMO, fluorescence minus one.

 © 2023 The Authors

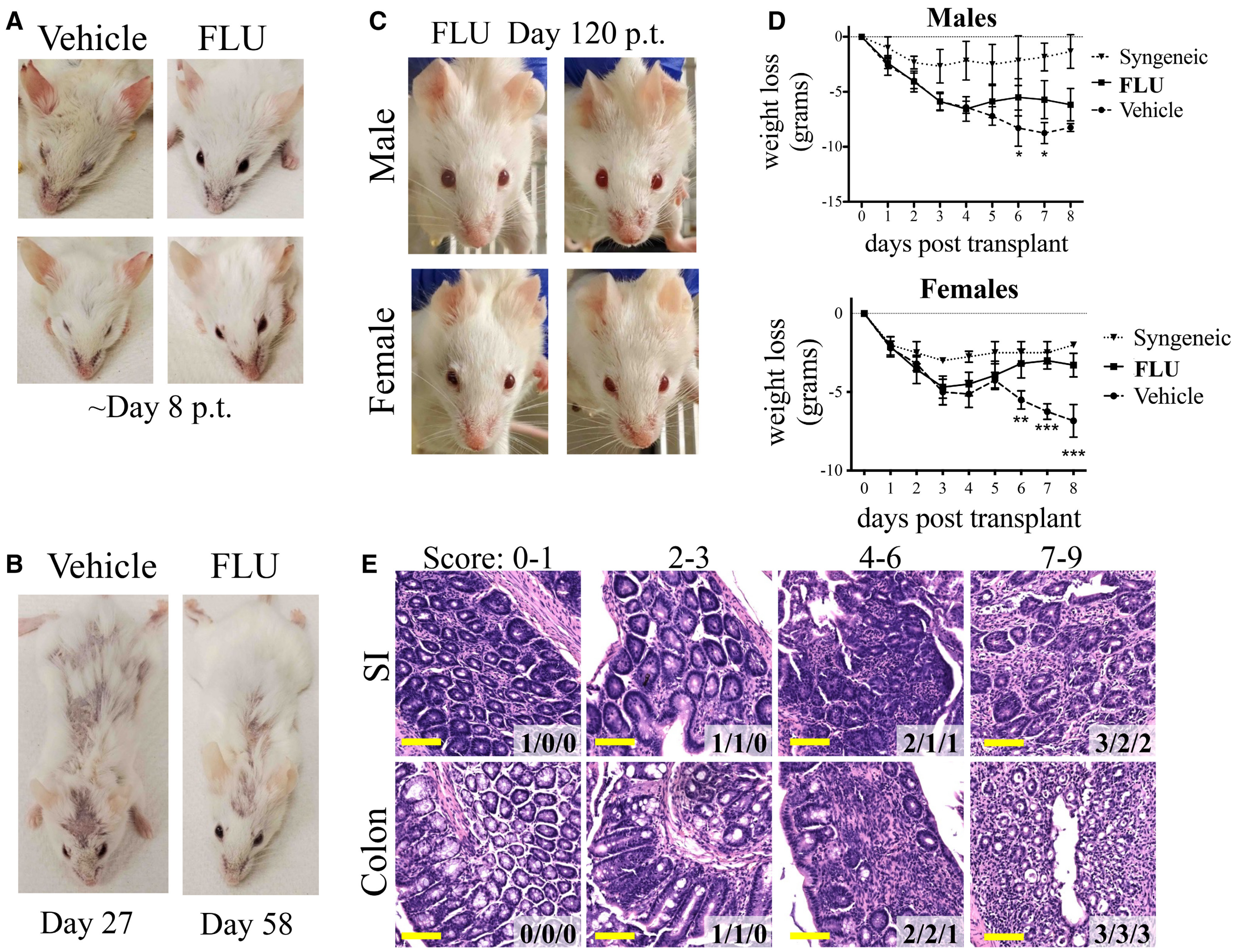

Figure EV2. Mice that received FLU-treated cells show less symptoms of GvHD compared to mice that received Vehicle-treated cells.

A Additional representative photographs of FLU and Vehicle treated cell recipients after approximately 7 days. Mice that were photographed were randomly chosen. Photos are taken of males except for the bottom left photograph is a female.

B Images of euthanized mice in each group at a later stage with skin lesions. Both are males.

C Images from remaining live healthy mice from the FLU-treated transplantation group approximately 120 days post-transplantation.

D Weight loss analysis of allogeneic transplants “FLU” and “Vehicle”, and “Syngeneic” vehicle (C57BL/6 into C57BL/6) transplanted mice separating males (top; $n = 7$ for “Vehicle”, $n = 8$ for “FLU”, $n = 3$ for “Syngeneic” groups) from females (bottom; $n = 4$ for “Vehicle”, $n = 8$ for “FLU”, $n = 2$ for “Syngeneic” groups). Significance is Vehicle group comparison to FLU group. Males at day 8 only had an n of 2.

E Comparison of small intestines (SI, top row), and colon (bottom row) of H&E stained sections representing different histopathological clinical scores. Tissues were scored by a blinded pathologist and assigned scores from 0 to 3 for three clinical signs of GvHD histopathology: lamina propria inflammation, crypt atrophy, and crypt epithelial apoptosis. Images represent different combined scores from 0 to 1 (leftmost), 2 to 3 (middle left), 4 to 6 (middle right), and 7 to 9 (rightmost). The three scores for each tissue is displayed in the lower right corner. Note that images represent only one region of the tissue, and tissues with lower scores had more regions of no pathology than those with higher scores. Yellow scale bars equal 100 microns.

Data information: $^{*}P \leq 0.05$; $^{**}P \leq 0.01$; $^{***}P \leq 0.001$; Student’s unpaired t-test. $M \pm$ SD shown. p.t., post-transplant.

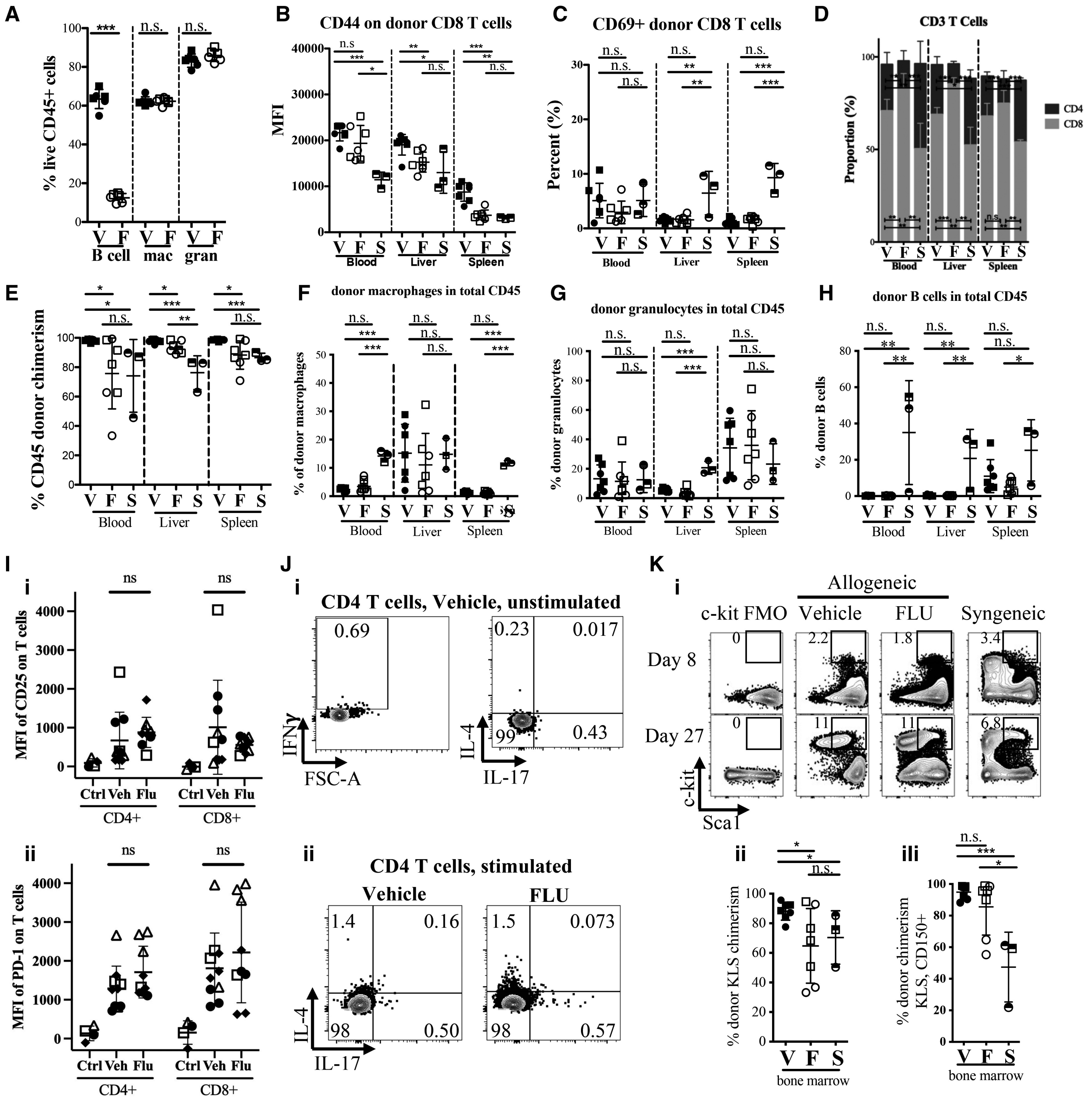

Figure EV3.

© 2023 The Authors

Figure EV3. Additional characterization of donor cells in allogeneic transplanted recipients receiving either FLU or Vehicle-treated whole splenocytes and bone marrow cells.

A Quantification of live B cells (CD45$^+$, CD11b$^-$, Gr1$^-$, CD3$^-$, B220$^+$), macrophages (mac; CD45$^+$, Gr1$^-$, CD11b$^+$), and granulocytes (gran; CD45$^+$, CD11b$^+$, Gr-1$^+$) after 16 h culture with FLU or Vehicle (n = 6, representative of three independent experiments). FLU dramatically decreases B cell viability but does not affect myeloid populations.

B, C Quantification of percent CD69$^+$ cells (B) and MFI of CD44 expression (C) in donor CD8 population. Gated on live, CD45$^+$, CFP$^+$, CD11b$^-$, Gr1$^-$, B220$^-$, CD3$^+$, CD4$^-$, CD8$^+$ single cells. No significant differences were observed in CD69 or CD44 expression in CD8 T cells.

D Proportion (%) of CD4 (purple) and CD8 (gray) T cells within the CD3$^+$ T cell population. Overall, FLU-treated donors cells had a decrease in CD4 T cells compared to Vehicle-treated donor cells, though both FLU and Vehicle groups had a lower CD4:CD8 ratio than the syngeneic recipients.

E Total donor CD45$^+$ chimerism in blood, liver and spleen of transplanted recipients. Data is donor (CFP$^+$) CD45$^+$ cells as a percentage of total CD45$^+$ cells. At 1 week post-transplant, donor chimerism is slightly lower in the FLU group compared to vehicle, though this could be due to the lower number of viable cells transplanted after FLU culture.

F–H Quantification of percent donor macrophages (F) granulocytes (G) and B cells (H) in total CD45 cells. No significant differences between FLU and Vehicle groups were observed.

I Quantification of surface CD25 (i) and PD1 (ii) MFI expression in donor-derived (CFP$^+$) CD4 and CD8 T cells, 7–8 days after transplantation. Different symbols represent four independent experiments, open symbols are females, closed symbols are male, Ctrl are fresh B6-CFP spleen cells. While CD25 and PD1 are both upregulated in donor-derived cells 7–8 days after allogeneic transplantation, there is no significant difference between Vehicle and FLU-treated grafts.

J (i) IFNγ, IL-4, and IL-17 expression levels in unstimulated CD4 T cells from Vehicle recipient. These plots were used as a negative control for gating for the data in Fig 3E. (ii) Representative FACS plot of IL-4 and IL-17 expression of stimulated CD4 T cells, quantified in Fig 3Eiii. Please note that the data represented in Figs 3E and EV3I and J are from the same set of experiments.

K (i) Representative plot of donor (CFP$^+$) KLS population in BM from transplanted recipients of Vehicle-treated, FLU-treated, and Vehicle-treated-syngeneic donor cells from mice euthanized on day 8 p.t. (top) and on day 27 p.t (bottom). Donor KLS (ii) and KLS, CD150$^+$ (iii) chimerism in recipient BM after transplant. Data indicate a slight reduction in engraftment of FLU-treated BM compared to Vehicle-treated, but not in the CD150$^+$ KLS population, which is more highly enriched for HSCs. B-H: n = 7 for FLU and Vehicle groups days 6–9 p.t., representative of three independent experiments, n = 3 for Syngeneic Vehicle-treated (S) CFP$^+$ C57BL/6 into C57BL/6 group day 9 (1 mouse) and day 27 (2 mice) p.t., representative of two independent experiments. Circles represent males and squares represent females.

Data information: *$P \le 0.05$; **$P \le 0.01$; ***$P \le 0.001$ (Student's unpaired t-test). Error bars are SD. p.t., post-transplant, KLS, c-kit$^+$ lineage$^-$ Sca1$^+$ cell population, FMO, fluorescence minus one, V, Vehicle, F, FLU, S, syngeneic, MFI, mean fluorescent intensity, n.s. not significant.

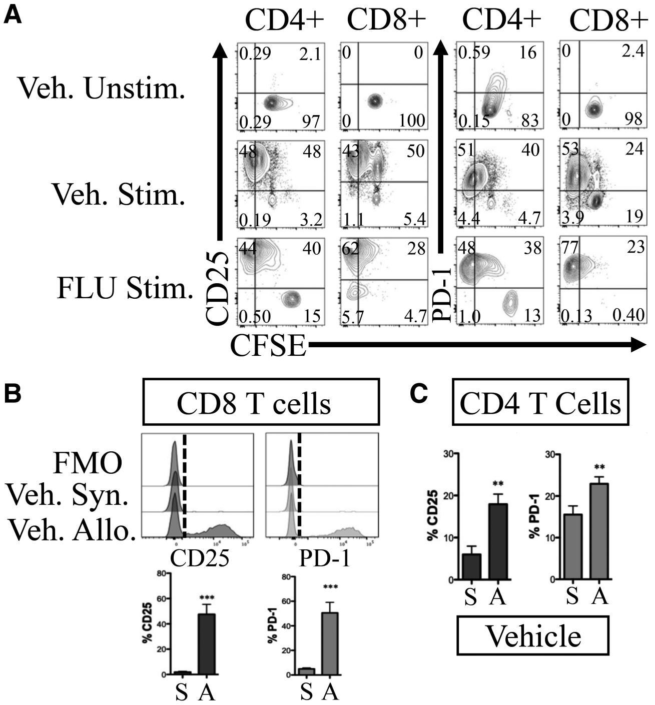

Figure EV4. Additional analysis of FLU-treated splenocytes after CD3/CD28 stimulation and MLR.

A Representative plots of proliferation (CFSE staining) vs activation markers CD25 and PD-1 expression on either FLU or Vehicle pretreated CD4$^+$ and CD8$^+$ cells after *in vitro* T cell activation assay via anti-CD3/CD28 antibody stimulation, from Fig 4A. Cells were stained with CFSE after FLU/vehicle culture and prior to anti-CD3/CD28 stimulation. In both vehicle and FLU conditions, stimulated cells diluted CFSE and upregulated CD25 and PD-1, indicating that anti-CD3/CD28 stimulation could rescue FLU-treated T cells from apoptosis and induce activation and proliferation.

B, C Representative histograms and quantification from MLR of CD25 and PD-1 expression on CD8 T cells (B) and CD4 T cells (C), from Fig 4B. Plots are gated on live, single, CD3$^+$ cells and either CD4$^+$ or CD8$^+$. Bar graphs display the percentages of CD25$^+$ or PD-1$^+$ T cells. **$P \le 0.01$; ***$P \le 0.001$ (Student's unpaired t-test). Syn. or S., Syngeneic, Allo. or A., Allogeneic, Veh., Vehicle, Unstim., unstimulated, Stim., stimulated, Flo, FLU, FMO, fluorescence minus one.

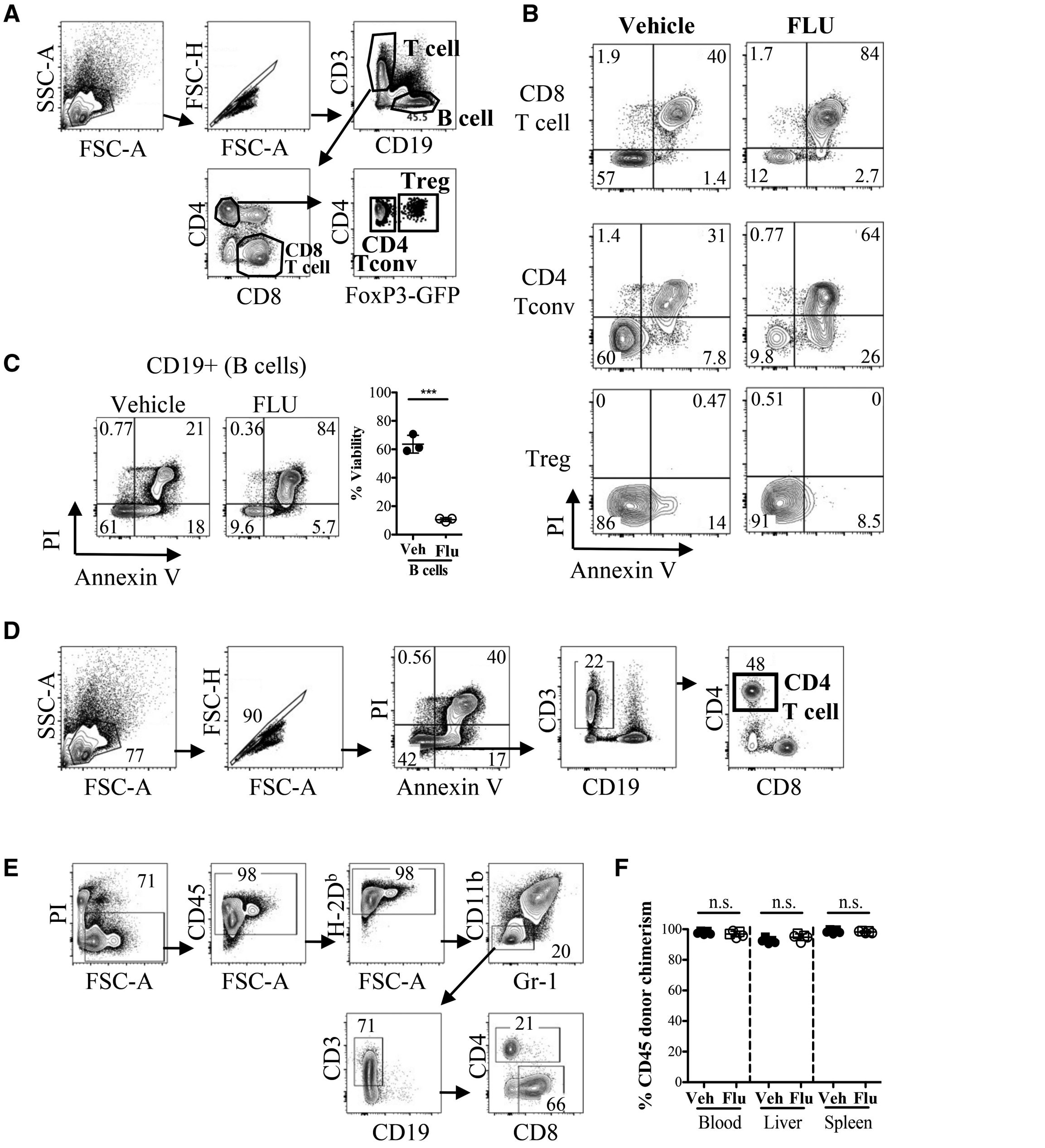

Figure EV5. Additional characterization of Tregs and other lymphocytes after FLU treatment *in vitro* and after allogeneic transplantation.

A Representative FACS gating scheme of FoxP3-GFP splenocytes after 16 h of treatment with FLU (3 nM) or vehicle (DMSO) before gating on viability using Propidium Iodide (PI) and apoptotic marker Annexin V, used in Fig 6A. Only vehicle treated cells are shown.

B Representative FACS plots showing live (PI^-, Annexin V^-), apoptotic (PI^-, Annexin V^+), and dead (PI^+, Annexin V^+) CD8 T cells, CD4 Tconv, and Tregs after 16 h FLU or vehicle culture. Data used for quantification in Fig 6A.

C Representative FACS plots (left) and quantitative data (right) showing B cell ($CD19^+$) viability ($n = 3$). B cell viability is poor after FLU treatment, with similar viability to that of CD4 Tconv and CD8 T cells.

D Representative FACS gating scheme of splenocytes after 16 h treatment with FLU or vehicle after gating on live (PI^-, Annexin V^-) cells. Data used for quantification in Fig 6B. Only vehicle treated cells shown. The same fcs files were used in EV5A.

E Representative FACS gating scheme of lineage cells from allogeneic recipient Balb/c ($H\text{-}2^d$) mice (spleen shown) 9 days after transplantation with donor FoxP3-GFP ($H\text{-}2^b$) mouse splenocytes and bone marrow. Data used for quantification in Fig 6C.

F Percent CD45 donor FoxP3-GFP ($H\text{-}2^b$) chimerism ($CD45^+$ $H\text{-}2D^{b+}$) as a percentage of total $CD45^+$ cells in recipient Balb/c ($H\text{-}2^d$) blood, liver and spleen 9 days post-transplantation ($n = 5$). Near 100% donor chimerism indicates a successful engraftment and hematopoietic reconstitution. n.s., not significant (Student's unpaired *t*-test).

 © 2023 The Authors

APPENDIX

Graft pre-treatment with fluticasone propionate reduces graft-versus-host disease in allo-HCT

Erika S. Varady[1,2,*], L. Angel Ayala[1,2,*], Pauline U. Nguyen[1,2], Vanessa M. Scarfone[1], Alborz Karimzadeh[1,2,**], Cuiwen Zhou[1,2], Xiyu Chen[1,2], Scott A. Greilach[1,2], Craig M. Walsh[1,2], and Matthew A. Inlay[1,2,†]

Table of Contents

Appendix Methods 2

Appendix Table S1. Antibodies 5

Appendix References 6

Appendix Methods

Antibodies

A detailed list of all antibodies used in this study is shown in Table S1.

Mice

C57BL/6J (H-2^b, "B6", stock no. 00664), mTmG (H-2^b, "tdTomato+", stock no. 007576), B6-Foxp3^{EGFP} (H-2^b, "FoxP3-GFP", stock no. 006772) and Balb/cJ (H-2^d, "Balb/c", stock no. 000651) mouse strains from Jackson Laboratory (Bar Harbor, ME) 8-12 weeks of age along with Rosa-ECFP (H-2^b, "CFP" or "TM5") mice 8-12 weeks of age generously donated by Dr. Irving Weissman, were used as donors and recipients for competitive transplantation. mTmG, FoxP3-GFP, and CFP are all congenic strains on the B6 background.

HSC Cell Culture for CXCR4 analysis

BM was harvested from C57BL/6 mice by crushing tibias and femurs using a mortar and pestle. Cells were processed in ice-cold fluorescence activated cell sorting (FACS) buffer (phosphate buffered saline (PBS) + 2% fetal bovine serum (FBS)). Red blood cells were lysed by ACK lysis buffer (155 mM NH_4Cl, 10 mM $KHCO_3$, 0.1 mM Na_2EDTA, pH 7.2-7.4 in dH2O) then cells were filtered through a 70μ mesh (Genesee Scientific, CA, #57-104). Cells were then resuspended in 100μL FACS buffer and incubated with 5μL of mouse anti-Kit (anti-CD117) Microbeads (Miltenyi Biotec, #130-091-224) for 15 minutes then magnetic separation was performed using the Milyenyi AutoMACs Pro cell separator (Miltenyi Biotec, Somerville, MA). Approximately 500,000 cells from the CD117 positive fraction were cultured per well in a 24-well culture plate in 500μL of X-VIVO 15 cell culture media (Fisher Scientific, #BW04-418Q) with 50 ng/mL murine stem cell factor (SCF) (PeproTech, #250-03) and 20 ng/mL murine thrombopoietin (TPO) (PeproTech, #315-14). Fluticasone propionate (FLU) (Sigma-Aldrich, F9428) was cultured at a concentration of 3nM unless otherwise specified. FLU and dexamethasone (abcam, ab120743) were reconstituted in DMSO. To block glucocorticoid receptor (GR), 1μM Mifepristone (RU486) (Sigma-Aldrich, #M8046) was simultaneously cultured with cells and FLU. Poly vinyl alcohol (PVA) (Sigma-Aldrich, #P8136) culture media was taken from (Wilkinson *et al*, 2019). All cells were cultured for 16 h in 37°C and 5% CO_2. After incubation, cells were analyzed using flow cytometry with antibodies listed in Appendix Table S1 ("Antibodies Table") in FACS buffer.

Chemotaxis Assay

Transwell migration assays were performed in 24 well-6.5mm diameter and 5.0µm pore size insert tissue culture treated transwell plates (Corning, #3421). After HSC cell culture with FLU or Vehicle culture, cells from each condition were washed and resuspended in 100µL X-VIVO 15 plus 50 ng/mL SCF and 20 ng/mL TPO culture media lacking FLU. Cells were transferred into transwell porous inserts resting in a bottom well containing 650 µL prewarmed X-VIVO 15/SCF/TPO media and 50 ng/mL mouse recombinant SDF-1 alpha (CXCL12) (Stem Cell Technologies, #78121). Transwells were cultured for 5 h in 37ºC and 5% CO_2. To block CXCR4, cells were pre-treated with 10 µg/mL AMD3100 (Sigma-Aldrich, 239820) for 30 minutes in 500 µL of X-VIVO 15/SCF/TPO media lacking FLU. After chemotactic assay incubation, cells were analyzed using Flow cytometry using antibodies listed in Supplementary Information Table S1 ("Antibodies Table") in FACS buffer. Cells from the top insert and bottom well were stained separately and counted using Countbright beads (Thermofisher, #C36950) to determine the number of migrated HSCs.

Cell Sorting

BM was harvested from donor mice by crushing leg bones in ice-cold FACS buffer followed by red blood cell lysis by ACK lysis buffer and filtration through a 70 µ mesh to remove debris. BM was Kit-enriched using the Milyenyi AutoMACs Pro (Miltenyi Biotec, Somerville, MA). Cells were stained with antibodies listed in Supporting Information Table S1 ("Antibodies Table") in FACS buffer. Cells were then fluorescence-activated cell sorted using a BD FACS-Aria II (Becton Dickinson, Franklin Lakes, NJ) into a 96-well U bottom plate containing 100 µL of X-VIVO 15/SCF/TPO culture media with or without FLU. Donor TM5 (CFP+) and mTmG (tdTomato+) mouse-derived cells were sorted into separate wells and TM5 was further split into FLU or vehicle containing wells. mTmG cells were all sorted into media only containing vehicle. Each well contained $1x10^4$ KLS (Ter119- CD27+ c-kit+ Sca1+) gated cells.

T cell Activation Assay

A tissue culture-treated flat bottom 96-well plate (Costar) was incubated with 30 µg/ml anti-hamster IgG (Vector Laboratories) for 1 hour at 37°C. Non-stimulated condition wells were

incubated with only PBS. Wells were then washed with PBS and then incubated with 5 μg/ml anti-mouse CD3e (eBiosciences) for 2 hours at 37°C. Non-stimulated condition wells were incubated with only PBS. Freshly harvested splenocytes (1.2 x 10^6) from CFP+ C57BL/6 (TM5) mice, pre-treated in triplicate with 3nM FLU or DMSO Vehicle, were stained with 5 μM CFSE (Biolegend) at a concentration of 10×10^6 cells/mL for 20 minutes at room temperature in the dark. Splenocytes were washed then split into two samples of 0.6 x 10^6 cells in 200μL complete RPMI-1640 (C-RPMI) stimulated and unstimulated condition with and without 2 μg/ml anti-mouse CD28 (eBiosciences) respectively and plated in stimulated (with eCD3), or non-stimulated (without eCD3) wells and incubated for 4 days at 37°C and 5%CO_2. Complete RPMI (C-RPMI) media was composed of RPMI 1640 with L-Glutamine, 25 mM HEPES, 0.11 mM 2-Mercaptoethanol, 1X MEM Non-Essential Amino Acids, 1 mM sodium pyruvate, and 10% heat inactivated (HI) FBS all from Gibco and 1X Penicillin/Streptomycin from Corning. After incubation, cells were analyzed using flow cytometry with antibodies listed in Appendix Table S1 in heat-inactivated (HI) FACS buffer.

Mixed Lymphocyte Reaction

Freshly harvested splenocytes (3×10^5) from CFP+ (H-2^b) mice were pre-treated in triplicate with 3nM FLU or DMSO Vehicle then used for responder cells. Responder cells were co-cultured (1:1) with (3×10^5) irradiated (2500cGy) allogeneic Balb/c or syngeneic C57BL/6 stimulator splenocytes for 4 days in 96-well round bottom plate with 200uL of C-RPMI. C-RPMI was used throughout this experiment. After incubation, cells were analyzed using flow cytometry with antibodies listed in Appendix Table S1 in HI FACS buffer.

Statistical Analysis

Statistical analysis was performed with GraphPad Prism 5 software (La Jolla, CA). Specific statistical tests are described for each experiment in the Figure Legends.

Appendix Table S1. Antibodies

Antigen	Clone	Conjugate	Source	Catalogue #
TER119	TER119	PE/Cy5	Biolegend	116210
	TER119	BV421	Biolegend	116233
SCA1 (Ly-6A/E)	E13-161.7	PE/Cy7	eBioscience	122514
	E13-161.7	PE	Biolegend	122507
KIT (CD117)	ACK2	APC	Biolegend	135107
	2B8	APC-eFluor 780	eBioscience	47-1171-82
	2B8	BV421	Biolegend	105828
CD27	LG.7F9	eFluor 780	eBioscience	47-0271-82
	LG.7F9	APC	eBioscience	17-0271-82
CD11A	M17/4	PE/Cy7	eBioscience	25-0111-30
	M17/4	Biotin	Biolegend	101103
	M17/4	APC	Biolegend	101119
	M17/4	PE	Biolegend	101107
	M17/4	FITC	Biolegend	101106
	M17/4	Alexa Fluor 488	Biolegend	101111
EPCR (CD201)	eBio1560	PerCP-eFluor 710	eBioscience	46-2012-82
	eBio1560	APC	eBioscience	17-2012-82
GR1 (Ly-6G/Ly-6C)	RB6-8C5	Alexa Fluor 700	eBioscience	108422
MAC1 (CD11b)	M1/70	APC	Biolegend	101212
	M1/70	FITC	Biolegend	101205
CD19	6D5	APC	Biolegend	115512
	eBio1D3	PerCP-Cy5.5	eBioscience	45-0193-82
	6D5	BV421	Biolegend	115537
CD45	30-F11	APC/Cy7	Biolegend	103116
	30-F11	Alexa Fluor 700	Biolegend	103128
CD45.2	104	FITC	Biolegend	109806
CD45.1	A20	PE/Cy7	Biolegend	110729
CD3ε	17A2	PerCP-eFluor 710	eBioscience	46-0032-82
	17A2	PE/Cy7	Biolegend	100220
CD150 (SlamF1)	TC15-12F12.2	Brilliant Violet 650	Biolegend	115931
CD4	RM4-5	PE/Cy7	Biolegend	100527
CD8a	53-6.7	APC/Cy7	Biolegend	100714
CD93 (AA4.1)	AA4.1	APC	eBioscience	17-5893-81
IgD	11-26c.2a	Alexa Fluor 700	Biolegend	405729
IgM	RMM-1	APC/Cy7	Biolegend	406515
B220	RA3-6B2	BV605	Biolegend	103243
Cxcr4	L276F12	PE	Biolegend	146506
	L276F12	APC	Biologend	146508
**Secondary antibodies**				
		Qdot 655-Streptavidin	Life Technologies	Q10121MP
		Qdot 605-Streptavidin	Life Technologies	Q10103MP
		eFluor710-Streptavidin	eBioscience	49-4317-80

Appendix References

Wilkinson AC, Ishida R, Kikuchi M, *et al*. (2019) Long-term ex vivo hematopoietic stem cell expansion affords nonconditioned transplantation. *Nature*. 571(7763).
