# Supplementary material for: Graft conditioning with fluticasone propionate reduces graft‐versus‐host disease upon allogeneic hematopoietic cell transplantation in mice
Source: EMBO Mol Med. 2023 Aug 4;15(9):e17748. doi: 10.15252/emmm.202317748 (PMC10493574; doi:10.15252/emmm.202317748)
Supplement: Supplementary file 4 — Source Data for Figure 1 [file EMMM-15-e17748-s003.zip › Figure 1/1B/README_fig1B.rtf]

FIGURE 1BHow to interpret:The values represent the mean fluorescent intensity (MFI) of CXCR4 on hematopoietic stem cells (HSCs) after being treated in vitro with either vehicle, Flonase (FLU), or FLU plus a glucocorticoid receptor antagonist (RU486)
