# Supplementary material for: Graft conditioning with fluticasone propionate reduces graft‐versus‐host disease upon allogeneic hematopoietic cell transplantation in mice
Source: EMBO Mol Med. 2023 Aug 4;15(9):e17748. doi: 10.15252/emmm.202317748 (PMC10493574; doi:10.15252/emmm.202317748)
Supplement: Supplementary file 4 — Source Data for Figure 1 [file EMMM-15-e17748-s003.zip › Figure 1/1D/README_fig1D.rtf]

FIGURE 1DiiHow to interpret:Flow cytometry analysis of donor HSC chimerism in the BM 12 weeks post transplant in both recipient groups. Shown is the percentage of CFP+ donor HSCs in the Flonase group relative to the percentage of FP+ donor HSCs in the vehicle group.
