# Supplementary material for: Graft conditioning with fluticasone propionate reduces graft‐versus‐host disease upon allogeneic hematopoietic cell transplantation in mice
Source: EMBO Mol Med. 2023 Aug 4;15(9):e17748. doi: 10.15252/emmm.202317748 (PMC10493574; doi:10.15252/emmm.202317748)
Supplement: Supplementary file 4 — Source Data for Figure 1 [file EMMM-15-e17748-s003.zip › Figure 1/1C/README_fig1C.rtf]

FIGURE 1C	How to interpret:This graph represents the percent migration of hematopoietic stem cels (HSCs) in a trans well assay. There are 5 conditions1. No SDF-1a (no chemical gradient control)2. Vehicle treated HSCs3. Vehicle + AMD3100 (a CXCR4 antagonist) treated HSCs4. Flonase (FLU) treated HSCs5. FLU + AMD3100 treated HSCs
