# Supplementary material for: Graft conditioning with fluticasone propionate reduces graft‐versus‐host disease upon allogeneic hematopoietic cell transplantation in mice
Source: EMBO Mol Med. 2023 Aug 4;15(9):e17748. doi: 10.15252/emmm.202317748 (PMC10493574; doi:10.15252/emmm.202317748)
Supplement: Supplementary file 5 — Source Data for Figure 2 [file EMMM-15-e17748-s001.zip › Figure 2/2F/README_fig2F.rtf]

FIGURE 2FHow to interpret figure 2FThese are clinical scores for the H&E stained tissue sections. Each cell with a value is the clinical score for that corresponding individual mouse. Each Column is the condition and tissue combination. Abbreviations: Syngeneic (Syn), Vehicle (Veh), Flonase (FLU), Small Intestine (SI).The values were determined by a trained pathologist from the UCI Chao Family Comprehensive Center and was blinded to the identity of the mouse. How the values were determined: The degree of histopathology observed was scored using a semi-quantitative scoring system (0-3 where 0= normal, 1= mild, 2=moderate, and 3=severe) for the following features as follows: Small intestine and colon: Lamina propria inflammation, crypt atrophy, and crypt epithelial apoptosis Highest possible score is a 9.
