# Supplementary material for: Graft conditioning with fluticasone propionate reduces graft‐versus‐host disease upon allogeneic hematopoietic cell transplantation in mice
Source: EMBO Mol Med. 2023 Aug 4;15(9):e17748. doi: 10.15252/emmm.202317748 (PMC10493574; doi:10.15252/emmm.202317748)
Supplement: Supplementary file 5 — Source Data for Figure 2 [file EMMM-15-e17748-s001.zip › Figure 2/2C/README_fig2C.rtf]

FIGURE 2CHow to interpret Figure 2C “survival curve”The leftmost column refers to days post-transplant (p.t.)The remaining columns are the conditionsA “0” means the animal did not die on that dayA “1” means the animal did die on that day
