# Supplementary material for: Graft conditioning with fluticasone propionate reduces graft‐versus‐host disease upon allogeneic hematopoietic cell transplantation in mice
Source: EMBO Mol Med. 2023 Aug 4;15(9):e17748. doi: 10.15252/emmm.202317748 (PMC10493574; doi:10.15252/emmm.202317748)
Supplement: Supplementary file 5 — Source Data for Figure 2 [file EMMM-15-e17748-s001.zip › Figure 2/2D/README_fig2D.rtf]

FIGURE 2DHow to interpret Figure 2DThe left most column refers to days post-transplant (p.t.)The remaining columns are the individual mice within each condition I.e. V1 refers to mouse 1 of the vehicle cohort.Each value refers to that animal’s clinical score for that dayWhen an animal reaches a score of 5, they are euthanized and retain a score of 5 for the remainder of the study. If the mouse dies of graft versus host disease they also retain a 5 for the remainder of the study.
