# Supplementary material for: Graft conditioning with fluticasone propionate reduces graft‐versus‐host disease upon allogeneic hematopoietic cell transplantation in mice
Source: EMBO Mol Med. 2023 Aug 4;15(9):e17748. doi: 10.15252/emmm.202317748 (PMC10493574; doi:10.15252/emmm.202317748)
Supplement: Supplementary file 5 — Source Data for Figure 2 [file EMMM-15-e17748-s001.zip › Figure 2/2E/README_fig2E.rtf]

Figure 2ERepresentative images of small intestine (SI) or colon from mice either receiving vehicle or Flonase treated cells.Image is 2X. Red box is the region displayed in manuscript.Scale bar is 500 microns.
