# Supplementary figures and images for: Graft conditioning with fluticasone propionate reduces graft‐versus‐host disease upon allogeneic hematopoietic cell transplantation in mice
Source: EMBO Mol Med. 2023 Aug 4;15(9):e17748. doi: 10.15252/emmm.202317748 (PMC10493574; doi:10.15252/emmm.202317748)

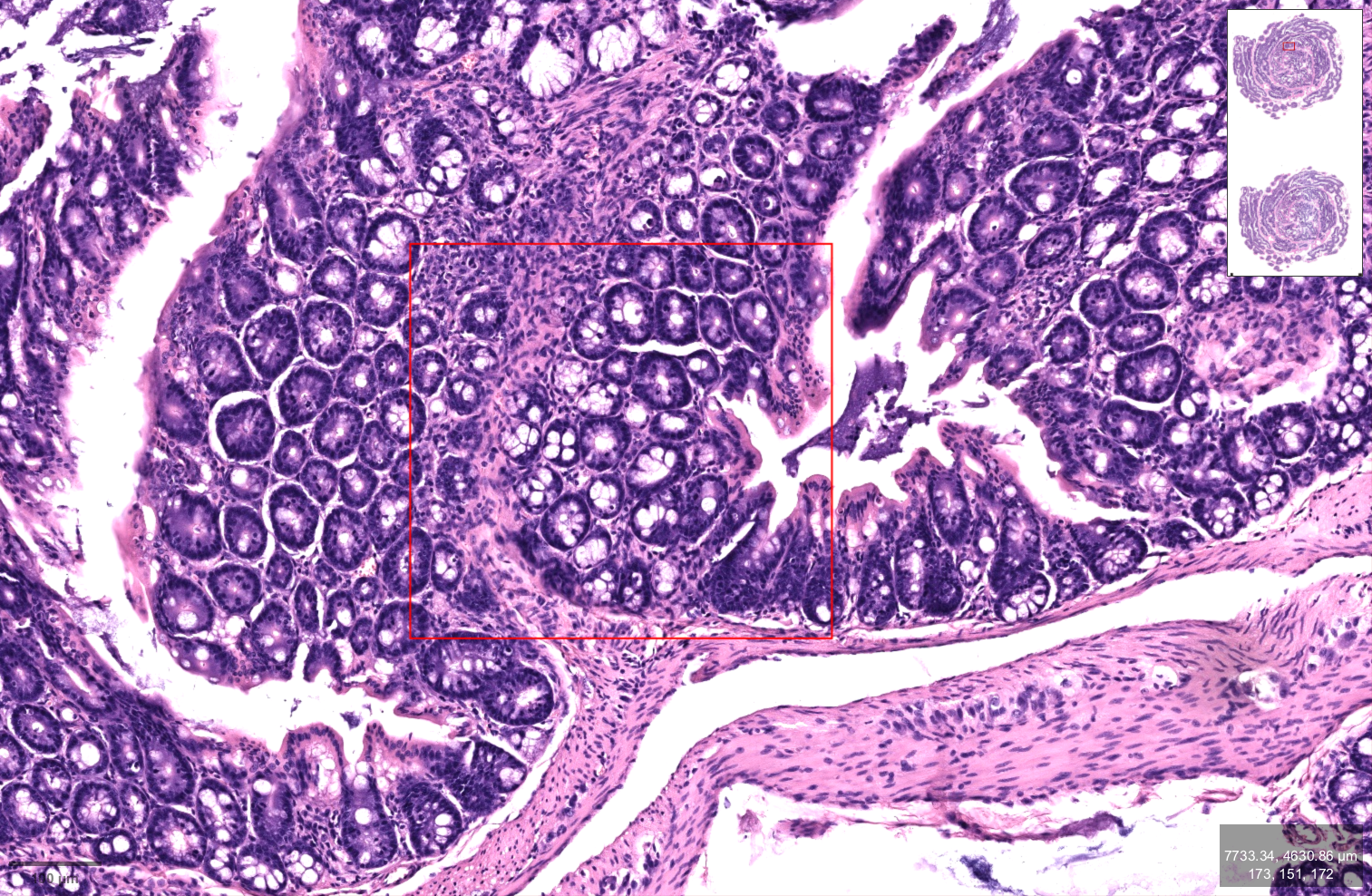

Supplement: Supplementary file 5 — Source Data for Figure 2 [file EMMM-15-e17748-s001.zip › Figure 2/2E/FLU colon 10x.tif]

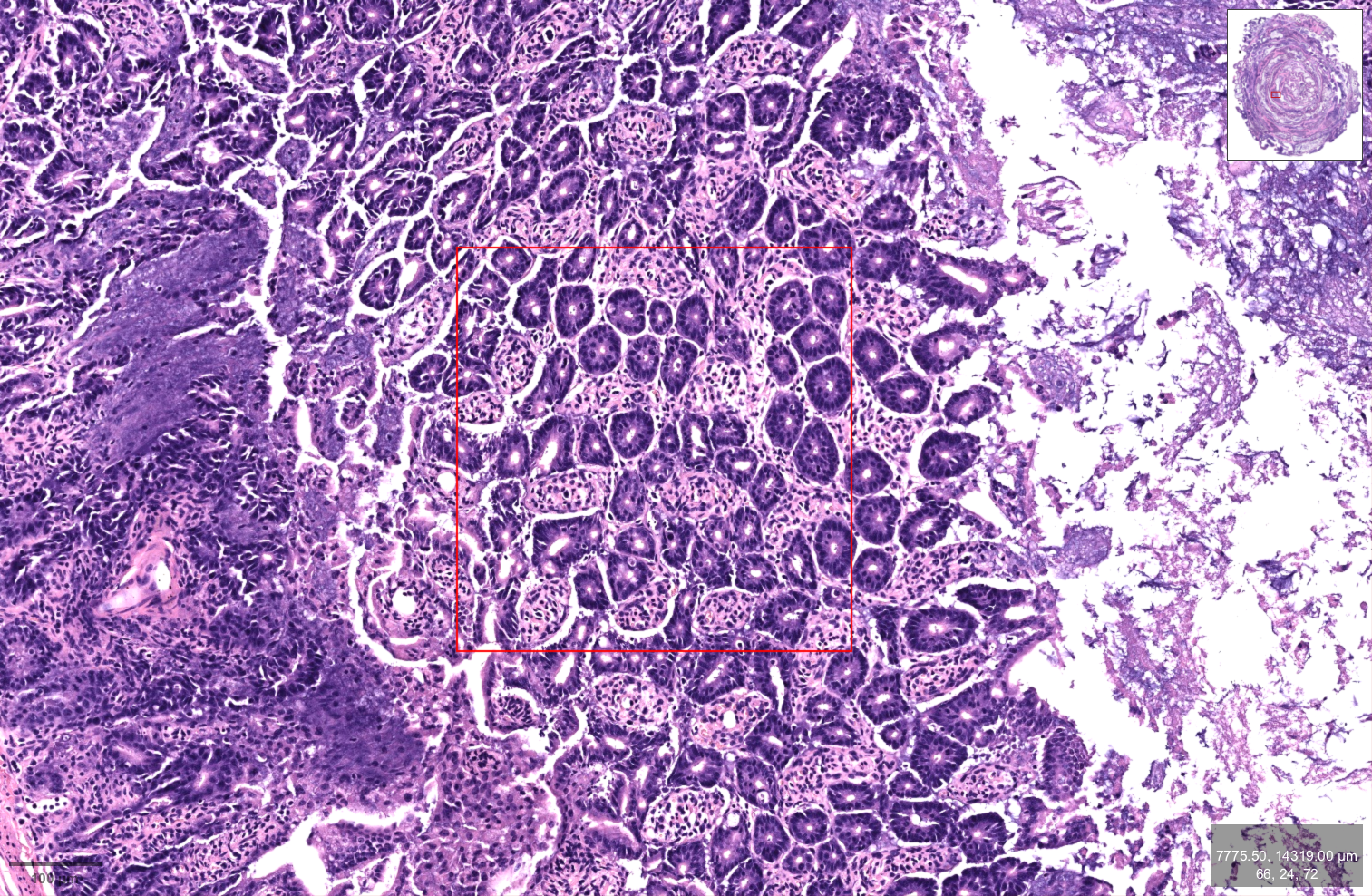

Supplement: Supplementary file 5 — Source Data for Figure 2 [file EMMM-15-e17748-s001.zip › Figure 2/2E/Veh SI 10x.tif]

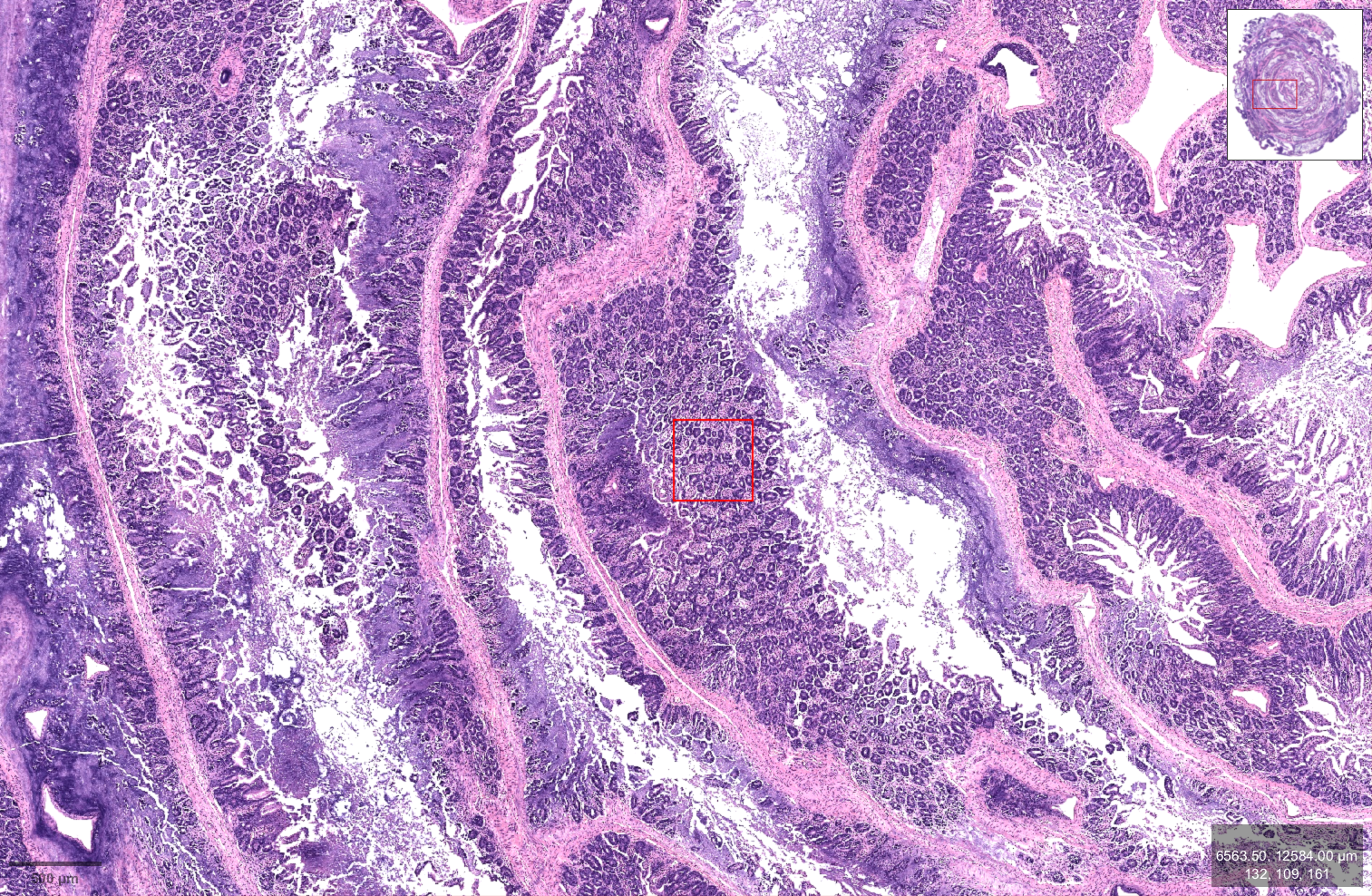

Supplement: Supplementary file 5 — Source Data for Figure 2 [file EMMM-15-e17748-s001.zip › Figure 2/2E/Veh SI 2x.tif]

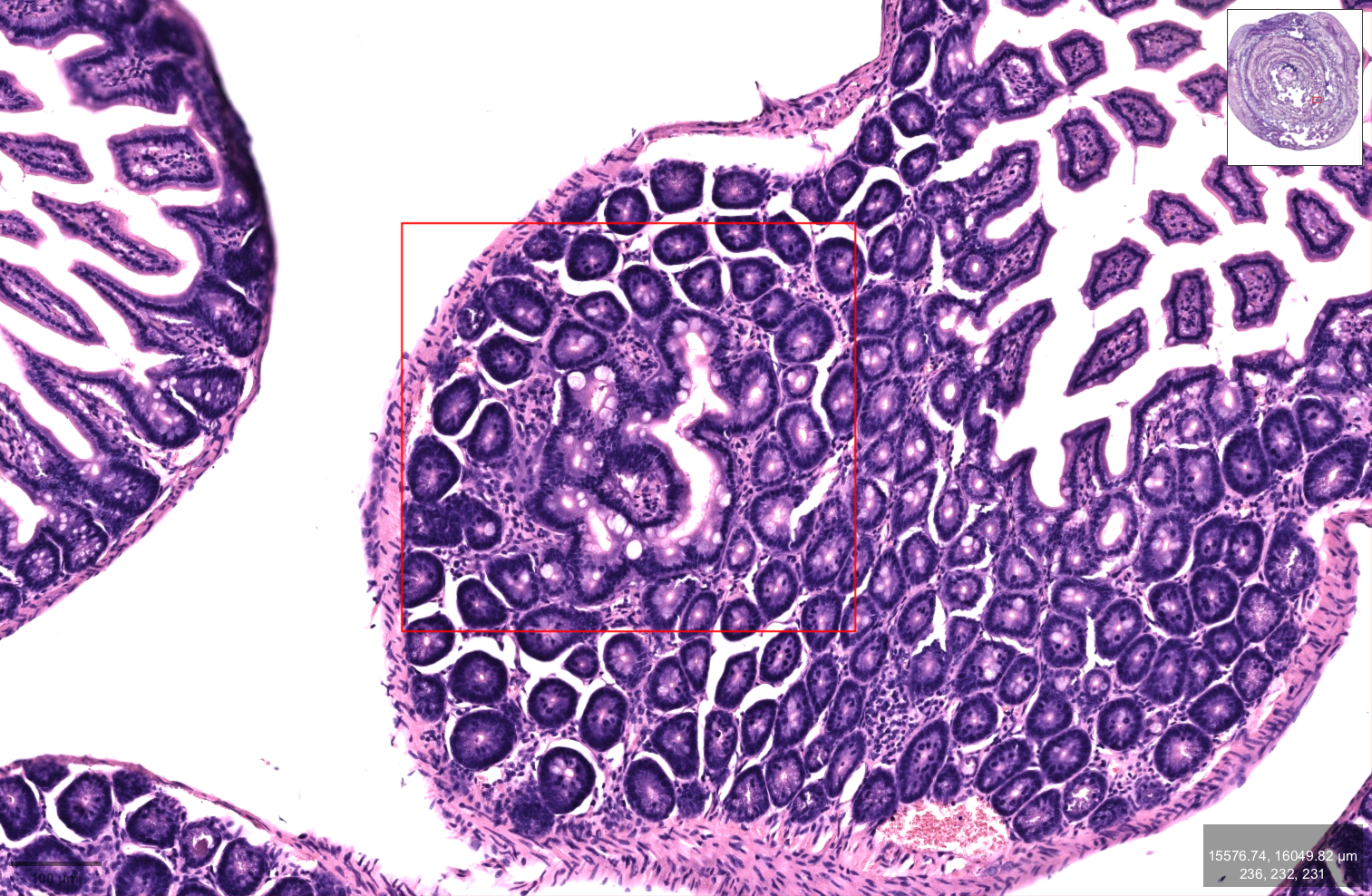

Supplement: Supplementary file 5 — Source Data for Figure 2 [file EMMM-15-e17748-s001.zip › Figure 2/2E/FLU SI 10x.tif]

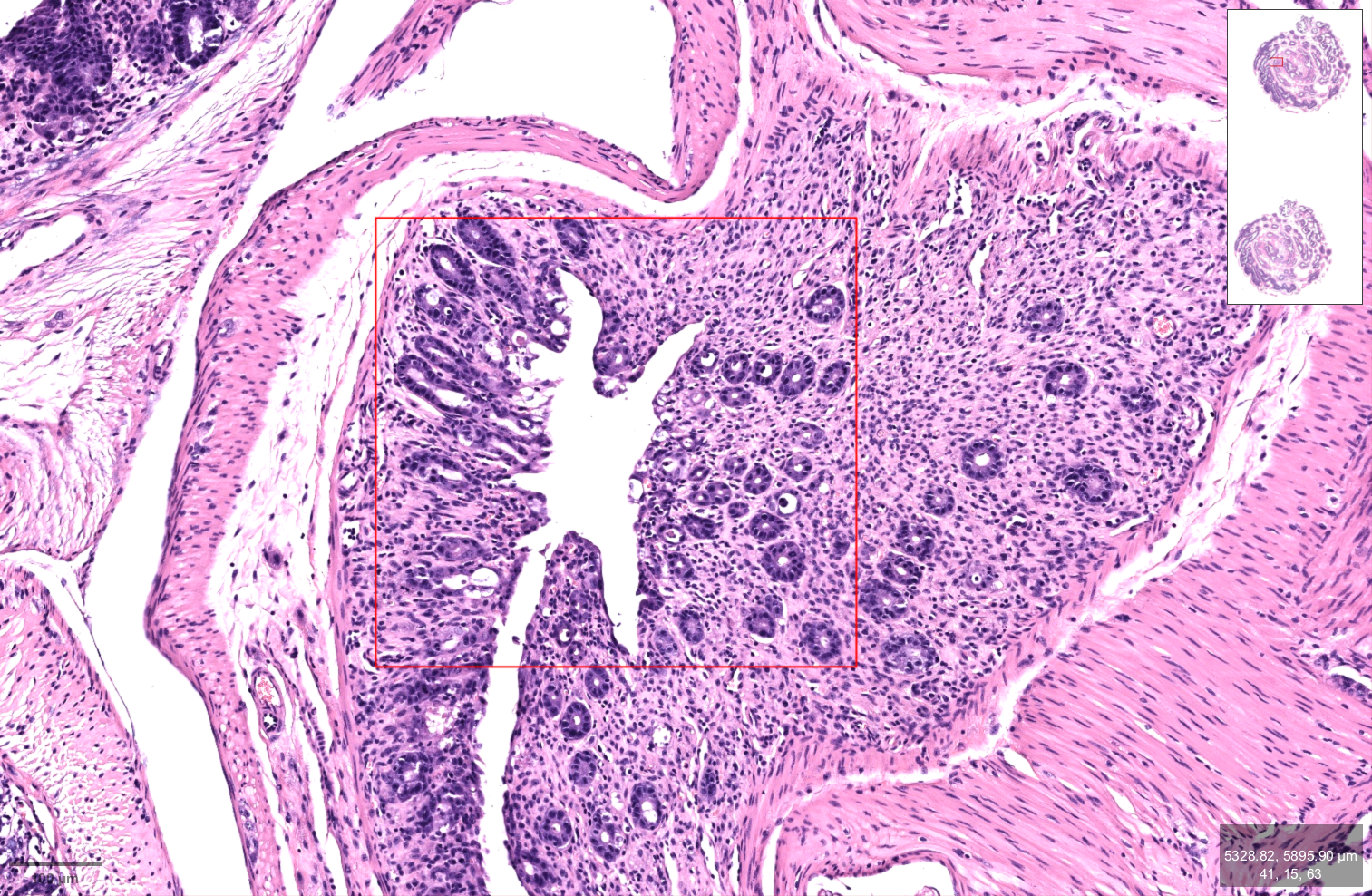

Supplement: Supplementary file 5 — Source Data for Figure 2 [file EMMM-15-e17748-s001.zip › Figure 2/2E/Veh colon 10x.tif]

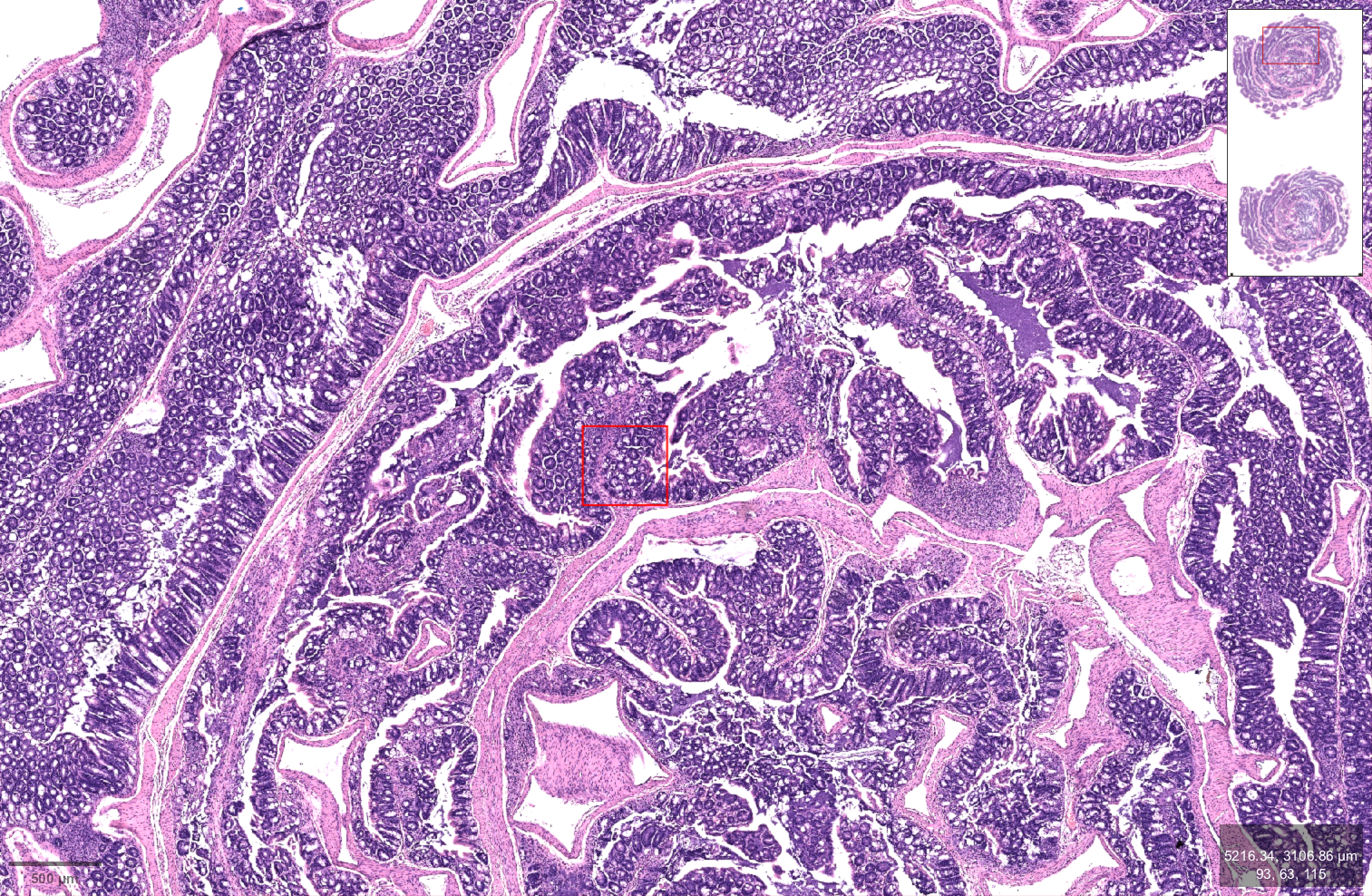

Supplement: Supplementary file 5 — Source Data for Figure 2 [file EMMM-15-e17748-s001.zip › Figure 2/2E/FLU colon 2x.tif]

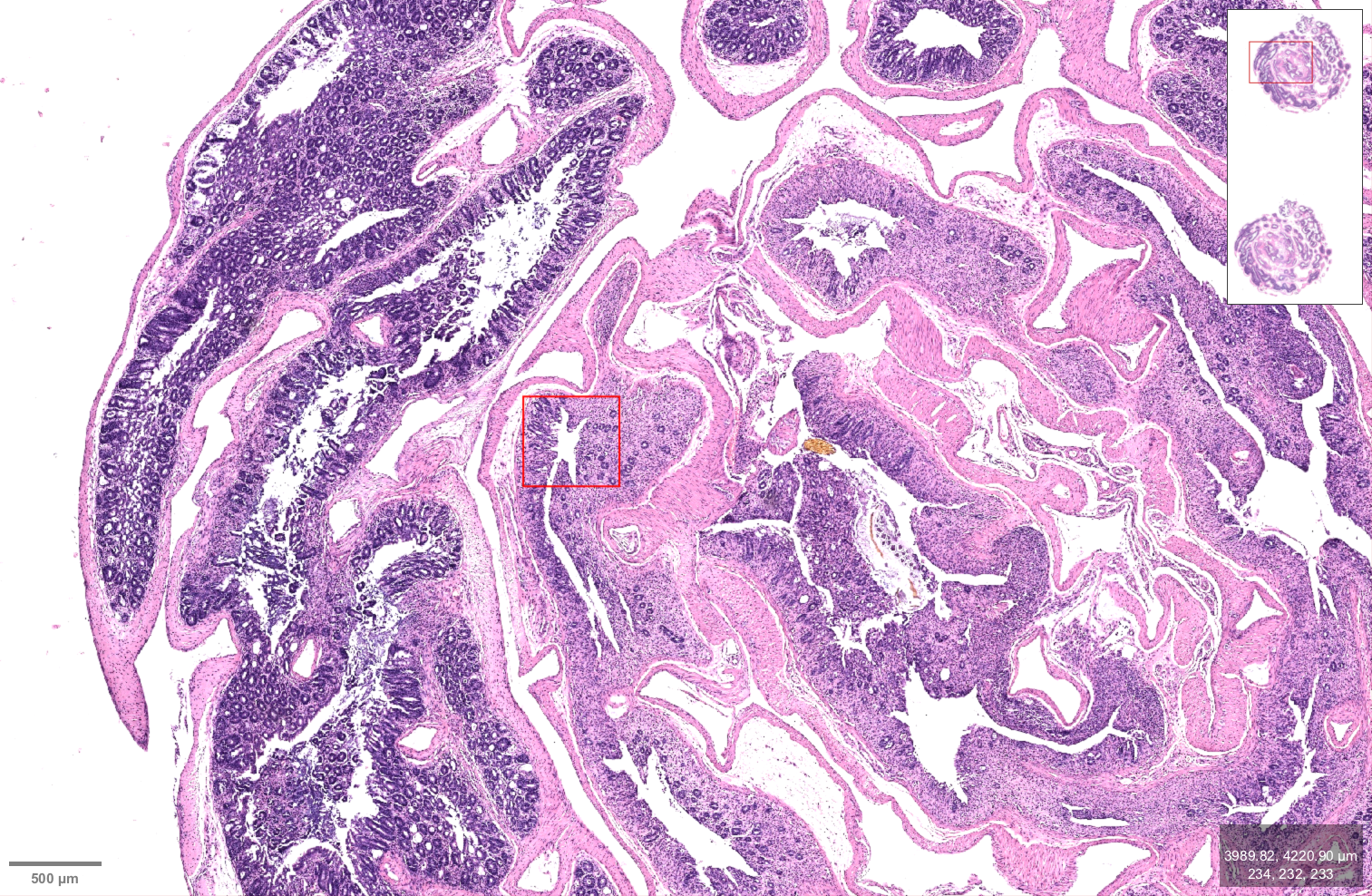

Supplement: Supplementary file 5 — Source Data for Figure 2 [file EMMM-15-e17748-s001.zip › Figure 2/2E/Veh colon 2x.tif]

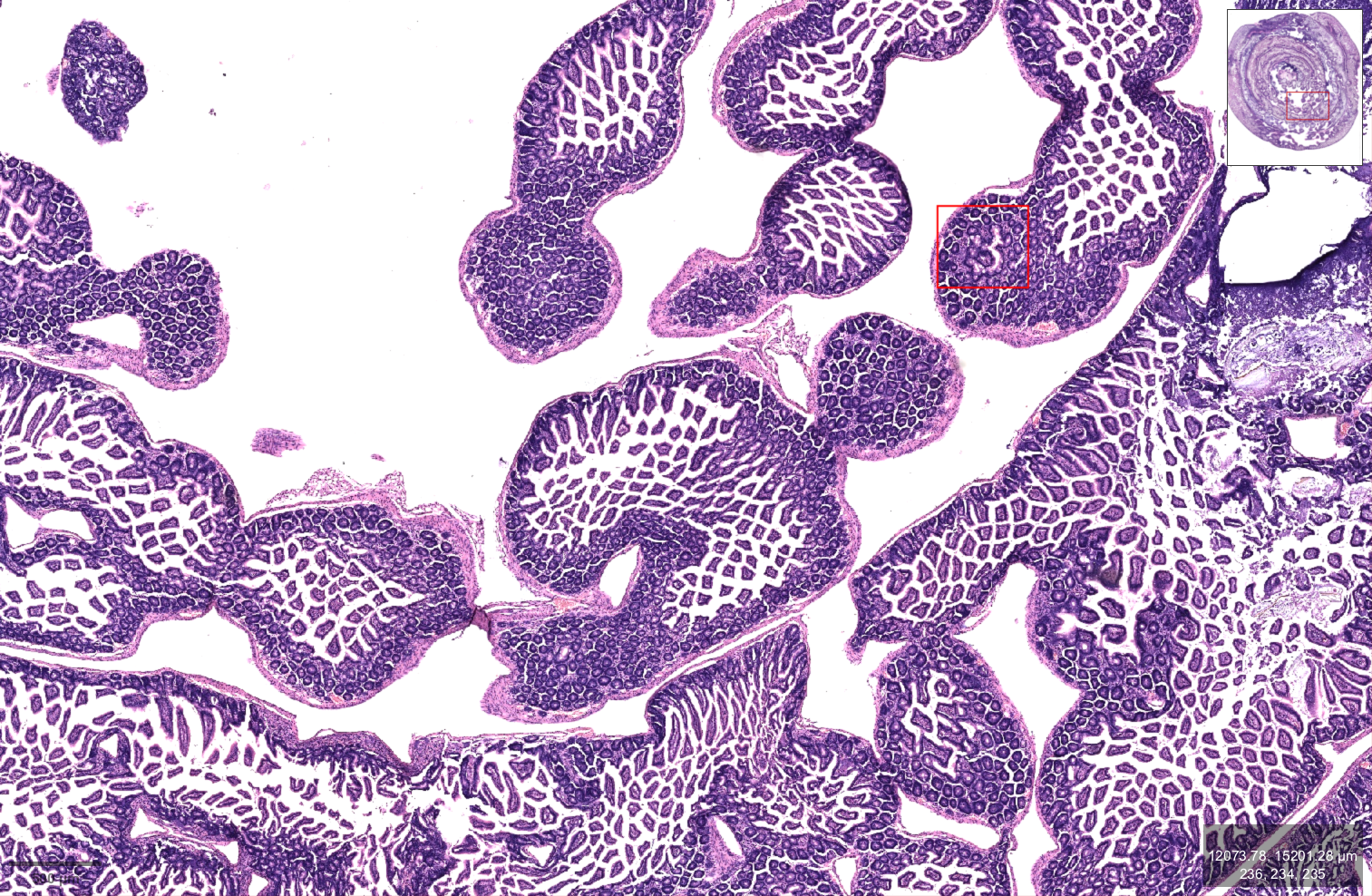

Supplement: Supplementary file 5 — Source Data for Figure 2 [file EMMM-15-e17748-s001.zip › Figure 2/2E/FLU SI 2x.tif]
