# Supplementary material for: Graft conditioning with fluticasone propionate reduces graft‐versus‐host disease upon allogeneic hematopoietic cell transplantation in mice
Source: EMBO Mol Med. 2023 Aug 4;15(9):e17748. doi: 10.15252/emmm.202317748 (PMC10493574; doi:10.15252/emmm.202317748)
Supplement: Supplementary file 6 — Source Data for Figure 3 [file EMMM-15-e17748-s004.zip › Figure 3/3E/README_fig3E.rtf]

How to interpret Figure 3Eii and 3EiiiEii is gated on live, single, CD4+ OR CD8+ cells, then on IFNg+ cells.Each cell represents one recipient from the experiment, either receiving vehicle or Flonase (FLU) treated cells.Eiii is gated on live single CD4+ cells and then on either IL-4+ or IL-17+ cellsEach cell represents one recipient
