# Supplementary material for: Graft conditioning with fluticasone propionate reduces graft‐versus‐host disease upon allogeneic hematopoietic cell transplantation in mice
Source: EMBO Mol Med. 2023 Aug 4;15(9):e17748. doi: 10.15252/emmm.202317748 (PMC10493574; doi:10.15252/emmm.202317748)
Supplement: Supplementary file 6 — Source Data for Figure 3 [file EMMM-15-e17748-s004.zip › Figure 3/3C/README_figure3C.rtf]

FIGURE 3CHow to interpret figure 3CThis looks at the percent of CD3+ T cells in total CD45+ cells in various tissues post transplant.Veh = mice receiving vehicle treated cellsFLU= mice receiving Flonase treated cellsSyn = syngeneic control (B6 to B6 transplant)
