# Supplementary material for: Graft conditioning with fluticasone propionate reduces graft‐versus‐host disease upon allogeneic hematopoietic cell transplantation in mice
Source: EMBO Mol Med. 2023 Aug 4;15(9):e17748. doi: 10.15252/emmm.202317748 (PMC10493574; doi:10.15252/emmm.202317748)
Supplement: Supplementary file 6 — Source Data for Figure 3 [file EMMM-15-e17748-s004.zip › Figure 3/3D/README_fig3D.rtf]

FIGURE 3Dii and FIGURE 3DiiiDii: %CD69+ done CD4+ T cells post transplant (gated on live single cells)Each cell represents an individual animalAbbreviationsVehicle (Veh)Flonase (FLU)Syngeneic (Syn)Diii: mean fluorescent intensity (MFI) of CD44 on donor CD4+ T cells post transplant.Each cell represents an individual animal
