# Supplementary material for: Graft conditioning with fluticasone propionate reduces graft‐versus‐host disease upon allogeneic hematopoietic cell transplantation in mice
Source: EMBO Mol Med. 2023 Aug 4;15(9):e17748. doi: 10.15252/emmm.202317748 (PMC10493574; doi:10.15252/emmm.202317748)
Supplement: Supplementary file 6 — Source Data for Figure 3 [file EMMM-15-e17748-s004.zip › Figure 3/3A/README_fig3A.rtf]

FIGURE 3AHow to interpret figure 3AValue represents the %live cells by annexing negative/ propidium iodide negativeEach cell represents an individual mouseAbbreviationsVehicle (Veh)Flonase (FLU)
