# Supplementary material for: Graft conditioning with fluticasone propionate reduces graft‐versus‐host disease upon allogeneic hematopoietic cell transplantation in mice
Source: EMBO Mol Med. 2023 Aug 4;15(9):e17748. doi: 10.15252/emmm.202317748 (PMC10493574; doi:10.15252/emmm.202317748)
Supplement: Supplementary file 7 — Source Data for Figure 4 [file EMMM-15-e17748-s002.zip › Figure 4/4A/README_fig4A.rtf]

FIGURE 4Aii and 4AiiiHow to interpret:Next to each sub category of either Flonase stimulated, Vehicle Stimulated or Vehicle unstimulated, there is a column labeled “animal ID”, to the right of the animal ID are 3 values which are technical replicates, however, each animal ID is an individual animal which are biological replicates. Spleens from mice in the vehicle cohort were split into stimulated or unstimulated conditions, so VS1 and VU1 come from the same spleen. Figure 4Aii looks at the percent of CD25+ cells in either CD4+ cell population or CD8+, both of which are gated on live single CD3+ cell populations.Figure 4Aiii is the same except looking at PD-1+ cell percentages.
