# Supplementary material for: Graft conditioning with fluticasone propionate reduces graft‐versus‐host disease upon allogeneic hematopoietic cell transplantation in mice
Source: EMBO Mol Med. 2023 Aug 4;15(9):e17748. doi: 10.15252/emmm.202317748 (PMC10493574; doi:10.15252/emmm.202317748)
Supplement: Supplementary file 8 — Source Data for Figure 5 [file EMMM-15-e17748-s007.zip › Figure 5/5C/README_fig5C.rtf]

Figure 5CHow to interpret figure 5CThe left most column refers to days post-transplant (p.t.)Each value refers to that animal’s clinical score for that dayWhen an animal reaches a score of 5, they are euthanized and retain a score of 5 for the remainder of the study. If the mouse dies of graft versus host disease they also retain a 5 for the remainder of the study.
