# Supplementary material for: Graft conditioning with fluticasone propionate reduces graft‐versus‐host disease upon allogeneic hematopoietic cell transplantation in mice
Source: EMBO Mol Med. 2023 Aug 4;15(9):e17748. doi: 10.15252/emmm.202317748 (PMC10493574; doi:10.15252/emmm.202317748)
Supplement: Supplementary file 8 — Source Data for Figure 5 [file EMMM-15-e17748-s007.zip › Figure 5/5D/README_fig5D.rtf]

Figure 5DPercent of donor CD45+ chimerism in secondary recipients out of total CD45+ cells.Veh is mice receiving vehicle treated cellsFLU is mice receiving Flonase treated cellsSyn is a syngeneic transplant (B6—>B6)
