# Supplementary material for: Graft conditioning with fluticasone propionate reduces graft‐versus‐host disease upon allogeneic hematopoietic cell transplantation in mice
Source: EMBO Mol Med. 2023 Aug 4;15(9):e17748. doi: 10.15252/emmm.202317748 (PMC10493574; doi:10.15252/emmm.202317748)
Supplement: Supplementary file 8 — Source Data for Figure 5 [file EMMM-15-e17748-s007.zip › Figure 5/5B/README_fig5B.rtf]

Figure 5BHow to interpret figure 5BColumn A refers to the iD of each animalColumn B is the number of days post transplantationColumn C is mice receiving vehicle treated cells Column D is mice receiving Flonase treated cells Column E is syngeneic control(0 means alive, 1 means death event at time point)
