# Supplementary material for: Graft conditioning with fluticasone propionate reduces graft‐versus‐host disease upon allogeneic hematopoietic cell transplantation in mice
Source: EMBO Mol Med. 2023 Aug 4;15(9):e17748. doi: 10.15252/emmm.202317748 (PMC10493574; doi:10.15252/emmm.202317748)
Supplement: Supplementary file 9 — Source Data for Figure 6 [file EMMM-15-e17748-s010.zip › Figure 6/6A/README_fig6A.rtf]

FIGURE 6AHow to interpret:This data looks at the viability of T cell subsets by PI and annexinV. There are 3 biological replicates and each biological replicate has 3 technical replicates. Each cell represents the %viable cells. Veh is vehicle treated splenocytesFLU is Flonase treated splenocytes
