# Supplementary material for: Graft conditioning with fluticasone propionate reduces graft‐versus‐host disease upon allogeneic hematopoietic cell transplantation in mice
Source: EMBO Mol Med. 2023 Aug 4;15(9):e17748. doi: 10.15252/emmm.202317748 (PMC10493574; doi:10.15252/emmm.202317748)
Supplement: Supplementary file 9 — Source Data for Figure 6 [file EMMM-15-e17748-s010.zip › Figure 6/6B/README_fig6B.rtf]

FIGURE 6BHow to interpret:This data looks at the percent of FoxP3+ cells in vitro after being treated with either vehicle or FlonaseThere are 3 biological replicates and each biological replicate has 3 technical replicates.
