# Supplementary material for: Graft conditioning with fluticasone propionate reduces graft‐versus‐host disease upon allogeneic hematopoietic cell transplantation in mice
Source: EMBO Mol Med. 2023 Aug 4;15(9):e17748. doi: 10.15252/emmm.202317748 (PMC10493574; doi:10.15252/emmm.202317748)
Supplement: Supplementary file 9 — Source Data for Figure 6 [file EMMM-15-e17748-s010.zip › Figure 6/6C/README_fig6C.rtf]

FIGURE 6CiiiHow to interpret:This data represents the percent of FoxP3+ from donor CD4+ T cells from varying tissues harvested 7-9 days post transplant in 2 groups of recipients. Recipients either received vehicle or Flonase treated spleen cells. There are 5 biological replicates.
