# Supplementary material for: Graft conditioning with fluticasone propionate reduces graft‐versus‐host disease upon allogeneic hematopoietic cell transplantation in mice
Source: EMBO Mol Med. 2023 Aug 4;15(9):e17748. doi: 10.15252/emmm.202317748 (PMC10493574; doi:10.15252/emmm.202317748)
Supplement: Supplementary file 10 [file EMMM-15-e17748-s006.pdf]

EMBO Molecular Medicine

Graft conditioning with fluticasone propionate reduces graft-versus-host disease upon allogeneic hematopoietic cell transplantation in mice

Erika Varady, Angel Ayala, Pauline Nguyen, Vanessa Scarfone, Alborz Karimzadeh, Cuiwen Zhou, Xiyu Chen, Scott Greilach, Craig Walsh, and Matthew Inlay
DOI: 10.15252/emmm.202317748

Corresponding author(s): Matthew Inlay (minlay@uci.edu)

Review Timeline:

Submission Date:	23rd Mar 23
Editorial Decision:	3rd May 23
Revision Received:	30th Jun 23
Accepted:	10th Jul 23

Editor: Kelly Anderson

Transaction Report:

(Note: With the exception of the correction of typographical or spelling errors that could be a source of ambiguity, letters and reports are not edited. Depending on transfer agreements, referee reports obtained elsewhere may or may not be included in this compilation. Referee reports are anonymous unless the Referee chooses to sign their reports.)

3rd May 2023

Dear Matt,

Congratulations, we've consulted with an expert in the field (see comments below) and have decided to move forward with the reviewed and revised version of your manuscript. However, we do ask that you please take care of the following editorial items and add this to a new point-by-point response:

1. Please upload the main figures as individual, high-resolution figure files and remove them from the manuscript text. Up to five EV figures (from the supplemental file) can also be uploaded as individual figure files and their legends should then be added to the manuscript, after the main figure legends.

2. Data availability: Please data and provide accession codes in this section according the online EMBO author guide (https://www.embopress.org/page/journal/14602075/authorguide#datadeposition). This can include Flow Cytometry results.

3. The funding information is missing in EJP, please add this.

4. Please remove the author contribution section from the manuscript.

5. Please review our new policy on conflict of interests on the EMBO author guide website and update the title of this section to: Disclosure and competing interests statement.

6. The paper explained: EMBO Molecular Medicine articles are accompanied by a summary of the articles to emphasize the major findings in the paper and their medical implications for the non-specialist reader. Please provide a draft summary of your article highlighting: the medical issue you are addressing, the results obtained and, their clinical impact. This may be edited to ensure that readers understand the significance and context of the research. Please refer to any of our published articles for an example.

7. For more information: There is space at the end of each article to list relevant web links for further consultation by our readers. Could you identify some relevant ones and provide such information as well? Some examples are patient associations, relevant databases, OMIM/proteins/genes links, author's websites, etc...

8. For references, please provide these in alphabetical order and only ten author names should be displayed. In cases where there are more than 10 authors, add et al. after the 10th author name.

9. Please add a table of contents to the appendix file and rename the figures with "Appendix Figure S1" etc. or Appendix Table S1". In figure call outs in the main text, please also update the renamed figures accordingly.

10. We require the publication of source data, particularly for electrophoretic gels and blots and graphs, with the aim of making primary data more accessible and transparent to the reader. Please provide this according to the email from Hannah Sonntag.

11. We do not allow "data not shown" Please remove this reference from p12 and p15.

12. Figure 3Ei is not called out, please add a reference to this in the correct order in the main manuscript.

13. Please remove the list of abbreviations.

14. Please move the materials and methods section after the Discussion section.

15. We include a synopsis of the paper (see http://emboj.embopress.org/). Please provide me with a general summary statement and 3-5 bullet points that capture the key findings of the paper.

16. We also need a summary figure for the synopsis. The size should be 550 wide by 200-440 high (pixels). You can also use something from the figures if that is easier.

17. Our publisher has also done their pre-publication check on your manuscript. I've attached the Data Edited Manuscript file. Please take a look at the word file and the comments regarding the figure legends and respond to the issues.

Thank you for the opportunity to consider your work for publication. I look forward to your revision.

All the best,
Kelly

Kelly M Anderson, PhD
Scientific Editor
EMBO Molecular Medicine

Expert Advisor Opinion:

I took a look at the paper, the reviewer comments, and author responses. I agree with reviewer #1, who raised very reasonable points initially, and whose comments were nicely addressed by the authors. I'm not sure why reviewer #2 is so dead-set on requiring GVL experiments. The reasons I think reviewer #2 is being unreasonable are: 1) The authors have already performed a great deal of work in the GVHD model, their studies are performed rigorously, and their findings are robust. 2) It is clear that performing the GVL studies would essentially double the size and scope of the experiments. There is no standard in the field of HSCT transplantation research that I am aware of that requires every manuscript to study GVHD and GVL for publication. 3) Allogeneic transplants are performed for non-neoplastic diseases all the time, so not sure why this would be a requirement. 4) The previous journal's position that this paper does not add new information of translational interest is not correct. GVHD remains a significant clinical problem, and therefore any new insights on potential ways to reduce the risk of this would be important. The fact that the strategy involves ex vivo treatment of the graft prior to transplant is a novel approach and could avoid toxicities/complications associated with chronic immunosuppression in the transplanted hosts. In short, the studies are novel, well-performed, and certainly of translational significance. I would strongly recommend accepting this paper - I don't think it needs to go through another round of reviews.

*** Instructions to submit your revised manuscript ***

*** PLEASE NOTE *** As part of the EMBO Publications transparent editorial process initiative (see our Editorial at https://www.embopress.org/doi/pdf/10.1002/emmm.201000094), EMBO Molecular Medicine will publish online a Review Process File to accompany accepted manuscripts.

In the event of acceptance, this file will be published in conjunction with your paper and will include the anonymous referee reports, your point-by-point response and all pertinent correspondence relating to the manuscript. If you do NOT want this file to be published, please inform the editorial office at contact@embomolmed.org.

To submit your manuscript, please follow this link:

https://embomolmed.msubmit.net/cgi-bin/main.plex

When submitting your revised manuscript, please include:

1) a .docx formatted version of the manuscript text (including Figure legends and tables)

2) Separate figure files*

3) supplemental information as Expanded View and/or Appendix. Please carefully check the authors guidelines for formatting Expanded view and Appendix figures and tables at
https://www.embopress.org/page/journal/17574684/authorguide#expandedview

4) a letter INCLUDING the reviewer's reports and your detailed responses to their comments (as Word file).

5) The paper explained: EMBO Molecular Medicine articles are accompanied by a summary of the articles to emphasize the major findings in the paper and their medical implications for the non-specialist reader. Please provide a draft summary of your article highlighting
- the medical issue you are addressing,
- the results obtained and
- their clinical impact.
This may be edited to ensure that readers understand the significance and context of the research.
Please refer to any of our published articles for an example.

6) For more information: There is space at the end of each article to list relevant web links for further consultation by our readers. Could you identify some relevant ones and provide such information as well? Some examples are patient associations, relevant databases, OMIM/proteins/genes links, author's websites, etc...

7) Author contributions: the contribution of every author must be detailed in a separate section.

8) EMBO Molecular Medicine now requires a complete author checklist (https://www.embopress.org/page/journal/17574684/authorguide) to be submitted with all revised manuscripts. Please use the checklist as guideline for the sort of information we need WITHIN the manuscript. The checklist should only be filled with page numbers were the information can be found. This is particularly important for animal reporting, antibody dilutions (missing) and exact values and n that should be indicted instead of a range.

9) Every published paper now includes a 'Synopsis' to further enhance discoverability. Synopses are displayed on the journal webpage and are freely accessible to all readers. They include a short stand first (maximum of 300 characters, including space) as well as 2-5 one sentence bullet points that summarise the paper. Please write the bullet points to summarise the key NEW findings. They should be designed to be complementary to the abstract - i.e. not repeat the same text. We encourage inclusion of key acronyms and quantitative information (maximum of 30 words / bullet point). Please use the passive voice. Please attach these in a separate file or send them by email, we will incorporate them accordingly.

You are also welcome to suggest a striking image or visual abstract to illustrate your article. If you do please provide a jpeg file 550 px-wide x 400-px high.

10) A Conflict of Interest statement should be provided in the main text

11) Please note that we now mandate that all corresponding authors list an ORCID digital identifier. This takes <90 seconds to complete. We encourage all authors to supply an ORCID identifier, which will be linked to their name for unambiguous name identification.

Currently, our records indicate that the ORCID for your account is 0000-0002-0451-2076.

Please click the link below to modify this ORCID:
Link Not Available

12) The system will prompt you to fill in your funding and payment information. This will allow Wiley to send you a quote for the article processing charge (APC) in case of acceptance. This quote takes into account any reduction or fee waivers that you may be eligible for. Authors do not need to pay any fees before their manuscript is accepted and transferred to our publisher.

*Additional important information regarding Figures

Each figure should be given in a separate file and should have the following resolution:
Graphs 800-1,200 DPI
Photos 400-800 DPI
Colour (only CMYK) 300-400 DPI"

Figures are not edited by the production team. All lettering should be the same size and style; figure panels should be indicated by capital letters (A, B, C etc). Gridlines are not allowed except for log plots. Figures should be numbered in the order of their appearance in the text with Arabic numerals. Each Figure must have a separate legend and a caption is needed for each panel.

*Additional important information regarding figures and illustrations can be found at https://bit.ly/EMBOPressFigurePreparationGuideline. See also figure legend preparation guidelines: https://www.embopress.org/page/journal/17574684/authorguide#figureformat

The system will prompt you to fill in your funding and payment information. This will allow Wiley to send you a quote for the article processing charge (APC) in case of acceptance. This quote takes into account any reduction or fee waivers that you may be eligible for. Authors do not need to pay any fees before their manuscript is accepted and transferred to our publisher.

Point-by-point response

Editor's comments are in black font. Author responses are in blue font. Changes made to the manuscript are indicated in blue font, although minor changes (like converting S5 to EV5) are not.

1. Please upload the main figures as individual, high-resolution figure files and remove them from the manuscript text. Up to five EV figures (from the supplemental file) can also be uploaded as individual figure files and their legends should then be added to the manuscript, after the main figure legends.

   All Figures and EV Figures have been uploaded as individual pdf files

2. Data availability: Please data and provide accession codes in this section according the online EMBO author guide (https://www.embopress.org/page/journal/14602075/authorguide#datadeposition). This can include Flow Cytometry results.

   Source data for all graphs, as well as TIFF files for the images has been uploaded as a zip file according to the author guidelines. FCS files for the flow cytometry data has been uploaded to FlowRepository.org with the Repository ID FR-FCM-Z6KY. Please note we do not mention source data information in the manuscript as we wouldn't sure if this information would be added by the editorial staff.

3. The funding information is missing in EJP, please add this.

   This has been added at the submission website

4. Please remove the author contribution section from the manuscript.

   This has been removed, and author contributions were added at the submission website according to the Credit system

5. Please review our new policy on conflict of interests on the EMBO author guide website and update the title of this section to: Disclosure and competing interests statement.

   This statement has been added

6. The paper explained: EMBO Molecular Medicine articles are accompanied by a summary of the articles to emphasize the major findings in the paper and their medical implications for the non-specialist reader. Please provide a draft summary of your article highlighting: the medical issue you are addressing, the results obtained and, their clinical impact. This may be edited to ensure that readers understand the significance and context of the research. Please refer to any of our published articles for an example.

   A Paper Explained section was added to the main manuscript

7. For more information: There is space at the end of each article to list relevant web links for further consultation by our readers. Could you identify some relevant ones and provide such information as well? Some examples are patient associations, relevant databases, OMIM/proteins/genes links, author's websites, etc...

   We did not add any "for more information" as we didn't feel there was any additional relevant information to add here

8. For references, please provide these in alphabetical order and only ten author names should be displayed. In cases where there are more than 10 authors, add et al. after the 10th author name.

   The references have been reformatted to journal standards

9. Please add a table of contents to the appendix file and rename the figures with "Appendix Figure S1" etc. or Appendix Table S1". In figure call outs in the main text, please also update the renamed figures accordingly.

   An appendix file has been created with the remaining supplementary information (after removal of the figures). A table of contents has been added, and all text in the manuscript and appendix has been edited to refer to the Appendix material (and not as supplementary material).

10. We require the publication of source data, particularly for electrophoretic gels and blots and graphs, with the aim of making primary data more accessible and transparent to the reader. Please provide this according to the email from Hannah Sonntag.

    This has been included in the source data file.

11. We do not allow "data not shown" Please remove this reference from p12 and p15.

    All mentions of data not shown have been removed.

12. Figure 3Ei is not called out, please add a reference to this in the correct order in the main manuscript.

    3Ei has been cited in the main text

13. Please remove the list of abbreviations.

    The list of abbreviations has been removed

14. Please move the materials and methods section after the Discussion section.

    The materials and methods section has been moved

15. We include a synopsis of the paper (see http://emboj.embopress.org/). Please provide me with a general summary statement and 3-5 bullet points that capture the key findings of the paper.

    A synopsis was added to the main manuscript file

16. We also need a summary figure for the synopsis. The size should be 550 wide by 200-440 high (pixels). You can also use something from the figures if that is easier.

    A synopsis image was uploaded to the website

17. Our publisher has also done their pre-publication check on your manuscript. I've attached the Data Edited Manuscript file. Please take a look at the word file and the comments regarding the figure legends and respond to the issues.

    The comments in the Data Edited Manuscript file has been responded to. Namely, "biological replicates" was added to the text where appropriate.

Additional requests that were not addressed above (redundant requests omitted):

When submitting your revised manuscript, please include:

1) a .docx formatted version of the manuscript text (including Figure legends and tables)

Done

2) Separate figure files*

Done (as pdfs)

4) a letter INCLUDING the reviewer's reports and your detailed responses to their comments (as Word file).

No response to the reviewer comments was necessary, and so this document includes the response to the editor's checklist

8) EMBO Molecular Medicine now requires a complete author checklist (https://www.embopress.org/page/journal/17574684/authorguide) to be submitted with all revised manuscripts. Please use the checklist as guideline for the sort of information we need WITHIN the manuscript. The checklist

should only be filled with page numbers were the information can be found. This is particularly important for animal reporting, antibody dilutions (missing) and exact values and n that should be indicted instead of a range.

The checklist has been completed. Please note that statements regarding exclusion criteria were added to the materials and methods for the relevant experiments.

11) Please note that we now mandate that all corresponding authors list an ORCID digital identifier. This takes <90 seconds to complete. We encourage all authors to supply an ORCID identifier, which will be linked to their name for unambiguous name identification.

All authors have a unique ORCID identifier. However, it was impossible to edit the authors to add their ORCID identifies as the edit box was greyed out and unclickable. I list them here for completeness.

Erika Varady 0000-0001-8125-0033
Luis Angel Ayala 0000-0002-6272-3314
Pauline Nguyen 0009-0000-0351-0653
Vanessa Scarfone 0000-0001-8786-4191
Alborz Karimzadeh 0000-0002-0846-8981
Cuiwen Zhou 0000-0001-7584-8653
Xiyu Chen 0009-0007-6314-5468
Scott Greilach 0000-0002-5640-8115
Craig Walsh 0000-0001-7808-2817
Matt Inlay 0000-0002-0451-2076

Other changes of note:

The title was shortened to fit within the character limit
The abstract was shortened to fit within the word limit
Files were renamed with the manuscript ID

Changes 6/30/23

Dear Annika Dierderich:

Below is a response to the latest requests (dated 6/30/23). Our responses are in blue font:

1. It is mandatory to include a 'Data Availability' section after the Materials and Methods. Primary datasets produced in this study need to be deposited in an appropriate public database, and the accession numbers and database listed under 'Data Availability'. We were unable to access the data in the FlowRepository, could you please check and provide a reviewer password if the datasets are not yet public (see https://www.embopress.org/page/journal/17574684/authorguide#dataavailability)?

The data availability section as added to the main manuscript after the methods section. It reads: "**Data Availability** The fcs files for the flow cytometry plots presented in the figures are available at FlowRepository.org under the repository ID FR-FCM-Z6KY."

The fcs files are available for reviewer viewing via the following link. We will make the data public once instructed to do so.

http://flowrepository.org/id/RvFr5FH6qO41yb7N1znr29qDhJc73q2NduzlLYThUtNLJuqkzIpCUrSGxRHdc1gj

2. Thank you for uploading the source data files. We noticed that Fig 3B and 4B have not been included -- is this data included in the FACS data deposited in the FlowRepository?

The source data (fcs files) for Fig 3B and 4B are available at the Flow Repository.

3. Could you please upload the synopsis image in jpg or png format, sized 550 pixels wide by 200-440 high?

The synopsis image was resized to 550 x 202 and uploaded as a png file.

4. We were unable to find the appendix file in your submission, could you please upload it?

The appendix has been uploaded

9th Jul 2023

Dear Matt,

I am pleased to inform you that your manuscript is accepted for publication and is now being sent to our publisher to be included in the next available issue of EMBO Molecular Medicine.

If you want to receive an e-mail alert regarding its publication as well as other EMBO Mol Med content, register here: embopress.org/alertsfeeds

Please read below for additional IMPORTANT information regarding your article, its publication and the production process.

Congratulations on your interesting work!

Kelly

Kelly M Anderson, PhD
Scientific Editor
EMBO Molecular Medicine
Follow us on Twitter @EmboMolMed

*** *** *** IMPORTANT INFORMATION *** *** ***

Your article cannot be published until the publisher has received the appropriate signed license agreement. Once your article has been received by Wiley for production you will receive an email from Wiley's Author Services system, which will ask you to log in and will present you with the appropriate license for completion.

Should you be planning a Press Release on your article, please get in contact with embomolmed@wiley.com as early as possible, in order to coordinate publication and release dates.

PROOFS

You will receive the proofs by e-mail approximately 2 weeks after all relevant files have been sent o our Production Office. Please return them within 48 hours and if there should be any problems, please contact the production office at embopressproduction@wiley.com.

Please inform us if there is likely to be any difficulty in reaching you at the above address at that time. Failure to meet our deadlines may result in a delay of publication.

All further communications concerning your paper proofs should quote reference number EMM-2023-17748-V2 and be directed to the production office at embopressproduction@wiley.com.
